# Chemoenzymatic synthesis and biological evaluation of enantiomerically enriched 1-(β-hydroxypropyl)imidazolium- and triazolium-based ionic liquids

**DOI:** 10.3762/bjoc.9.56

**Published:** 2013-03-12

**Authors:** Paweł Borowiecki, Małgorzata Milner-Krawczyk, Jan Plenkiewicz

**Affiliations:** 1 Warsaw University of Technology, Faculty of Chemistry, Noakowskiego St. 3, 00-664 Warsaw, Poland

**Keywords:** antibiotics, antifungal agents, double derivatization, enzyme catalysis, ionic liquids

## Abstract

Racemic 1-(β-hydroxypropyl)azoles were prepared by solvent-free direct regioselective ring opening of 1,2-propylene oxide with imidazole or 1,2,4-triazole. Lipase-catalyzed transesterification of alcohols with vinyl acetate resulted in kinetic enantiomers resolution. Separated (*S*)-enantiomers of (+)-1-(1*H*-imidazol-1-yl)propan-2-ol and (+)-1-(1*H*-1,2,4-triazol-1-yl)propan-2-ol were quaternized with alkyl bromides or iodides, yielding novel optically active ionic liquids. Racemic salts were tested against a wide range of microorganisms.

## Introduction

Aromatic heterocycles play a crucial role in medicinal chemistry [1–2]. More than half of all known drugs contain at least one heterocyclic component. Novel biologically active compounds are often designed as analogues of endogenous ligands that are vital to biochemical processes. Since most of these substances are comprised of heterocycles, such rings by default become core structures of the newly designed therapeutically active compounds.

Many biomolecules (e.g., vitamins, amino acids, purines, alkaloids) containing an imidazole ring [3–5] meet the relevant biological requirements, therefore, its presence in active substances is very desirable. Imidazole rings are also frequently present in antibacterial [6–8], antifungal [9–13], antiparasitic [14–15], anticancer [16–17] and antiaggregatory [18] preparations.

In turn, many 1,2,4-triazole derivatives exhibit antimicrobial [19–20], antifungal [21], antitumor [22], analgesic [23], anti-inflammatory [24], psychoactive [25–27], anticonvulsant [28], diuretic [29], and anti-HIV [30] activity. 1,2,4-Triazole derivatives also represent the most important group of herbicides and fungicides [31].

In recent years growing attention has been focused on imidazole- and 1,2,4-triazole-derived ionic liquids (ILs), as well as their chiral derivatives (CILs). CILs have become a subject of intensive study since their potential as catalysts for asymmetric induction [32–34], as supplements for influencing reaction stereoselectivity [35], as chiral solvents in stereoselective polymerization [36], as chiral phase for gas chromatography [37], or as chiral differentiating solvents for spectroscopic investigations [38–39] is far from being exhausted. CILs can also act as a tool for organizing the structure of solid polymeric electrolytes (SPE), playing the triple role as plasticizer, solvent for the ions, and medium. Most of the described optically active ILs are prepared from easily accessible natural chiral substrates [40]. The use of ILs in so-called “green chemistry”, as for example solvents in inorganic or organic syntheses or as a replacement for many hazardous and volatile organic solvents, raises the question about the toxicity of newly designed compounds. Therefore, it is crucial to select the least toxic ILs with good chemical properties that can be used in industrial processes [41]. Moreover, a number of current studies have demonstrated the potential of certain ionic liquids to exhibit excellent antimicrobial activity, raising the possibility that ionic liquids could find application as biocidal agents in the control of microorganism growth [42–45].

Herein, we report the results of our investigation on the chemoenzymatic synthesis of new chiral ionic liquids using enzyme-catalyzed kinetic resolution of 1-(β-hydroxypropyl)azoles as a key step. We have shown that this attempt is simple and allows the preparation of new types of optically active ionic liquids. Inhibitory activity of the newly synthesized CILs was tested towards gram-negative and gram-positive bacteria and fungi.

## Results and Discussion

### Synthesis and enzymatic resolution of the *N*-2-hydroxypropylazoles

**Scheme 1:**
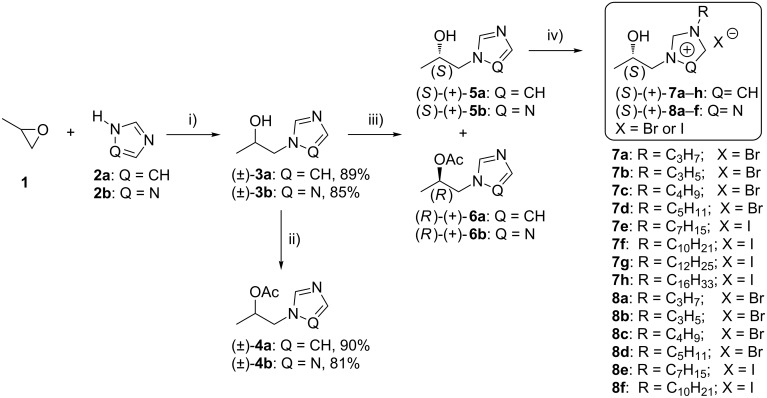
Synthesis of optically active ILs. Reagents and conditions: (i) 1,2-propylene oxide (1.1 equiv), 32 °C, 24 h; (ii) vinyl acetate (5 equiv), Novozyme SP 435, 2-methyl-2-butanol, rt, 48 h, magnetic stirrer; (iii) vinyl acetate (5 equiv), enzyme, 2-methyl-2-butanol/MTBE (methyl *tert*-butyl ether), rt, 250 rpm; (iv) RX (3 equiv), Δ, dry CH_3_CN.

In this paper we report the synthesis of racemic 1-(1*H*-imidazol-1-yl)propan-2-ol and 1-(1*H*-1,2,4-triazol-1-yl)propan-2-ol and the procedure for lipase-catalyzed kinetic separation of their enantiomers. These enantiomers can be used as chiral synthons for new drugs, pesticides or task-specific ionic liquids. The 1-(β-hydroxypropyl)-imidazole (±)-**3a** and -triazole (±)-**3b** used in this study were prepared as racemic mixtures according to the method described by Yus and co-workers [46] (Scheme 1). Next, these compounds were used as a new type of substrate in lipase-catalyzed transesterification, yielding both enantiomers with good optical purity.

The syntheses of the secondary alcohols (±)-**3a** and (±)-**3b** were accomplished by the addition of imidazole (**2a**) or 1,2,4-triazole (**2b**) to 1,2-propylene oxide (**1**) under solvent-free conditions. The epoxide ring-opening reactions were carried for 24 h at elevated temperature (32 °C) and resulted in the formation of the appropriate alcohols (±)-**3a** and (±)-**3b** in high yields (Scheme 1).

In the next step, the influence of crucial parameters in enzyme-catalyzed reactions such as the type and quantity of the biocatalyst and solvent on the enantiomers resolution was investigated. The aim was to achieve a conversion close to 50% and 99% ee for the slow-reacting substrates and product enantiomers. Four different lipases, i.e., two native (Amano PS, Amano AK) and two immobilized enzymes (Novozym SP 435, Amano PS-IM), were chosen for the transesterification studies. The screenings were performed with a 5-fold molar excess of vinyl acetate as an irreversible acyl donor at ambient temperature. The selection of a proper solvent is a significant factor in biocatalysis [47–48], since solvents are able to change the activity [49–51] and enantioselectivity [52–54] of an enzyme. Conformational changes in the enzyme structure under the influence of solvent can even invert the substrate specificity and the enzyme–substrate affinity [55]. The ability to control the direction of an enzymatic reaction and its specificity in a nonaqueous environment has resulted in the development of so-called "solvent engineering". After testing several solvents for lipase-catalyzed acetylation of (±)-**3a** and (±)-**3b** with vinyl acetate, we determined the following order of their utility as the reaction media: MTBE ~ 2-methyl-2-butanol > acetone > 1,4-dioxane >> acetonitrile. Further optimization revealed that MTBE and 2-methyl-2-butanol are unsurpassed, not only from the viewpoint of substrate solubility and reactivity, but also due to the highest enantiomeric excesses of the separated enantiomers. Results of the lipase-catalyzed transesterification of the racemic (±)-**3a** are summarized in Table 1.

**Table 1:** Lipase-catalyzed resolution of racemic 1-(1*H*-imidazol-1-yl)propan-2-ol (±)-**3a**.

Entry	Enzyme	Solvent	*t* (h)	Conv.^a^ (%)	Product	ee^b^ (%)	*E*^c^	Yield^d^ (%)

1	Amano PS	MTBE^e^	20	30	alcohol ester	41 97	98	95 56
2		2-methyl-2-butanol^e^	26	32	alcohol ester	46 99	103	43 67

3	Amano AK	MTBE^e^	5	47	alcohol ester	88 >99	584	97 62
4		2-methyl-2-butanol^e^	22	41	alcohol ester	70 >99	419	95 90
5		2-methyl-2-butanol^f^	43	45	alcohol ester	80 >99	492	94 91

6	Novozym SP 435	MTBE^e^	1.5	57	alcohol ester	97 73	26	95 79
7		MTBE^f^	7	59	alcohol ester	98 68	23	53 73
8		2-methyl-2-butanol^e^	7	30	alcohol ester	40 93	41	99 64
9		2-methyl-2-butanol^f^	11	53	alcohol ester	82 73	16	70 62
10		2-methyl-2-butanol^f^	12	57	alcohol ester	92 68	17	40 60

11	Amano PS-IM	MTBE^f^	3	39	alcohol ester	64 99	386	98 75
12		MTBE^f^	4	46	alcohol ester	82 98	254	66 95
13		2-methyl-2-butanol^f^	5	38	alcohol ester	60 >99	368	86 88

^a^Conversion was calculated from the enantiomeric excess of the starting material (ee_s_) and the product (ee_p_) according to the formula: conv. = ee_s_/(ee_s_ + ee_p_). ^b^Determined by HPLC analysis by using a Chiralcel OD-H column. ^c^Calculated according to Chen et al. [56], by using the equation: *E* = ln[(1 − conv.)(1 − ee_s_)]/ln[(1 − conv.)(1 + ee_s_)]. ^d^Isolated yield after column chromatography. ^e^Conditions: (±)-**3a** 300 mg, lipase 100 mg, solvent 3 ml, vinyl acetate 1 g (5 equiv), 250 rpm at rt. ^f^Conditions: (±)-**3a** 300 mg, lipase 50 mg, solvent 3 ml, vinyl acetate 1 g (5 equiv), 250 rpm at rt.

The experiments demonstrated that the reaction proceeded particularly efficiently when Amano AK or Amano PS-IM were used. These enzymatic preparations also gave excellent reaction enantioselectivities (Table 1, entries 3–5 and 11–13, *E* >> 200). Particularly, Amano AK was characterized by very high selectivity (Table 1, entry 3, *E* = 584) toward the acetate (+)-**6a** formation, which was enantiomerically pure (>99% ee) when the reaction was arrested close to 45% conversion. In turn, conversion exceeding 57% was beneficial for high optical purity of the remaining alcohol (+)-**5a** (Table 1, entries 6 and 7, >97% ee).

In the lipase-catalyzed acetylation of alcohol (±)-**3b** the best results were achieved with native *Pseudomonas fluorescens* lipase (Amano AK) suspended in 2-methyl-2-butanol (Table 2, entry 5, *E* = 56). As shown in the Table 2, the reaction time required for about 50% substrate conversion varied from 5 h for Amano PS-IM in MTBE (Table 2, entry 8) to 132 h for the least active enzyme, i.e., native Amano PS in 2-methyl-2-butanol (Table 2, entry 2).

**Table 2:** Lipase-catalyzed resolution of racemic 1-(1*H*-1,2,4-triazol-1-yl)propan-2-ol (±)-**3b**.

Entry	Enzyme	Solvent	*t* (h)	Conv.^a^ (%)	Product	ee^b^ (%)	*E*^c^	Yield^d^ (%)

1	Amano PS	MTBE^e^	65	40	alcohol ester	57 84	20	77 87
2		2-methyl-2-butanol^e^	132	39	alcohol ester	56 88	27	85 73

3	Amano AK	MTBE^e^	12	41	alcohol ester	63 89	33	97 97
4		2-methyl-2-butanol^e^	30	37	alcohol ester	54 90	33	93 97
5		2-methyl-2-butanol^e^	49	54	alcohol ester	98 85	56	93 97

6	Novozym SP 435	MTBE^e^	7	45	alcohol ester	49 59	6	82 98
7		2-methyl-2-butanol^e^	8	40	alcohol ester	40 60	6	95 93

8	Amano PS-IM	MTBE^f^	5	47	alcohol ester	74 84	25	76 87
9		2-methyl-2-butanol^f^	10	41	alcohol ester	62 90	36	98 77

^a^Conversion was calculated from the enantiomeric excess of the starting material (ee_s_) and the product (ee_p_) according to the formula conv. = ee_s_/(ee_s_ + ee_p_). ^b^Determined by HPLC analysis by using Chiralcel OD-H column. ^c^Calculated according to Chen et al. [56], by using the equation: *E* = ln[(1 − conv.)(1 − ee_s_)]/ln[(1 - conv.)(1 + ee_s_)]. ^d^Isolated yield after column chromatography. ^e^Conditions: (±)-**3a** 300 mg, lipase 100 mg, solvent 3 ml, vinyl acetate 1 g (5 equiv), 250 rpm at rt. ^f^Conditions: (±)-**3a** 300 mg, lipase 50 mg, solvent 3 ml, vinyl acetate 1 g (5 equiv), 250 rpm at rt.

Generally, similar to acetylation of (±)-**3a**, the highest reaction rates for (±)-**3b** were observed with Amano PS-IM in MTBE. As usual, the optical purities of the acetate, as well as of the remaining alcohol, were dependent on the enzyme used and the conversion rate. For example, with Amano AK the reaction reached 37% conversion after 30 h giving product (+)-**6b** in 90% ee (Table 2, entry 4), and after 49 h the stereochemical course reached 54% conversion yielding the slower reacting enantiomer (+)-**5b** with high enantiomeric excess (Table 2, entry 5, 98% ee). The highest optical purity for (+)-**6b** (90% ee) and the shortest reaction time (10 h) were obtained by the reaction catalyzed by Amano PS-IM, which was much faster than that catalyzed by native Amano PS. The reaction enantioselectivities were fairly good (*E* = 20–56) for all of the tested lipases except Novozyme SP 435, which showed very high activity but low stereoselectivity (*E* = 6).

The racemic acetyl esters (±)-**4a** and (±)-**4b** used for determination of the enantiomeric configurations were prepared in good isolated yields by lipase-catalyzed esterification of the appropriate 1-(β-hydroxypropyl)azole (±)-**3a** or (±)-**3b** with vinyl acetate as the acyl donor (Scheme 1). The enzyme-catalyzed syntheses of acetylated standards were used, since the conventional esterification procedure of (±)-**3a** and (±)-**3b** (with Ac_2_O, pyridine and DMAP) gave low yields (ca. 25%) of the acetates.

### Determination of the stereochemistry of alcohols (+)-**5a** and (+)-**5b**

**Scheme 2:**
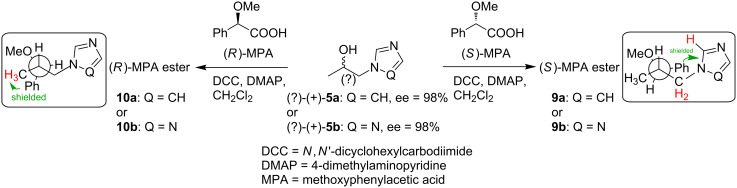
Conversion of (+)-**5a** and (+)-**5b** into MPA esters **9a**, **9b** and **10a**, **10b**.

The absolute configurations of the alcohols (+)-**5a** and (+)-**5b**, obtained by lipase-catalyzed transesterification of the racemates (±)-**3a** and (±)-**3b**, were determined by the modified Mosher’s method as described by Riguera et al [57]. This approach consists of comparing the differences between ^1^H NMR chemical shifts recorded for the diastereomeric esters prepared from the separated enantiomers of the alcohols (+)-**5a** or (+)-**5b** and (*R*)- and (*S*)-enantiomers of methoxyphenylacetic acid (MPA, Mosher reagent) (Scheme 2). The utility of this method applied to secondary alcohols possessing heterocyclic azole rings has been demonstrated by us previously [58].

The absolute configuration of the substrate is deduced by interpretation of the signs of the Δδ*^RS^* values, by using an empirical model that assumes that in MPA esters of secondary alcohols, the most representative conformer has the methoxy group of MPA, the carbonyl group, and a proton bonded to the stereogenic center of the alcohol in the same plane.

The differences in the chemical shifts (Δδ*^RS^*) observed in the spectra of the esters prepared from the chiral auxiliaries [(*R*)- and (*S*)-MPA acids, respectively], were calculated separately for the protons attached to the carbon atoms situated on both sides of the chirality center, as shown by the following equations:


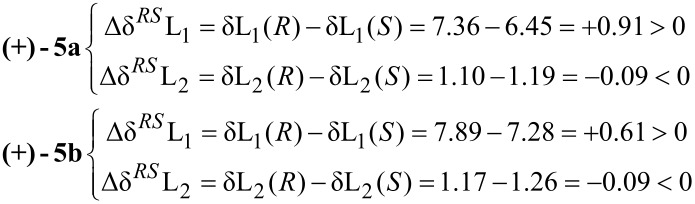


**Figure 1:**
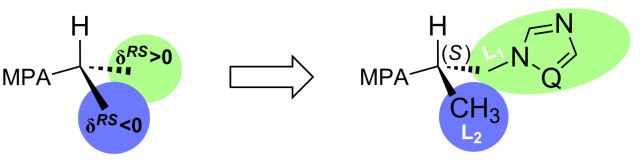
Model for the configurational correlation of MPA esters **9a**, **9b** and **10a**, **10b**.

**Figure 2:**
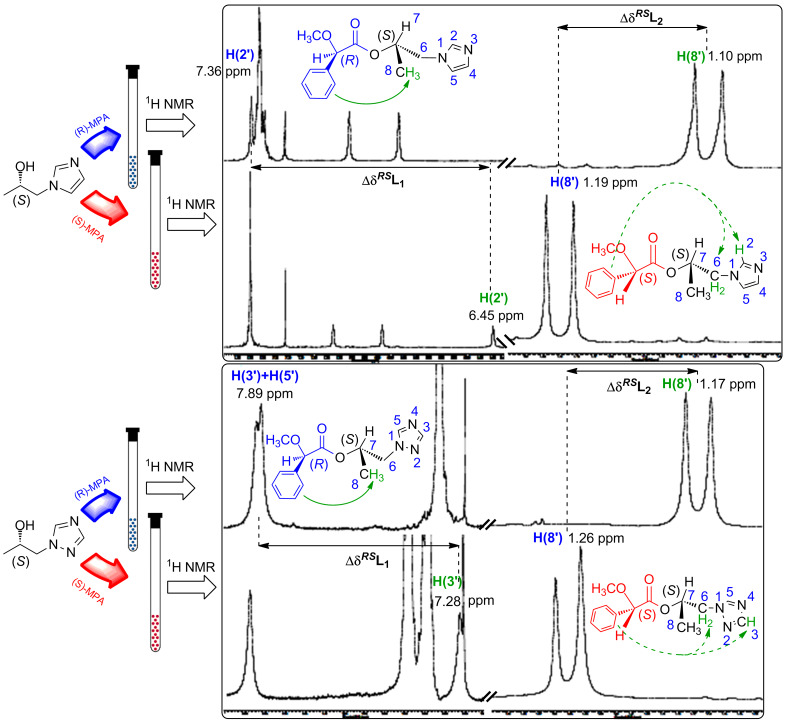
The ^1^H NMR spectra of the derivatives of two unreacted chiral alcohols (+)-**5a** (top) and (+)-**5b** (bottom). Symbols marked in green represent protons shielded by the phenyl ring of chiral auxiliary (MPA); blue labels stand for unaffected protons.

The signs of these parameters bear the information necessary for the configurational assignment, since they indicate the relative position of L1/L2, with respect to the anisotropic group (phenyl group of MPA). The positive value of Δδ*^RS^*, which corresponds to the signal of the protons of the substituent L1, and the negative sign for the protons L2 indicate an (*S*)-configuration for the enantiomers of both types of investigated alcohols (+)-**5a** and (+)-**5b** according to Figure 1. The ^1^H NMR spectra recorded for the resulting diastereomeric derivatives (Figure 2) confirmed the results of the above equations.

The chiral environment provided by the derivatizing agent (MPA) covalently associated with the respective alcohol leads to significant differences in chemical shifts (δ = 0.91 ppm), especially in the case of the imidazolium derivatives **9a** and **10a**. Comparison of the ^1^H NMR spectra of these two species for this particular alcohol (+)-**5a** shows that the phenyl ring of the chiral auxiliary reagent projects its magnetic anisotropy strongly toward the H(2’) proton in the (*S*)-derivative **9a**, while the same proton in the (*R*)-MPA ester **10a** remains unaffected. The opposite phenomenon is observed for methyl group protons H(8’), which are shielded in (*R*)-MPA derivatives **10a** due to the strength and direction of the anisotropic effect, while in (*S*)-MPA ester **9a** they are unaffected. Similarly, information gained from ^1^H NMR spectra of both species of triazolic alcohol, **9b** and **10b**, defined (+)-**5b** as having (*S*)-configuration (see the lower part of the Figure 2).

### Synthesis of chiral ionic liquids

In the final step, the appropriate chiral intermediates (*S*)-(+)-1-(1*H*-imidazol-1-yl)propan-2-ol ((+)-**5a**) or (*S*)-(+)-1-(1*H*-1,2,4-triazol-1-yl)propan-2-ol ((+)-**5b**) and various alkyl halides were reacted in dry acetonitrile. The quaternization of the *N*-3 atom of imidazole and that of *N*-4 of the 1,2,4-triazole ring of the derivatives, provided salts (+)-**7a**–**h** and (+)-**8a**–**f** in >81% and >78% isolated yields, respectively (Table 3). With the exception of (+)-**8a**–**c** and (+)-**8e**–**f**, which are exceedingly viscous liquids (gums), all of the chiral hydroxy-functionalized imidazolium and one of the triazolium salts are liquid at room temperature. Final products were characterized by ^1^H, ^13^C NMR and FTIR spectroscopy as well as high-resolution electrospray ionization mass spectrometry (HRMS–ESI). Their structures were found to be spectroscopically well–defined, which was further confirmed by elemental analyses. For the purpose of biological tests, the racemic mixtures all of salts presented in Table 3 were synthesized.

**Table 3:** Synthesis of chiral imidazolium (+)-**7a**–**h** and triazolium ILs (+)-**8a**–**f**.

Entry	IL	R	X	*t* (h)	*T* (°C)	Yield (%)	[α]_D_^a^

1	(+)-**7a**	C_3_H_7_	Br	96	65	92	+23.6
2	(+)-**7b**	C_3_H_5_	Br	96	65	93	+25.3
3	(+)-**7c**	C_4_H_9_	Br	96	80	94	+17.2
4	(+)-**7d**	C_5_H_11_	Br	96	82	82	+15.4
5	(+)-**7e**	C_7_H_15_	I	96	82	92	+16.3
6	(+)-**7f**	C_10_H_21_	I	96	82	92	+15.8
7	(+)-**7g**	C_12_H_25_	I	96	120	86	+14.2
8	(+)-**7h**	C_16_H_33_	I	96	120	81	+11.4

9	(+)-**8a**	C_3_H_7_	Br	48	65	98	+18.8
10	(+)-**8b**	C_3_H_5_	Br	48	65	94	+15.6
11	(+)-**8c**	C_4_H_9_	Br	48	80	90	+17.7
12	(+)-**8d**	C_5_H_11_	Br	48	82	78	+15.4
13	(+)-**8e**	C_7_H_15_	I	48	82	87	+10.2
14	(+)-**8f**	C_10_H_21_	I	48	82	92	+8.5

^a^Specific rotation; *c* solution in chloroform (*c* 1.0).

### Antimicrobial activity of racemic ionic liquids

Antibacterial and antifungal activities of the synthesized racemic CILs were evaluated against a wide range of microorganisms. The studies were conducted on three strains of gram-negative bacteria, two strains of gram-positive bacteria, and eight strains of fungi. For the preliminary screening of bacteria and yeasts we used the agar diffusion test (Table 4) in order to select the most promising compounds for further determination of minimal inhibitory concentrations (MICs) by the broth dilution method (Table 5). In turn, the antifungal activity was tested by the agar dilution method. The results are listed in Table 6.

**Table 4:** Growth inhibition halo (cm) for the racemic imidazolium and triazolium CILs (25 mM).

CIL	R	*E. coli* ATCC 8739 G(−)	*S. typhimurium* ATCC 14028 G(−)	*P. aeruginosa* ATCC 9027 G(−)	*B. subtilis* ATCC 6633 G(+)	*S. aureus* ATCC 6538 G(+)	*C. albicans* ATCC 10231 yeast

(±)-**7a**	C_3_H_7_	–	–	–	–	–	–
(±)-**7d**	C_5_H_11_	–	–	–	–	–	–
(±)-**7e**	C_7_H_15_	1.20 ± 0.00	1.03 ± 0.04	1.05 ± 0.00	1.20 ± 0.00	1.73 ± 0.04	–
(±)-**7f**	C_10_H_21_	1.40 ± 0.00	1.23 ± 0.04	1.10 ± 0.00	2.30 ± 0.00	2.80 ± 0.00	+

(±)-**8a**	C_3_H_7_	–	–	–	–	–	–
(±)-**8d**	C_5_H_11_	–	–	–	–	–	–
(±)-**8e**	C_7_H_15_	–	–	–	–	1.00 ± 0.00	–
(±)-**8f**	C_10_H_21_	1.30 ± 0.14	1.25 ± 0.07	1.08 ± 0.04	1.90 ± 0.14	1.25 ± 0.07	1.18 ± 0.04

–: No inhibition; +: total growth inhibition

**Table 5:** The MIC (mM) values for the racemic imidazolium and triazolium CILs.

CIL	R	*E. coli* ATCC 8739 G(−)	*S. typhimurium* ATCC 14028 G(−)	*P. aeruginosa* ATCC 9027 G(−)	*B. subtilis* ATCC 6633 G(+)	*S. aureus* ATCC 6538 G(+)	*C. albicans* ATCC 10231 yeast

(±)-**7f**	C_10_H_21_	0.5	0.4	>9.5	0.5	0.2	0.3
(±)-**7g**	C_12_H_25_	0.3	0.5	1.6	0.5	0.3	0.1
(±)-**7h**	C_16_H_33_	0.2	0.5	9.0	0.2	0.3	0.3

(±)-**8f**	C_10_H_21_	1.7	4.8	5.5	3.0	0.5	0.9

**Table 6:** Antifungal activity (% of control) of the racemic imidazolium and triazolium CILs (1mM).

CIL	R	*F. oxysporum* MF 5	*F. sambucinum* MF 1	*F. culmorum* MF 18	*A. brasiliensis* ATCC 16404	*C. coccodis* MC 1	*P. infestans* MP 324	*P. infestans* MP 1320

(±)-**7a**	C_3_H_7_	89.97 ± 0.00	99.60 ± 0.88	98.38 ± 0.81	100.78 ± 0.78	98.70 ± 1.67	98.81 ± 3.76	93.00 ± 7.87
(±)-**7d**	C_5_H_11_	90.12 ± 5.10	96.56 ± 3.82	101.32 ± 1.02	92.89 ± 1.18	96.71 ± 1.58	99.49 ± 5.01	104.17 ± 7.04
(±)-**7e**	C_7_H_15_	110.20 ± 4.01	87.55 ± 8.03	82.73 ± 0.66	89.92 ± 5.47	96.55 ± 1.24	90.65 ± 2.14	59.29 ± 3.20
(±)-**7f**	C_10_H_21_	59.70 ± 5.12	63.00 ± 1.45	68.40 ± 1.00	91.55 ± 0.68	99.54 ± 1.76	113.32 ± 3.17	99.43 ± 4.21
(±)-**7g**	C_12_H_25_	47.58 ± 7.08	61.33 ± 1.50	66.78 ± 3.68	87.20 ± 0.72	93.73 ± 1.97	88.21 ± 6.76	85.02 ± 5.16
(±)-**7h**	C_16_H_33_	74.35 ± 11.65	94.53 ± 3.72	74.13 ± 8.51	93.66 ± 2.96	103.10 ± 45.34	92.77 ± 2.65	91.87 ± 2.28

(±)-**8a**	C_3_H_7_	95.16 ± 3.07	102.28 ± 0.76	99.19 ± 2.04	98.40 ± 3.45	101.30 ± 1.67	101.58 ± 2.37	91.20 ± 6.68
(±)-**8d**	C_5_H_11_	94.65 ± 3.43	93.89 ± 5.06	105.29 ± 2.64	93.31 ± 1.60	97.53 ± 6.64	112.05 ± 0.00	93.05 ± 24.81
(±)-**8e**	C_7_H_15_	105.10 ± 2.32	98.39 ± 5.47	95.58 ± 2.78	104.12 ± 1.58	99.82 ± 3.43	104.63 ± 7.64	92.64 ± 4.45
(±)-**8f**	C_10_H_21_	77.35 ± 5.95	73.58 ± 5.28	92.64 ± 7.14	98.65 ± 3.12	104.59 ± 2.59	93.47 ± 2.87	95.58 ± 4.82

The antimicrobial and antifungal activities of tested CILs are significantly dependent on the alkyl chain length, as was demonstrated in numerous previous studies [59]. The high toxicity of CILs was noted for CILs with alkyl-chain substituents of 10–16 carbon atoms (Table 4 and 5). Moreover, the chemical nature of the cationic head group influenced the overall toxicity of the CILs, which is in good agreement with several previous studies [60]. The imidazolium CILs exhibited visibly stronger antibacterial and antifungal activity than triazolium CILs (Tables 4–6). However, the filamentous fungi displayed high tolerance towards the tested CILs. Nevertheless, the relation between the alkyl chain substituent and the antifungal activity of the tested compounds could still be observed, with the strongest toxicity demonstrated by CILs with an alkyl chain substituent of 12 carbon atoms (Table 6). This high tolerance of filamentous fungi against CILs could be explained by the ability of fungal cultures to change their cell biochemistry (resulting in an altered pattern of secondary metabolites) in response to CILs [61].

## Conclusion

Lipase-catalyzed kinetic enantiomeric separation of 1-(1*H*-imidazol-1-yl)propan-2-ol (±)-**3a** proceeded with excellent enantioselectivity, exceeding *E* = 500, in a short reaction time (5 h), by using a native enzymatic preparation from *Pseudomonas fluorescens* (Amano AK) as biocatalyst. In turn, after many trials we found that the kinetic separation of enantiomers of 1-(1*H*-1,2,4-triazol-1-yl)propan-2-ol (±)-**3b** with various tested lipases was less efficient than that of (±)-**3a**. Resolution of (±)-**3b** proceeded with good yield, but the enantiomeric excess of the slower reacting enantiomer (alcohol (+)-**5b**) was less than 98%. The faster reacting ester (+)-**6b** barely reached 90% ee. Nevertheless, the procedure presented here is simple and efficient, and, after some additional optimization, can be readily extended to other substrates of this type. According to ^1^H NMR investigations, the slower reacting enantiomers of the alcohols (+)-**5a** and (+)-**5b** possess (*S*)-configuration. This assignment is in good agreement with Kazlauskas’s rule [62–63], where in lipase-catalyzed esterification of secondary alcohols the (*R*)-ester and (*S*)-alcohol enantiomers are obtained. The imidazolic and triazolic chiral salts derived from alcohols (S)-(+)-**5a** and (S)-(+)-**5b** were obtained in high to excellent isolated yields. The core structures of these salts were modified by using, for quaternization reaction, haloalkanes or haloalkenes with various chain lengths. Their antibacterial and antifungal properties were evaluated by three different methods. Some of these compounds exhibited biological activity that was significantly dependent on the alkyl chain length, with considerably high toxicity of the substituents with 10–16 carbon atoms. The imidazolium salts revealed stronger antibacterial activity than their triazolium analogues.

## Supporting Information

File 1 Complete experimental procedures and characterization data.
