# Supplementary material for: Chemoenzymatic synthesis and biological evaluation of enantiomerically enriched 1-(β-hydroxypropyl)imidazolium- and triazolium-based ionic liquids
Source: Beilstein J Org Chem. 2013 Mar 12;9:516–25. doi: 10.3762/bjoc.9.56 (PMC3628848; doi:10.3762/bjoc.9.56)
Supplement: File 1 — Complete experimental procedures and characterization data. [file Beilstein_J_Org_Chem-09-516-s001.pdf]

Supporting Information

for

Chemoenzymatic synthesis and biological evaluation of enantiomerically enriched 1-(β-hydroxypropyl)imidazolium- and triazolium-based ionic liquids

Paweł Borowiecki*, Małgorzata Milner-Krawczyk and Jan Plenkiewicz

Address: Warsaw University of Technology, Faculty of Chemistry, Noakowskiego St. 3, 00-664 Warsaw, Poland

Email: Paweł Borowiecki* - pawel_borowiecki@onet.eu

* Corresponding author

Content

General details S2–S3

Complete experimental procedures and characterization data S3–S21

Copies of HPLC chromatograms and spectra S22–S72

References ……………………………………..S72

General details

All commercially available reagents (Aldrich, Fluka and POCH) were used without further purification. Novozym SP 435 (lipase from *Candida antarctica B* immobilized on a macroporous acrylic resin, >10000 U/g), Amano PS (native lipase from *Burkholderia cepacia ealier Pseudomonas cepacia*, >23.000 U/g), Amano PS-C (immobilized on ceramic particles) and Amano PS-IM (immobilized on diatomite, >500 U/g), Amano AK (native lipase from *Pseudomonas fluorescens*, >20.000 U/g) were purchased from Novo Nordisk Co. and Amano Pharmaceutical Co., respectively and were used without any pretreatment. Melting points were obtained with an MPA100 Optimelt SRS apparatus. Thin-layer chromatography was carried on TLC aluminium plates with silica gel Kieselgel 60 F_{254} (Merck) (0.2 mm thickness film) and the compounds were visualized in iodine vapor. Preparative plate chromatography was performed with PSC-Fertigplatten Kieselgel 60 F_{254} (20 × 20 cm with 2 mm thickness layer). The chromatographic analyses (GLC) were performed with an HP Series II 5890 instrument equipped with a flame ionization detector (FID) and fitted with HP-50+ (30 m) semipolar column. Helium (2 ml/min) was used as carrier gas; T_{injector} = 280 °C, T_{column} = 100 °C (3 min) and 100–280 °C (10 °C/min); retention times (t_R) are given in minutes under these conditions. Column chromatography was performed by using Silica gel 60 (Merck) of 40–63 μm. A mixture of chloroform/methanol was used as eluent (9:1 or 8:2 v/v depending on substance purified). The enantiomeric excesses of the resulting esters and alcohols were determined by HPLC analysis performed on Shimadzu CTO-10ASV chromatograph equipped with STD-20A UV detector and Chiralcel OD-H (Diacel) chiral column using mixtures of *n*-hexane/isopropyl alcohol as the mobile phase in appropriate ratios given in experimental section; flow (*f*) is given in ml/min; racemic alcohols and esters

were used as standards. Optical rotations were measured with a P20 polarimeter (Belligham & Stanley Ltd., line D spectrum of sodium) in a 2 dm long cuvette. UV–vis absorption spectra were measured with Cary 3 spectrometer (data were collected on diluted solutions in quartz spectroscopy cells). ^{1}H and ^{13}C NMR spectra were measured with a Varian Mercury 400BB spectrometer operating at 400 MHz for ^{1}H and 100 MHz for ^{13}C nuclei, chemical shifts (δ) are given in parts per million (ppm) related to tetramethylsilane (TMS) as internal standard; signal multiplicity assignment: s, singlet; d, doublet; t, triplet; q, quartet; m, multiplet; coupling constant (*J*) are given in hertz (Hz). Mass spectra were recorded with a Micro-mass ESI Q-TOF spectrometer at the Mass Spectrometry Laboratory, Institute of Biochemistry and Biophysics (IBB), PAN. IR spectra of neat samples were recorded on a PerkinElmer System 2000 FT-IR Spectrometer equipped with a Pike Technologies GladiATR^{TM} attenuated total reflectance (ATR) accessory with a monolithic diamond crystal stage and a pressure clamp. Elemental analyses were performed with a VARIO EL III (Elementar Analysensysteme GmbH) elemental analyzer.

Procedures for gram-scale preparation of racemic alcohols and acetates

1-(1*H*-Imidazol-1-yl)propan-2-ol (±)-3a: A mixture of imidazole (8 g; 117.52 mmol) and 1,2-propylene oxide (7.5 g; 129.28 mmol; 9 ml) was placed in a round-bottomed flask and stirred at 32 °C for 24 h. The resulting crude product was purified by column chromatography [$CHCl_3$–MeOH (9:1)] yielding a yellowish oil (13.2 g; 104.6 mmol; yield = 89%). R_f = 0.41 [$CHCl_3$–MeOH (9:1)]; ^{1}H NMR ($CDCl_3$, 400 MHz) δ 1.17 (d, *J* = 8 Hz, 3H, CH_3CH(OH)CH_2), 3.77–3.91 (m, 2H, HOCHCH_2N^1), 3.98–

4.01 (m, 1H, HOC*H*CH_2N^1), 5.69 (s, 1H, O*H*), 6.83 (s, 1H, CH_2N^1CH=C*H*N^3), 6.87 (s, 1H, CH_2N^1C*H*=CHN^3), 7.32 (s, 1H, CH_2N^1C*H*=N^3); ^{13}C NMR ($CDCl_3$, 100 MHz) δ 20.5 (*C*H_3CH(OH)CH_2), 54.6 (HOCH*C*H_2N^1), 66.3 (HO*C*HCH_2N^1), 119.7 (CH_2N^1*C*H=CHN^3), 128.1 (CH_2N^1CH=*C*HN^3), 137.3 (CH_2N^1*C*H=N^3); HRMS (ESI$^+$, *m*/*z*): $[M + H]^+_{calcd}$ = 127.0865, $[M + H]^+_{found}$ = 127.1009; anal. calcd. (%) for $C_6H_{10}N_2O$: C, 57.12 H, 7.99 N, 22.21; found: C, 57.16 H, 7.98 N, 22.25; IR (neat, ν cm^{-1}): 3111, 2971, 2869, 1734, 1509, 1437, 1373, 1285, 1233, 1107, 1076, 919, 820, 735, 661, 625, 472; UV–vis: λ_{max} = 201 nm; GC: t_R = 5.960; HPLC [hexane–iPrOH (9:1); f = 0.8]: t_R = 20.056, 34.482.

1-(1*H*-1,2,4-Triazol-1-yl)propan-2-ol (±)-3b: A mixture of 1,2,4-triazole (7 g; 101.36 mmol) and 1,2-propylene oxide (6.47 g; 111.49 mmol; 7.8 ml) was placed in a round-bottomed flask and stirred at 32 °C for 24 h. The resulting crude product was purified by column chromatography [CHCl3–MeOH (4:1)] to afford a yellowish oil (10.9 g; 85.7 mmol; yield = 85%). R_f = 0.42 [$CHCl_3$–MeOH (4:1)]; ^{1}H NMR ($CDCl_3$, 400 MHz) δ 1.11 (d, *J* = 8 Hz, 3H, C*H*$_3$CH(OH)CH_2), 3.85–3.94 (m, 1H, HOC*H*CH_2N^1), 3.98–4.10 (m, 2H, HOCHC*H*$_2N^1$), 5.00 (s, 1H, O*H*), 7.66 (s, 1H, $CH_2N^1N^2$=C*H*N^4), 7.98 (s, 1H, CH_2N^1C*H*=N^4); ^{13}C NMR ($CDCl_3$, 100 MHz) δ 20.17 (*C*H_3CH(OH)CH_2), 56.6 (HOCH*C*H_2N^1), 65.3 (HO*C*HCH_2N^1), 143.5 (CH_2N^1*C*H=N^4), 150.9 ($CH_2N^1N^2$=*C*HN^4); HRMS (ESI$^+$, *m*/*z*): $[M + H]^+_{calcd}$ = 128.0818, $[M + H]^+_{found}$ = 128.1116; anal. calcd. (%) for $C_5H_9N_3O$: C, 47.23 H, 7.13 N, 33.05; found: C, 47.27 H, 7.13 N, 33.07; IR (neat, ν cm^{-1}): 3268, 3116, 2971, 1508, 1434, 1375, 1274, 1210, 1188, 1134, 1077, 1012, 938, 844, 752, 678, 638, 472; UV–vis: λ_{max} = 201 nm; GC: t_R = 4.379; HPLC [hexane–iPrOH (9:1); f = 0.8]: t_R = 14.716, 17.830.

1-(1*H*-Imidazol-1-yl)propan-2-yl acetate (±)-4a: To a solution of 1-(1*H*-imidazol-1-yl)propan-2-ol (±)-**3a** (0.3 g; 2.38 mmol) in 2-methyl-2-butanol (2.5 ml), vinyl acetate (1.02 g; 11.89 mmol; 1.1 ml) and Novozym SP 435 (0.1 g) were added. The reaction mixture was stirred at room temperature with a magnetic stirrer for 48 h. Aliquots were regularly analyzed by GC until almost 100% substrate conversion was reached. The enzyme was removed by filtration, and the solution was evaporated to dryness. The crude product was purified by chromatography [$CHCl_3$–MeOH (9:1)] to afford a yellowish oil (0.36 g; 2.14 mmol; yield = 90%). R_f = 0.55 [$CHCl_3$–MeOH (9:1)]; ^{1}H NMR ($CDCl_3$, 400 MHz) δ 1.14 (d, *J* = 6.24 Hz, 3H, $CH_3CH(OH)CH_2$), 1.94 (s, 3H, CH_3C=O), 3.90–4.03 (m, 2H, $HOCHCH_2N^1$), 5.01–5.05 (m, 1H, $HOCHCH_2N^1$), 6.85 (s, 1H, CH_2N^1CH=CHN^3), 6.97 (s, 1H, CH_2N^1CH=CHN^3), 7.43 (s, 1H, CH_2N^1CH=N^3); ^{13}C NMR ($CDCl_3$, 100 MHz) δ 16.9 ($CH_3CH(OH)CH_2$), 20.8 (CH_3C=O), 50.73 ($HOCHCH_2N^1$), 68.9 ($HOCHCH_2N^1$), 119.4 (CH_2N^1CH=CHN^3), 128.8 (CH_2N^1CH=CHN^3), 137.4 (CH_2N^1CH=N^3), 169.8 (*C*=O); HRMS (ESI$^+$, *m*/*z*): $[M + H]^+_{calcd}$ = 169.0971, $[M + H]^+_{found}$ = 169.1240; anal. calcd. (%) for $C_8H_{12}N_2O_2$: C, 57.13 H, 7.19 N, 16.66; found: C, 57.10 H, 7.20 N, 16.63; UV–vis: λ_{max} = 201 nm; GC: t_R = 6.735; HPLC [hexane–iPrOH (9:1); f = 0.8]: t_R = 32.938, 39.637.

1-(1*H*-1,2,4-Triazol-1-yl)propan-2-yl acetate (±)-4b: To the solution of 1-(1*H*-1,2,4-triazol-1-yl)propan-2-ol (±)-**3b** (0.3 g; 2.36 mmol) in 2-methyl-2-butanol (2.5 ml), vinyl acetate (1.02 g; 11.79 mmol; 1.1 ml) and Novozym SP 435 (0.1 g) were added. The reaction mixture was stirred at room temperature with a magnetic stirrer for 48 h. Aliquots were regularly analyzed by GC until almost 100% substrate conversion was reached. The reaction was stopped by draining of the enzyme. After solvent evaporation the crude product was purified by chromatography [$CHCl_3$–MeOH (9:1)]

to yield product as a yellowish oil (0.32 g; 1.9 mmol; yield = 81%). R_f = 0.64 [$CHCl_3$–MeOH (9:1)]; ^{1}H NMR ($CDCl_3$, 400 MHz) δ 1.14 (d, J = 6.46 Hz, 3H, CH_3CH(OH)CH_2), 1.87 (s, 3H, CH_3C=O), 4.11–4.26 (m, 2H, HOCHC$H_2$$N^1$), 5.08–5.13 (m, 1H, HOC*H*$CH_2$$N^1$), 7.80 (s, 1H, $CH_2N^1N^2$=C*H*N^4), 7.99 (s, 1H, CH_2N^1C*H*=N^4); ^{13}C NMR ($CDCl_3$, 100 MHz) δ 17.0 (*C*H_3CH(OH)CH_2), 20.7 (*C*H_3C=O), 53.1 (HOCH*C*$H_2$$N^1$), 68.3 (HO*C*H$CH_2$$N^1$), 143.5 ($CH_2N^1$*C*H=$N^4$), 151.6 ($CH_2N^1N^2$=*C*H$N^4$), 169.6 (*C*=O); HRMS (ESI$^+$, *m*/*z*): $[M + H]^+_{calcd}$ = 170.0924, $[M + H]^+_{found}$ = 170.1215; anal. calcd. (%) for $C_7H_{11}N_3O_2$: C, 49.70 H, 6.55 N, 24.84; found: C, 49.67 H, 6.55 N, 24.86; UV–vis: λ_{max} = 201 nm; GC: t_R = 5.258; HPLC [hexane–iPrOH (9:1); f = 0.8]: t_R = 19.231, 24.214.

General procedure for the lipase-catalyzed kinetic resolution of racemic alcohols (±)-3a and (±)-3b

In a typical experiment, the reaction mixture was composed of 0.3 g of the appropriate racemic alcohol (±)-**3a** or (±)-**3b**, 5-fold molar excess of vinyl acetate, 3 ml of organic solvent, and 50–100 mg of lipase (depending of the enzyme used – see Table 1 and 2). The mixture was agitated (250 rpm) at room temperature. Conversion rate was measured by GC. The reaction was stopped by filtering off the enzyme. To the remaining lipase the same organic solvent (3 ml) as used in the reaction was added and suspension was stirred for a minimum of 10 min. Then, the enzyme was filtered off via a funnel stocked with cotton and washed with methanol (3 ml) in order to elute residuals of the products. The crude product was evaporated to dryness and purified by chromatography using a mixture of $CHCl_3$–MeOH (9:1) as eluent (details are given in Tables 1 and 2; physical, spectroscopic and analytical

data are identical as for the racemic standard compounds). The specific rotations were measured in $CHCl_3$ solution for the enantioenriched alcohols and esters, and are as follows:

(*S*)-(+)-**5a**: $[\alpha]_D^{29}$ = +35.2 (*c* 1.0, $CHCl_3$) for ee = 98%

(*R*)-(+)-**6a**: $[\alpha]_D^{29}$ = +9.2 (*c* 1.0, $CHCl_3$) for ee > 99%

(*S*)-(+)-**5b**: $[\alpha]_D^{29}$ = +44 (*c* 1.0, $CHCl_3$) for ee = 98%

(*R*)-(+)-**6b**: $[\alpha]_D^{29}$ = +5.2 (*c* 1.0, $CHCl_3$) for ee = 90%

General procedure for the determination of the absolute configuration of (+)-5a (+)-5b

Esterification of (*S*)-(+)-1-(1*H*-imidazol-1-yl)propan-2-ol (+)-5a with (*R*)- or (*S*)-α-methoxy-α-phenylacetic acid. A catalytic amount of DMAP (5 mg) was added to a solution of (*S*)-(+)-1-(1*H*-imidazol-1-yl)propan-2-ol (+)-**5a** (50 mg; 0.4 mmol), (*R*)- or (*S*)-α-methoxy-α-phenylacetic acid (66 mg; 0.4 mmol), as appropriate, and DCC (98 mg; 0.48 mmol) in dry CH_2Cl_2 (2.5 ml). After 24 h of stirring at rt, precipitated dicyclohexylurea was removed by filtration and then the urea cake was rinsed with toluene (3 × 5 ml). The combined toluene solution was evaporated in vacuum. Crude product as a yellow oil was purified by preparative chromatographic plate with a mixture of hexane–ethyl acetate (1:1). Appropriately, the separated fraction was removed from the plate together with silica gel, placed in a round-bottomed flask, and stirred with 10 ml of $PhCH_3$–AcOEt (8:2) for over 1 h. Finally, silica gel was filtered off, the fraction was rinsed with $PhCH_3$ (2 × 2.5 ml), and the solvents were removed under reduced pressure to afford the pure product.

(*S*)-((*S*)-1-(1*H*-Imidazol-1-yl)propan-2-yl) 2-methoxy-2-phenylacetate (9a): Yellow oil; yield 68%; R_f = 0.09 [hexane–ethyl acetate (1:1)]; ^{1}H NMR (CDCl$_3$, 400 MHz) δ 1.19 (d, *J* = 6.32 Hz, 3H, CH_3CH(OH)CH$_2$), 3.36 (s, 3H, OCH_3), 3.85–3.97 (m, 2H, MPAOCHCH_2N^1), 4.72 (s, 1H, C*H*OCH$_3$), 5.14–5.24 (m, 1H, MPAOC*H*CH$_2$N^1), 6.45 (s, 1H, CH$_2$N^{1}C*H*=N^3), 6.88 (s, 1H, CH$_2$N^{1}CH=C*H*N^3), 7.07 (s, 1H, CH$_2$N^{1}C*H*=CHN^3), 7.36–7.44 (m, 5H, Ph); ^{13}C NMR (CDCl$_3$, 100 MHz) δ 16.9 (*C*H$_3$CH(OMPA)CH$_2$), 50.7 (MPAOCH*C*H$_2$N^1), 57.2 (O*C*H$_3$), 70.0 (MPAO*C*HCH$_2$N^1), 82.4 (*C*HOCH$_3$), 119.6 (CH$_2$N^1*C*H=CHN^3), 127.3 (2C, Ph), 128.8 (CH$_2$N^{1}CH=*C*HN^3), 129.0 (2C, Ph), 129.2 (Ph), 135.8 (Ph), 137.4 (CH$_2$N^1*C*H=N^3), 169.8 (*C*=O).

((*R*)-((*S*)-1-(1*H*-Imidazol-1-yl)propan-2-yl) 2-methoxy-2-phenylacetate (10a): Yellow oil; yield 74%; R_f = 0.09 [hexane–ethyl acetate (1:1)]; ^{1}H NMR (CDCl$_3$, 400 MHz) δ 1.10 (d, *J* = 6.32 Hz, 3H, CH_3CH(OMPA)CH$_2$), 3.35 (s, 3H, OCH_3), 3.95–4.09 (m, 2H, MPAOCHCH_2N^1), 4.71 (s, 1H, C*H*OCH$_3$), 5.10–5.22 (m, 1 H, MPAOC*H*CH$_2$N^1), 6.82 (s, 1H, CH$_2$N^{1}CH=C*H*N^3), 7.01 (s, 1H, CH$_2$N^{1}C*H*=CHN^3), 7.32–7.38 (m, 5H, Ph), 7.39 (s, 1H, CH$_2$N^{1}C*H*=N^3); ^{13}C NMR (CDCl$_3$, 100 MHz) δ 16.8 (*C*H$_3$CH(OMPA)CH$_2$), 50.8 (MPAOCH*C*H$_2$N^1), 57.4 (O*C*H$_3$), 70.1 (MPAO*C*HCH$_2$N^1), 82.5 (*C*HOCH$_3$), 119.5 (CH$_2$N^1*C*H=CHN^3), 126.9 (2C, Ph), 128.7 (2C, Ph), 128.9 (Ph), 129.1 (CH$_2$N^{1}CH=*C*HN^3), 135.7 (Ph), 137.6 (CH$_2$N^1*C*H=N^3), 169.9 (*C*=O).

Esterification of (*S*)-(+)-1-(1*H*-1,2,4-triazol-1-yl)propan-2-ol (+)-5b with (*R*)- or (*S*)-α-methoxy-α-phenylacetic acid. A catalytic amount of DMAP (5 mg) was added to a solution of (*S*)-(+)-1-(1*H*-1,2,4-triazol-1-yl)propan-2-ol (+)-**5b** (50 mg; 0.39 mmol), (*R*)- or (*S*)-α-methoxy-α-phenylacetic acid (66 mg; 0.39 mmol), as appropriate, and

DCC (97.4 mg; 0.47 mmol) in dry methylene chloride (2.5 ml). After 24 h of stirring at room temp., precipitated dicyclohexylurea was removed by filtration and then the urea cake was rinsed with toluene (3 × 5 ml). The combined organic solutions were washed with cold 1 M HCl (2 × 1 ml), saturated $NaHCO_3$ (2 × 1 ml), and saturated NaCl (1 × 1 ml). Then the organic layer was dried over $MgSO_4$ and filtered, and the solvent was evaporated under reduced pressure. The crude product was purified by preparative plate chromatography using hexane–ethyl acetate (1:1) mixture.

(*S*)-((*S*)-1-(1*H*-1,2,4-Triazol-1-yl)propan-2-yl) 2-methoxy-2-phenylacetate (9b): Yellow oil; yield 63%; R_f = 0.09 [hexane–ethyl acetate (1:1)]; ^{1}H NMR ($CDCl_3$, 400 MHz) δ 1.25 (d, *J* = 6.55 Hz, 3H, CH_3CH(OMPA)CH_2), 3.34 (s, 3H, OCH_3), 4.16 (d, *J* = 4.52 Hz, 2H, MPAOCHCH_2N^1), 4.69 (s, 1H, CHOCH_3), 5.23–5.36 (m, 1H, MPAOCHCH_2N^1), 7.28 (s, 1H, $CH_2N^1CH{=}N^4$), 7.32–7.47 (m, 5H, Ph), 7.79 (s, 1H, $CH_2N^1N^2{=}CHN^4$); ^{13}C NMR ($CDCl_3$, 100 MHz) δ 17.1 (CH_3CH(OMPA)CH_2), 53.1 (MPAOCHCH_2N^1), 57.2 (OCH_3), 69.3 (MPAOCHCH_2N^1), 82.3 (CHOCH_3), 126.9 ($CH_2N^1CH{=}N^4$), 127.2 (Ph), 128.9 (2C, Ph), 129.2 (2C, Ph), 135.8 (Ph), 151.6 ($CH_2N^1N^2{=}CHN^4$), 169.6 (*C*=O).

(*R*)-((*S*)-1-(1*H*-1,2,4-Triazol-1-yl)propan-2-yl) 2-methoxy-2-phenylacetate (10b): Yellow oil; yield 59%; R_f = 0.09 [hexane–ethyl acetate (1:1)]; ^{1}H NMR ($CDCl_3$, 400 MHz) δ 1.17 (d, *J* = 6.32 Hz, 3H, CH_3CH(OMPA)CH_2), 3.34 (s, 3H, OCH_3), 4.18–4.34 (m, 2H, MPAOCHCH_2N^1), 4.69 (s, 1H, CHOCH_3), 5.26–5.30 (m, 1H, MPAOCHCH_2N^1), 7.28–7.41 (m, 5H, Ph), 7.89 (d, *J* = 5.87 Hz, 2H, $CH_2N^1N^2{=}CHN^4$ and $CH_2N^1CH{=}N^4$); ^{13}C NMR ($CDCl_3$, 100 MHz) δ 16.9 (CH_3CH(OMPA)CH_2), 53.2 (MPAOCHCH_2N^1), 57.3 (OCH_3), 69.4 (MPAOCHCH_2N^1), 82.4 (CHOCH_3), 126.8 (Ph),

128.7 (2C, Ph), 128.8 (2C, Ph), 135.7 (Ph), 143.8 (CH_2N^1*C*H=N^4), 151.9 ($CH_2N^1N^2$=*C*HN^4), 169.7 (*C*=O).

General methods for the preparation of chiral ionic liquids

Synthesis and characterization of optically active imidazolium salts [(+)-7a–h]. Optically active (*S*)-(+)-1-(1*H*-imidazol-1-yl)propan-2-ol (+)-**5a** (0.5 g; 3.96 mmol) was dissolved in dry CH_3CN (5 ml), and subsequently a 3-fold molar excess of the appropriate freshly distilled alkyl bromide or iodide was added. The reaction mixture was stirred under the conditions given in Table 3. The reaction progress was monitored by TLC with a chloroform–methanol (8:2) mixture as the eluent. The reaction mixture was cooled to room temperature, and the separated salt was washed with Et_2O (3 × 5 ml) and *n*-hexane (5 ml) to afford a dark oil, which was sequentially purified from tars with a column filled with activated carbon. The resulting product was a colored oil.

(*S*)-(+)-1-(2-Hydroxypropyl)-3-propyl-1*H*-imidazol-3-ium bromide (+)-7a: White oil; yield 92%; R_f = 0.26 [$CHCl_3$–MeOH (8:2)]; ^{1}H NMR ($CDCl_3$, 400 MHz) δ 0.97 (t, *J* = 7.34 Hz, 3H, CH_2CH_3), 1.25 (d, *J* = 6.32 Hz, 3H, $CH_3CH(OH)CH_2$), 1.89–2.01 (m, 2H, CH_2CH_3), 4.13–4.26 (m, 2H, $^+N^3CH_2CH_2CH_3$), 4.28–4.36 (m, 2H, $HOCHCH_2N^1$), 4.50 (m, 1H, HOC*H*CH_2N^1), 7.40 (t, *J* = 1.58 Hz, 1H, CH_2N^1CH=C*H*N^3), 7.64 (t, *J* = 1.47 Hz, 1H, CH_2N^1C*H*=CHN^3), 9.74 (s, 1H, CH_2N^1C*H*=N^3); ^{13}C NMR ($CDCl_3$, 100 MHz) δ 10.7 (CH_2CH_3), 20.3 (CH_2CH_3), 23.4 ($CH_3CH(OH)CH_2$), 51.5 ($^+N^3CH_2CH_2CH_3$), 56.1 (HOCH*C*H_2N^1), 65.5 (HO*C*HCH_2N^1), 121.2 (CH_2N^1CH=*C*HN^3), 123.6 (CH_2N^1*C*H=CHN^3), 136.7 (CH_2N^1*C*H=N^3); HRMS (ESI$^+$,

m/z): $[M^+]_{calcd}$ = 169.1329, $[M^+]_{found}$ = 169.4186; anal. calcd. (%) for $C_9H_{17}BrN_2O$: C, 43.39 H, 6.88 N, 11.24; found: C, 43.34 H, 6.89 N, 11.21; IR (neat, ν cm^{-1}): 3233, 3070, 2968, 1563, 1451, 1375, 1274, 1163, 1135, 1072, 937, 846, 782, 753, 627, 472; $[\alpha]_D^{29}$ = +23.6 (*c* 1.0, $CHCl_3$) for ee = 98%.

(*S*)-(+)-1-(2-Hydroxypropyl)-3-(prop-2-en-1-yl)-1*H*-imidazol-3-ium bromide (+)-7b: Dark brown oil; yield 93%; R_f = 0.31 [$CHCl_3$–MeOH (8:2)]; ^{1}H NMR ($CDCl_3$, 400 MHz) δ 1.24 (d, *J* = 6.32 Hz, 3H, $CH_3CH(OH)CH_2$), 4.13–4.22 (m, 1H, $HOCHCH_2N^1$), 4.23–4.31 (m, 1H, $HOCHCH_2N^1$), 4.47 (dd, *J* = 13.55, 2.71 Hz, 1H, $HOCHCH_2N^1$), 4.92 (d, *J* = 6.32 Hz, 2H, $^+N^3CH_2CH_a{=}CH_aH_b$), 5.39–5.50 (m, 2H, $^+N^3CH_2CH_a{=}CH_aH_b$), 5.95–6.09 (m, 1H, $^+N^3CH_2CH_a{=}CH_aH_b$), 7.37 (t, *J* = 1.81 Hz, 1H, $CH_2N^1CH{=}CHN^3$), 7.66 (t, *J* = 1.81 Hz, 1H, $CH_2N^1CH{=}CHN^3$), 9.65–9.70 (m, 1H, $CH_2N^1CH{=}N^3$); ^{13}C NMR ($CDCl_3$, 100 MHz) δ 20.3 ($CH_3CH(OH)CH_2$), 52.1 ($^+N^3CH_2CH{=}CH_2$), 56.3 ($HOCHCH_2N^1$), 65.4 ($HOCHCH_2N^1$), 121.2 ($CH_2N^1CH{=}CHN^3$), 122.6 ($^+N^3CH_2CH{=}CH_2$), 123.7 ($CH_2N^1CH{=}CHN^3$), 129.7 ($^+N^3CH_2CH{=}CH_2$), 136.6 ($CH_2N^1CH{=}N^3$); HRMS (ESI^+, *m/z*): $[M^+]_{calcd}$ = 167.1173, $[M^+]_{found}$ = 167.0548; anal. calcd. (%) for $C_9H_{15}BrN_2O$: C, 43.74 H, 6.12 N, 11.34; found: C, 43.69 H, 6.11 N, 11.37; IR (neat, ν cm^{-1}): 3297, 3072, 2970, 1560, 1444, 1423, 1161, 1135, 1078, 994, 938, 840, 759, 623, 579, 471; $[\alpha]_D^{29}$ = +25.3 (*c* 1.0, $CHCl_3$) for ee = 98%.

(*S*)-(+)-3-Butyl-1-(2-hydroxypropyl)-1*H*-imidazol-3-ium bromide (+)-7c: Yellowish oil; yield 94%; R_f = 0.37 [$CHCl_3$–MeOH (8:2)]; ^{1}H NMR ($CDCl_3$, 400 MHz) δ 0.76 (t, *J* = 7.34 Hz, 3H, CH_2CH_3), 1.07 (d, *J* = 6.32 Hz, 3H, $CH_3CH(OH)CH_2$), 1.13–1.25 (m, 2H, CH_2CH_3), 1.67–1.79 (m, 2H, $CH_2CH_2CH_2CH_3$), 3.96–4.05 (m, 1H, $HOCHCH_2N^1$), 4.07–4.21 (m, 2H, $^+N^3CH_2CH_2CH_2CH_3$), 4.29 (s, 1H, $HOCHCH_2N^1$), 4.37 (dd, *J* =

13.55, 2.94 Hz, 1H, HOCHCH_2N[1]), 7.37 (t, *J* = 1.81 Hz, 1H, CH_2N^1C*H*=CHN^3), 7.60 (t, *J* = 1.69 Hz, 1H, CH_2N^1CH=C*H*N^3), 9.50 (s, 1H, CH_2N^1C*H*=N^3); ^{13}C NMR ($CDCl_3$, 100 MHz) δ 13.0 (CH_2*C*H_3), 19.0 (*C*H_3CH(OH)CH_2), 19.8 (*C*H_2CH_3), 31.5 (CH_2*C*$H_2CH_2CH_3$), 49.3 ($^+N^3$*C*$H_2CH_2CH_2CH_3$), 55.5 (HOCH*C*H_2N^1), 65.1 (HO*C*HCH_2N^1), 121.1 (CH_2N^1CH=*C*HN^3), 123.4 (CH_2N^1*C*H=CHN^3), 135.9 (CH_2N^1*C*H=N^3); HRMS (ESI$^+$, *m/z*): $[M^+]_{calcd}$ = 183.1486, $[M^+]_{found}$ = 183.5657; anal. calcd. (%) for $C_{10}H_{19}BrN_2O$: C, 45.64 H, 7.28 N, 10.64; found: C, 45.60 H, 7.30 N, 10.61; IR (neat, ν cm^{-1}): 3294, 3068, 2962, 1562, 1460, 1372, 1164, 1137, 1078, 948, 841, 753, 623, 470; $[\alpha]_D^{29}$ = +17.2 (*c* 1.0, $CHCl_3$) for ee = 98%.

(*S*)-(+)-1-(2-Hydroxypropyl)-3-pentyl-1*H*-imidazol-3-ium bromide (+)-7d: Red oil; yield 82%; R_f = 0.43 [$CHCl_3$–MeOH (8:2)]; ^{1}H NMR ($CDCl_3$, 400 MHz) δ 0.72 (t, *J* = 6.99 Hz, 3H, CH_2C*H*$_3$), 1.10 (d, *J* = 6.32 Hz, 3H, C*H*$_3$CH(OH)CH_2), 1.13–1.21 (m, 4H, $^+N^3CH_2CH_2$C*H*$_2$C*H*$_2CH_3$), 1.74–1.78 (m, 2H, $^+N^3CH_2$C*H*$_2CH_2CH_2CH_3$), 3.99–4.23 (m, 4H, $^+N^3$C*H*$_2CH_2CH_2CH_2CH_3$, HOC*H*CH_2N^1 and HOCHC*H*$_2N^1$), 4.39 (dd, *J* = 13.66, 2.82 Hz, 1H, HOCHC*H*$_2N^1$), 4.77 (br. s., 1H, O*H*), 7.36 (d, *J* = 1.58 Hz, 1H, CH_2N^1C*H*=CHN^3), 7.63 (d, *J* = 1.35 Hz, 1H, CH_2N^1CH=C*H*N^3), 9.52 (s, 1H, CH_2N^1C*H*=N^3); ^{13}C NMR ($CDCl_3$, 100 MHz) δ 13.43 (CH_2*C*H_3), 19.9 (*C*H_3CH(OH)CH_2), 21.6 (*C*H_2CH_3), 27.8 ($^+N^3CH_2$*C*$H_2CH_2CH_2CH_3$), 29.4 ($^+N^3CH_2CH_2$*C*$H_2CH_2CH_3$), 49.6 ($^+N^3$*C*$H_2CH_2CH_2CH_2CH_3$), 55.6 (HOCH*C*H_2N^1), 65.2 (HO*C*HCH_2N^1), 121.0 (CH_2N^1CH=*C*HN^3), 123.5 (CH_2N^1*C*H=CHN^3), 135.9 (CH_2N^1*C*H=N^3); HRMS (ESI$^+$, *m/z*): $[M^+]_{calcd}$ = 197.1643, $[M^+]_{found}$ = 197.0844; anal. calcd. (%) for $C_{11}H_{21}BrN_2O$: C, 47.66 H, 7.64 N, 10.11; found: C, 47.61 H, 7.66 N, 10.15; IR (neat, ν cm^{-1}): 3298, 3071, 2959, 2931, 1562, 1457, 1373, 1163, 1137, 1078, 937, 841, 747, 623, 473; $[\alpha]_D^{29}$ = +15.4 (*c* 1.0, $CHCl_3$) for ee = 98%.

(*S*)-(+)-3-Heptyl-1-(2-hydroxypropyl)-1*H*-imidazol-3-ium iodide (+)-7e: Cherry-red oil; yield 92%; R_f = 0.52 [$CHCl_3$–MeOH (8:2)]; ^1H NMR ($CDCl_3$, 400 MHz) δ 0.64 (t, *J* = 6.44 Hz, 3H, CH_2CH_3), 0.94–1.22 (m, 11H, $^+N^3CH_2CH_2CH_2CH_2CH_2CH_2CH_3$ and $CH_3CH(OH)CH_2$), 1.70–1.74 (m, 2H, $^+N^3CH_2CH_2CH_2CH_2CH_2CH_2CH_3$), 3.88 (br. s., 1H, O*H*), 3.98–4.18 (m, 4H, HOC*H*CH_2N^1, one of HOCHCH_2N^1 and $^+N^3CH_2CH_2CH_2CH_2CH_2CH_2CH_3$), 4.36 (d, *J* = 11.29 Hz, 1H, one of HOCHCH_2N^1), 7.32 (s, 1H, $CH_2N^1CH{=}CHN^3$), 7.55 (s, 1H, $CH_2N^1CH{=}CHN^3$), 9.26 (s, 1H, $CH_2N^1CH{=}N^3$); ^{13}C NMR ($CDCl_3$, 100 MHz) δ 13.6 (CH_2CH_3), 19.9 ($CH_3CH(OH)CH_2$), 22.0 (CH_2CH_3), 25.7 ($^+N^3CH_2CH_2CH_2CH_2CH_2CH_2CH_3$), 28.1 ($^+N^3CH_2CH_2CH_2CH_2CH_2CH_2CH_3$), 29.6 ($^+N^3CH_2CH_2CH_2CH_2CH_2CH_2CH_3$), 31.0 ($^+N^3CH_2CH_2CH_2CH_2CH_2CH_2CH_3$), 49.8 ($^+N^3CH_2CH_2CH_2CH_2CH_2CH_2CH_3$), 55.7 (HOCH$CH_2N^1$), 65.1 (HO*C*H$CH_2N^1$), 121.2 ($CH_2N^1CH{=}CHN^3$), 123.4 ($CH_2N^1CH{=}CHN^3$), 135.4 ($CH_2N^1CH{=}N^3$); HRMS (ESI$^+$, *m*/*z*): $[M^+]_{calcd}$ = 225.1955, $[M^+]_{found}$ = 225.0930; anal. calcd. (%) for $C_{13}H_{25}IN_2O$: C, 44.33 H, 7.15 N, 7.95; found: C, 44.37 H, 7.14 N, 7.99; IR (neat, ν cm^{-1}): 3337, 3080, 2926, 2856, 1561, 1457, 1374, 1162, 1136, 1092, 1076, 936, 838, 745, 635, 473; $[\alpha]_D^{29}$ = +16.3 (*c* 1.0, $CHCl_3$) for ee = 98%.

(*S*)-(+)-3-Decyl-1-(2-hydroxypropyl)-1*H*-imidazol-3-ium iodide (+)-7f: Orange oil; yield 92%; R_f = 0.55 [$CHCl_3$–MeOH (8:2)]; ^1H NMR ($CDCl_3$, 400 MHz) δ 0.68 (t, *J* = 6.94 Hz, 3H, CH_2CH_3), 0.95–1.27 (m, 17H, $^+N^3CH_2CH_2CH_2CH_2CH_2CH_2CH_2CH_2CH_2CH_3$ and $CH_3CH(OH)CH_2$), 1.71–1.76 (m, 2H, $^+N^3CH_2CH_2$), 3.82–4.25 (m, 4H, HOC*H*CH_2N^1, one of HOCHCH_2N^1 and $^+N^3CH_2$), 4.32–4.45 (m, 1H, one of HOCHCH_2N^1), 7.32 (t, *J* = 1.69 Hz, 1H, $CH_2N^1CH{=}CHN^3$),

7.58 (t, *J* = 1.69 Hz, 1H, $CH_2N^1CH{=}CHN^3$), 9.29 (s, 1H, $CH_2N^1CH{=}N^3$); ^{13}C NMR ($CDCl_3$, 100 MHz) δ 13.35 (CH_2CH_3), 19.7 ($CH_3CH(OH)CH_2$), 21.7 (CH_2), 25.4 (CH_2), 27.4 (CH_2), 27.9 (CH_2), 29.3 (CH_2), 29.7 (CH_2), 30.8 (CH_2), 32.8 (CH_2), 49.5 ($^+N^3CH_2$), 55.4 ($HOCHCH_2N^1$), 64.9 ($HOCHCH_2N^1$), 121.0 ($CH_2N^1CH{=}CHN^3$), 123.2 ($CH_2N^1CH{=}CHN^3$), 135.2 ($CH_2N^1CH{=}N^3$); HRMS (ESI$^+$, *m*/*z*): $[M^+]_{calcd}$ = 267.2425, $[M^+]_{found}$ = 267.1200; anal. calcd. (%) for $C_{16}H_{31}IN_2O$: C, 48.73 H, 7.92 N, 7.10; found: C, 48.76 H, 7.91 N, 7.12; IR (neat, v cm^{-1}): 3333, 3081, 2922, 2853, 1561, 1458, 1376, 1163, 1137, 1076, 936, 745, 635, 472; $[\alpha]_D^{29}$ = +15.8 (*c* 1.0, $CHCl_3$) for ee = 98%.

(*S*)-(+)-3-Dodecyl-1-(2-hydroxypropyl)-1*H*-imidazol-3-ium iodide (+)-7g: Cherry-red oil; yield 86%; R_f = 0.51 [$CHCl_3$: MeOH (8:2)]; ^1H NMR ($CDCl_3$, 400 MHz) δ 0.83 (t, *J* = 6.59 Hz, 3H, CH_2CH_3), 1.15–1.25 (m, 18H, $^+N^3CH_2CH_2CH_2CH_2CH_2CH_2CH_2CH_2CH_2CH_2CH_2CH_3$), 1.28 (d, *J* = 5.87 Hz, 3H, $CH_3CH(OH)CH_2$), 1.81–2.02 (m, 2H, $^+N^3CH_2CH_2$), 3.78 (d, *J* = 4.97 Hz, 1H, C*H*), 4.12–4.35 (m, 3H, CH_2), 4.46 (d, *J* = 11.07 Hz, 1H, C*H*), 7.35 (t, *J* = 1.81 Hz, 1H, $CH_2N^1CH{=}CHN^3$), 7.59 (t, *J* = 1.69 Hz, 1H, $CH_2N^1CH{=}CHN^3$), 9.21 (s, 1H, $CH_2N^1CH{=}N^3$); ^{13}C NMR ($CDCl_3$, 100 Hz) δ 14.0 (CH_2CH_3), 20.4 ($CH_3CH(OH)CH_2$), 22.5 (CH_2CH_3), 26.2 (CH_2), 28.3 (CH_2), 28.8 (CH_2), 29.1 (CH_2), 29.4 (2C, CH_2), 29.9 (CH_2), 30.3 (CH_2), 31.7 (CH_2), 50.5 ($^+N^3CH_2$), 56.4 ($HOCHCH_2N^1$), 65.5 ($HOCHCH_2N^1$), 121.6 ($CH_2N^1CH{=}CHN^3$), 123.8 ($CH_2N^1CH{=}CHN^3$), 135.8 ($CH_2N^1CH{=}N^3$); HRMS (ESI$^+$, *m*/*z*): $[M^+]_{calcd}$ = 295.2738, $[M^+]_{found}$ = 295.8576; anal. calcd. (%) for $C_{18}H_{35}IN_2O$: C, 51.18 H, 8.35 N, 6.63; found: C, 51.22 H, 8.32 N, 6.67; IR (neat, v cm^{-1}): 3332, 2921, 2852, 1560, 1466, 1376, 1162, 1091, 1075, 936, 832, 745, 622, 472; $[\alpha]_D^{29}$ = +14.2 (*c* 1.0, $CHCl_3$) for ee = 98%.

(S)-(+)-3-Hexadecyl-1-(2-hydroxypropyl)-1H-imidazol-3-ium iodide (+)-7h: Colorless oil; yield 81%; R_f = 0.58 [$CHCl_3$: MeOH (8:2)]; ^1H NMR ($CDCl_3$, 400 MHz) δ 0.82 (t, J = 6.78 Hz, 3H, CH_2CH_3), 1.17–1.31 (m, 29H, $^+N^3CH_2CH_2(CH_2)_{13}CH_3$ and $CH_3CH(OH)CH_2$), 1.80–1.94 (m, 2H, $^+N^3CH_2CH_2$), 4.15–4.29 (m, 4H, $HOCHCH_2N^1$ and $^+N^3CH_2$), 4.43 (s, 1H, $HOCHCH_2N^1$), 7.33 (t, J = 1.69 Hz, 1H, $CH_2N^1CH{=}CHN^3$), 7.59 (t, J = 1.58 Hz, 1H, $CH_2N^1CH{=}CHN^3$), 9.39 (s, 1H, $CH_2N^1CH{=}N^3$); ^{13}C NMR ($CDCl_3$, 100 Hz) δ 14.0 (CH_2CH_3), 20.1 ($CH_3CH(OH)CH_2$), 22.5 (CH_2CH_3), 26.1 (CH_2), 28.8 (CH_2), 29.2 (CH_2), 29.2 (CH_2), 29.4 (CH_2), 29.5 (CH_2), 29.5 (CH_2), 29.5 (CH_2), 29.5 (3C, CH_2), 29.9 (CH_2), 31.7 (CH_2), 50.2 ($^+N^3CH_2$), 56.2 ($HOCHCH_2N^1$), 65.4 ($HOCHCH_2N^1$), 121.3 ($CH_2N^1CH{=}CHN^3$), 123.6 ($CH_2N^1CH{=}CHN^3$), 136.0 ($CH_2N^1CH{=}N^3$); HRMS (ESI$^+$, m/z): $[M^+]_{calcd}$ = 351.3364, $[M^+]_{found}$ = 351.2940; anal. calcd. (%) for $C_{22}H_{43}IN_2O$: C, 55.22 H, 9.06 N, 5.85; found: C, 55.17 H, 9.08 N, 5.81; IR (neat, ν cm^{-1}): 3328, 2914, 2850, 1561, 1473, 1374, 1164, 1136, 1021, 936, 839, 749, 715, 626, 472; $[\alpha]_D^{29}$ = +11.4 (c 1.0, $CHCl_3$) for ee = 98%.

Synthesis and characterization of optically active triazolium salts [(+)-8a–f]. Optically active (S)-(+)-1-(1H-1,2,4-triazol-1-yl)propan-2-ol (+)-**5b** (0.5 g; 3.93 mmol) was dissolved in dry CH_3CN (5 ml), and a 3-fold molar excess of the appropriate freshly distilled alkyl bromide or iodide was added. The reaction mixture was stirred under the conditions given in Table 4. The reaction progress was monitored by TLC using a solution of chloroform–methanol (8:2) as the eluent. After stirring had been stopped and the mixture cooled to 0–5 °C, the separated salt was filtered off, and washed with Et_2O (3 × 5 ml) and toluene (5 ml). Next, the solvents were removed under reduced pressure to yield the product as a viscous liquid (gum) or sticky oil.

(*S*)-(+)-1-(2-Hydroxypropyl)-4-propyl-1*H*-1,2,4-triazol-4-ium bromide (+)-8a: White gum; yield 98%; R_f = 0.31 [$CHCl_3$–MeOH (8:2)]; ^{1}H NMR ($CDCl_3$, 400 MHz) δ 0.94 (t, *J* = 7.38 Hz, 3H, CH_2CH_3), 1.23 (d, *J* = 6.43 Hz, 3H, $CH_3CH(OH)CH_2$), 1.97 (m, 2H, CH_2CH_3), 4.06–4.25 (m, 1H, $HOCHCH_2N^1$), 4.27–4.53 (m, 4H, $HOCHCH_2N^1$ and $^+N^4CH_2$), 8.99 (s, 1H, $CH_2N^1N^2$=CHN^4), 10.62 (s, 1H, CH_2N^1CH=N^4); ^{13}C NMR ($CDCl_3$, 100 MHz) δ 10.6 (CH_2CH_3), 20.0 (CH_2CH_3), 23.0 ($CH_3CH(OH)CH_2$), 49.9 ($^+N^4CH_2$), 59.3 ($HOCHCH_2N^1$), 64.6 ($HOCHCH_2N^1$), 142.8 ($CH_2N^1N^2$=CHN^4), 143.7 (CH_2N^1CH=N^4); HRMS (ESI$^+$, *m*/*z*): $[M^+]_{calcd}$ = 170.1282, $[M^+]_{found}$ = 170.1560; anal. calcd. (%) for $C_8H_{16}BrN_3O$: C, 38.41 H, 6.45 N, 16.80; found: C, 38.36 H, 6.46 N, 16.85; IR (neat, ν cm^{-1}): 3308, 3038, 2971, 1577, 1446, 1374, 1272, 1159, 1137, 1067, 999, 936, 909, 843, 808, 626, 583, 478; $[\alpha]_D^{29}$ = +18.8 (*c* 1.0, $CHCl_3$) for ee = 98%.

(*S*)-(+)-1-(2-Hydroxypropyl)-4-(prop-2-en-1-yl)-1*H*-1,2,4-triazol-4-ium bromide (+)-8b: Brown gum; yield 94%; R_f = 0.18 [$CHCl_3$–MeOH (8:2)]; ^{1}H NMR ($CDCl_3$, 400 MHz) δ 1.23 (d, *J* = 6.43 Hz, 3H, $CH_3CH(OH)CH_2$), 4.12–4.23 (m, 1H, $HOCHCH_2N^1$), 4.33–4.48 (m, 2H, $HOCHCH_2N^1$), 4.82 (br. s., 1H, O*H*), 5.10 (d, *J* = 6.67 Hz, 2H, $^+N^4CH_2$), 5.44 (dd, *J* = 10.12, 0.83 Hz, 1H, $^+N^4CH_2CH_a$=CH_aH_b), 5.54 (dd, *J* = 16.90, 0.71 Hz, 1H, $^+N^4CH_2CH_a$=CH_aH_b), 6.10 (ddt, *J* = 16.96, 10.18, 6.55, 6.55 Hz, 1H, $^+N^4CH_2CH_a$=CH_aH_b), 8.89 (s, 1H, $CH_2N^1N^2$=CHN^4), 10.58 (s, 1H, CH_2N^1CH=N^4); ^{13}C NMR ($CDCl_3$, 100 MHz) δ 20.0 ($CH_3CH(OH)CH_2$), 50.6 ($^+N^4CH_2$), 59.4 ($HOCHCH_2N^1$), 64.6 ($HOCHCH_2N^1$), 123.6 ($^+N^4CH_2CH$=CH_2), 128.8 ($^+N^4CH_2CH$=CH_2), 142.8 ($CH_2N^1N^2$=CHN^4), 143.4 (CH_2N^1CH=N^4); HRMS (ESI$^+$, *m*/*z*): $[M^+]_{calcd}$ = 168.1126, $[M^+]_{found}$ = 168.1497; anal. calcd. (%) for $C_8H_{14}BrN_3O$: C,

38.73 H, 5.69 N, 16.94; found: C, 38.75 H, 5.68 N, 16.92; IR (neat, ν cm^{-1}): 3308, 3039, 2979, 1575, 1443, 1385, 1274, 1243, 1151, 1135, 1078, 988, 944, 842, 780, 626, 578, 479; $[\alpha]_D^{29}$ = +15.6 (*c* 1.0, $CHCl_3$) for ee = 98%.

(*S*)-(+)-4-Butyl-1-(2-hydroxypropyl)-1*H*-1,2,4-triazol-4-ium bromide (+)-8c: White gum; yield 90%; R_f = 0.35 [$CHCl_3$–MeOH (8:2)]; ^{1}H NMR ($CDCl_3$, 400 MHz) δ 0.88 (t, *J* = 7.26 Hz, 3H, CH_2CH_3), 1.21 (d, *J* = 6.43 Hz, 3H, $CH_3CH(OH)CH_2$), 1.28–1.39 (m, 2H, CH_2CH_3), 1.84–1.95 (m, 2H, $^+N^4CH_2CH_2CH_2CH_3$), 4.13–4.22 (m, 1H, $HOCHCH_2N^1$), 4.33–4.49 (m, 4H, $HOCHCH_2N^1$ and $^+N^4CH_2$), 4.67 (br. s., 1H, O*H*), 8.99 (s, 1H, $CH_2N^1N^2$=C*H*N^4), 10.63 (s, 1H, CH_2N^1C*H*=N^4); ^{13}C NMR ($CDCl_3$, 100 MHz) δ 13.2 (CH_2CH_3), 19.3 ($CH_3CH(OH)CH_2$), 20.0 (CH_2CH_3), 31.3 ($^+N^4CH_2CH_2CH_2CH_3$), 48.3 ($^+N^4CH_2$), 59.2 ($HOCHCH_2N^1$), 64.5 ($HOCHCH_2N^1$), 142.8 ($CH_2N^1N^2$=*C*HN^4), 143.6 (CH_2N^1*C*H=N^4); HRMS (ESI^+, *m*/*z*): $[M^+]_{calcd}$ = 184.1439, $[M^+]_{found}$ = 184.1733; anal. calcd. (%) for $C_9H_{18}BrN_3O$: C, 40.92 H, 6.87 N, 15.91; found: C, 40.89 H, 6.87 N, 15.93; IR (neat, ν cm^{-1}): 3320, 2963, 2939, 1778, 1464, 1369, 1308, 1277, 1203, 1165, 1140, 1100, 1068, 1000, 935, 843, 780, 629, 570, 478; $[\alpha]_D^{29}$ = +17.7 (*c* 1.0, $CHCl_3$) for ee = 98%.

(*S*)-(+)-1-(2-Hydroxypropyl)-4-pentyl-1*H*-1,2,4-triazol-4-ium bromide (+)-8d: Brownish oil; yield 78%; R_f = 0.40 [$CHCl_3$–MeOH (8:2)]; ^{1}H NMR ($CDCl_3$, 400 MHz) δ 0.78 (t, *J* = 6.83 Hz, 3H, CH_2CH_3), 1.17 (d, *J* = 6.55 Hz, 3H, $CH_3CH(OH)CH_2$), 1.21–1.26 (m, 4H, $^+N^4CH_2CH_2CH_2CH_2CH_3$), 1.81–1.94 (m, 2H, $^+N^4CH_2CH_2CH_2CH_2CH_3$), 4.13 (m, 1H, $HOCHCH_2N^1$), 4.31–4.39 (m, 4H, $HOCHCH_2N^1$ and $^+N^4CH_2$), 5.03 (br. s., 1H, O*H*), 9.00 (s, 1H, $CH_2N^1N^2$=C*H*N^4), 10.56 (s, 1H, CH_2N^1C*H*=N^4); ^{13}C NMR ($CDCl_3$, 100 MHz) δ 13.5 (CH_2CH_3), 19.8 ($CH_3CH(OH)CH_2$), 21.6 (CH_2CH_3), 27.9

($^{+}N^{4}CH_2CH_2CH_2CH_2CH_3$), 29.0 ($^{+}N^{4}CH_2CH_2\mathit{C}H_2CH_2CH_3$), 48.4 ($^{+}N^{4}\mathit{C}H_2$), 59.1 (HOCH$\mathit{C}H_2N^{1}$), 64.4 (HO$\mathit{C}HCH_2N^{1}$), 142.6 ($CH_2N^{1}N^{2}$=$\mathit{C}HN^{4}$), 143.6 ($CH_2N^{1}\mathit{C}H$=$N^{4}$); HRMS (ESI^{+}, *m*/*z*): $[M^{+}]_{calcd}$ = 198.1595, $[M^{+}]_{found}$ = 198.1956; anal. calcd. (%) for $C_{10}H_{20}BrN_3O$: C, 43.17 H, 7.25 N, 15.10; found: C, 43.19 H, 7.24 N, 15.14; IR (neat, ν cm^{-1}): 3328, 2958, 2931, 1576, 1458, 1376, 1160, 1136, 1075, 996, 937, 907, 846, 761, 624, 475; $[\alpha]_D^{29}$ = +15.4 (*c* 1.0, $CHCl_3$) for ee = 98%.

(*S*)-(+)-4-Heptyl-1-(2-hydroxypropyl)-1*H*-1,2,4-triazol-4-ium iodide (+)-8e: Amber gum; yield 87%; R_f = 0.51 [$CHCl_3$–MeOH (8:2)]; ^{1}H NMR ($CDCl_3$, 400 MHz) δ 0.79 (t, *J* = 6.66 Hz, 3H, $CH_2C\mathit{H}_3$), 1.00–1.48 (m, 11H, $^{+}N^{4}CH_2CH_2C\mathit{H}_2C\mathit{H}_2C\mathit{H}_2C\mathit{H}_2CH_3$ and $C\mathit{H}_3CH(OH)CH_2$), 1.77–2.06 (m, 2H, $^{+}N^{4}CH_2C\mathit{H}_2CH_2CH_2CH_2CH_2CH_3$), 4.14 (br. s., 1H, O*H*), 4.27 (m, 1H, HOC*H*CH$_2N^{1}$), 4.33–4.54 (m, 4H, HOCHCH_2N^{1} and $^{+}N^{4}C\mathit{H}_2$), 8.84 (s, 1H, $CH_2N^{1}N^{2}$=C*H*N^{4}), 10.45 (s, 1H, CH_2N^{1}C*H*=N^{4}); ^{13}C NMR ($CDCl_3$, 100 MHz) δ 13.8 ($CH_2\mathit{C}H_3$), 20.0 ($\mathit{C}H_3CH(OH)CH_2$), 22.2 ($\mathit{C}H_2CH_3$), 25.9 ($^{+}N^{4}CH_2\mathit{C}H_2$), 28.3 ($^{+}N^{4}CH_2CH_2\mathit{C}H_2CH_2CH_2CH_2CH_3$), 29.4 ($^{+}N^{4}CH_2CH_2CH_2\mathit{C}H_2CH_2CH_2CH_3$), 31.2 ($^{+}N^{4}CH_2CH_2CH_2CH_2\mathit{C}H_2CH_2CH_3$), 48.8 ($^{+}N^{4}\mathit{C}H_2$), 59.3 (HOCH$\mathit{C}H_2N^{1}$), 64.5 (HO$\mathit{C}HCH_2N^{1}$), 142.4 ($CH_2N^{1}N^{2}$=$\mathit{C}HN^{4}$), 143.4 ($CH_2N^{1}\mathit{C}H$=$N^{4}$); HRMS (ESI^{+}, *m*/*z*): $[M^{+}]_{calcd}$ = 226.1908, $[M^{+}]_{found}$ = 226.2362; anal. calcd. (%) for $C_{12}H_{24}IN_3O$: C, 40.80 H, 6.85 N, 11.90; found: C, 40.83 H, 6.84 N, 11.92; IR (neat, ν cm^{-1}): 3315, 3054, 2956, 2930, 2857, 1574, 1455, 1412, 1372, 1308, 1162, 1135, 1094, 1073, 1001, 935, 891, 846, 767, 721, 619, 526, 477; $[\alpha]_D^{29}$ = +10.2 (*c* 1.0, $CHCl_3$) for ee = 98%.

(*S*)-(+)-4-Decyl-1-(2-hydroxypropyl)-1*H*-1,2,4-triazol-4-ium iodide (+)-8f: Amber gum; yield 92%; R_f = 0.55 [$CHCl_3$–MeOH (8:2)]; ^{1}H NMR ($CDCl_3$, 400 MHz)

δ 0.78 (t, J = 6.78 Hz, 3H, CH_2CH_3), 1.10–1.30 (m, 17H, $^+N^4CH_2CH_2CH_2CH_2CH_2CH_2CH_2CH_2CH_2CH_3$ and $CH_3CH(OH)CH_2$), 1.91–1.95 (m, 2H, $^+N^4CH_2CH_2$), 4.13–4.14 (m, 1H, $HOCHCH_2N^1$), 4.25 (br.s., 1H, O*H*), 4.32–4.51 (m, 4H, $HOCHCH_2N^1$ and $^+N^4CH_2$), 8.83 (s, 1H, $CH_2N^1N^2{=}CHN^4$), 10.42 (s, 1H, $CH_2N^1CH{=}N^4$); ^{13}C NMR ($CDCl_3$, 100 MHz) δ 13.8 (CH_2CH_3), 19.9 ($CH_3CH(OH)CH_2$), 22.3 (CH_2), 26.0 (CH_2), 28.6 (CH_2), 28.9 (CH_2), 29.1 (CH_2), 29.1 (CH_2), 29.4 (CH_2), 31.5 (CH_2), 48.8 ($^+N^4CH_2$), 59.3 ($HOCHCH_2N^1$), 64.4 ($HOCHCH_2N^1$), 142.3 ($CH_2N^1N^2{=}CHN^4$), 143.3 ($CH_2N^1CH{=}N^4$); HRMS (ESI^+, *m*/*z*): $[M^+]_{calcd}$ = 268.2378, $[M^+]_{found}$ = 268.2930; anal. calcd. (%) for $C_{15}H_{30}IN_3O$: C, 45.57 H, 7.65 N, 10.63; found: C, 45.55 H, 7.65 N, 10.66; IR (neat, ν cm^{-1}): 3310, 3056, 2922, 2852, 1574, 1454, 1412, 1364, 1337, 1308, 1290, 1162, 1136, 1095, 1073, 1001, 935, 893, 846, 767, 721, 641, 620, 526, 463; $[\alpha]_D^{29}$ = + 8.5 (*c* 1.0, $CHCl_3$) for ee = 98%.

Microorganisms used

The strains were of the American Type Culture Collection (ATCC): *Escherichia coli* ATCC 8739, *Salmonella typhimurium* ATCC 14028, *Pseudomonas aeruginosa* ATCC 9027, *Bacillus subtilis* ATCC 6633, *Staphylococcus aureus* ATCC 6538, *Candida albicans* ATCC 10231, *Aspergillus brasiliensis* ATCC 16404. The strains received from the IHAR-PIB collection of Plant Breeding and Acclimatization Institute, Młochów: *Colletotrichum coccodis* MC 1, *Fusarium culmorum* MF 18, *Fusarium oxysporu*m MF 5, *Fusarium sambucinum* MF 1, *Phytophthora infestans* MP 324, *Phytophthora infestans* MP 1320.

Antimicrobal activity assays

Qualitative evaluation of the antimicrobial activity was determined by the agar diffusion method according to Rebros et al. [1] with some modifications. Bacterial lawns were grown in liquid TSB (Merck) medium for 24 h. Inocula of approximately 2.5–3.5 × 10^8 CFU/ml (200 μl) were spread on solid TSA (Merck) medium. Subsequently, sterile 8 mm paper discs were placed on the surface of the agar and 20 μl of the tested ILs at the 0.025 M concentration was added. Plates were incubated for 24 h at 30 °C and the diameter of growth-inhibition zones were measured to the nearest millimeter. Each test was performed in duplicate. The ILs used in the experiment were diluted with 0.1–1% EtOH. It was verified in a separate experiment that EtOH at a concentration of 0.1–1% does not inhibit bacterial growth by itself (data not shown).

Quantitative evaluation of antimicrobal activity was determined by tube dilution method [2]. Bacterial and yeast strains were cultured for 24 h on TSB (Merck) medium. The ILs were diluted in TSB medium and subsequently the same amount of inoculum was added to each tube. Positive control was prepared without ILs. Growth (or lack thereof) of the microorganisms was determined visually after incubation for 24 h at 30 °C. The lowest concentration at which there was no visible growth (turbidity) was taken as the minimal inhibitory concentration (MIC).

Antifungal activity was evaluated by measuring inhibition of radial growth on an agar medium in a Petri dish (Liu et al. [3]). The ILs were dissolved in EtOH (96%). Subsequently, the appropriate diluted ILs were added to a PDA (Merck) medium or, in the case of *P. infestans* strains, to a Rye agar [4] medium to give a final

concentration of 1 mM of ILs and below 0.1% of ethanol. The 8 mm disk were cut from an actively growing mycelium of the fungi and placed in the center of the agar plate containing the proper dilution of ILs. The positive control was prepared without ILs. The radial growth of fungal colonies was measured after several days of incubation at 25 °C (in the case of *P. infestans* strains 18 °C). The inhibitory activity of ILs was expressed as a percentage of the positive control growth. Each experiment was carried out twice and the data presented here are the average of two experiments.

1-(1*H*-Imidazol-1-yl)propan-2-ol (±)-3a:

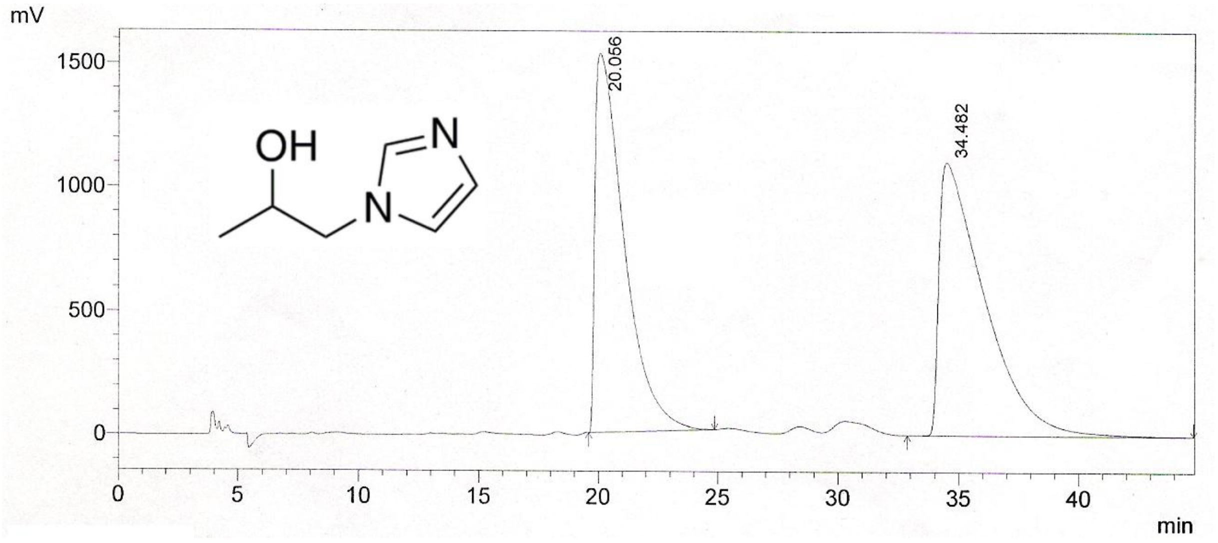

(*S*)-(+)-1-(1*H*-Imidazol-1-yl)propan-2-ol [(*S*)-(+)-5a]

$[\alpha]_D^{29}$ = +35.2 (*c* 1.0, $CHCl_3$) for ee = 98%

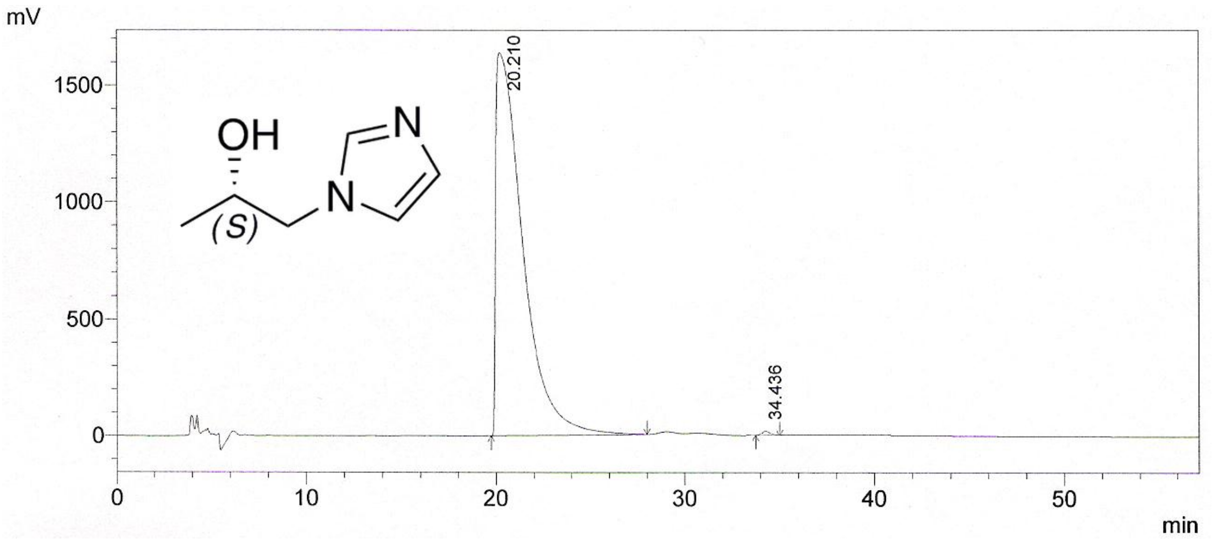

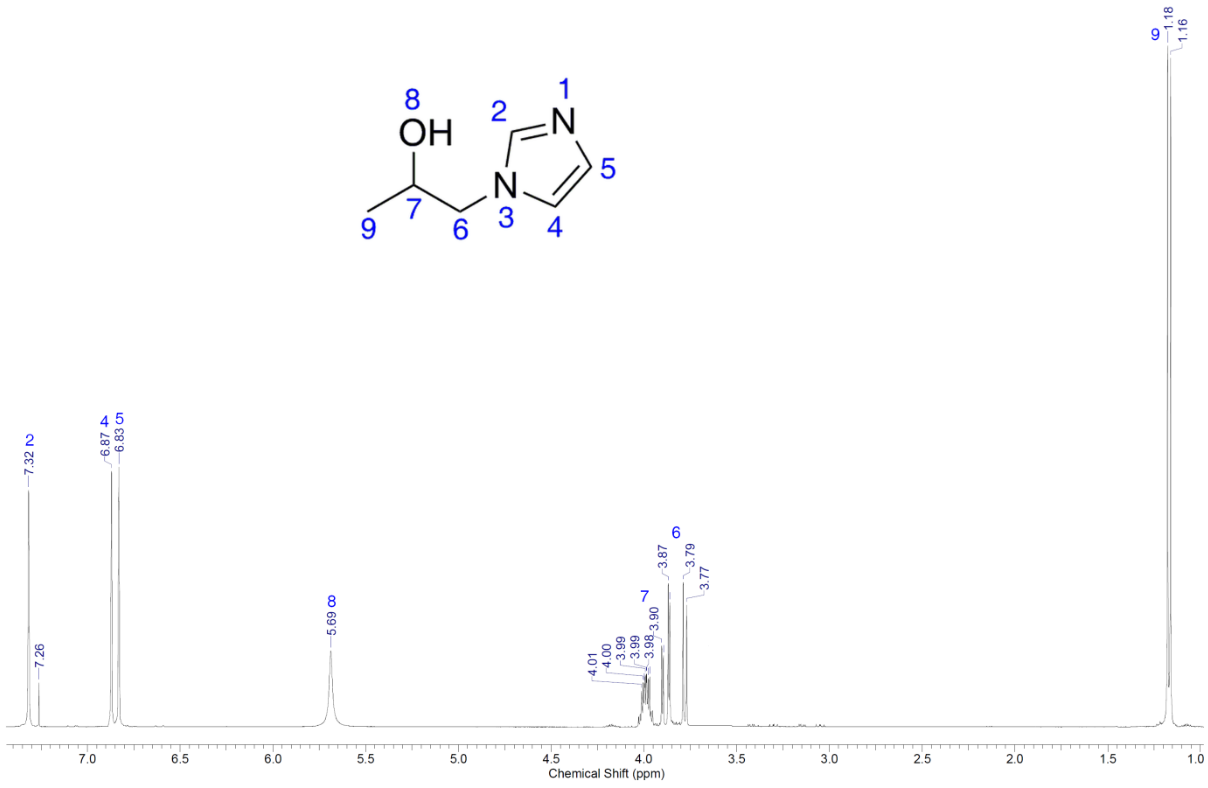

OH
N
N
8
2
1
5
9
7
6
3
4
7.32
7.26
6.87
6.83
5.69
4.01
4.00
3.99
3.99
3.98
3.90
3.87
3.79
3.77
1.18
1.16
Chemical Shift (ppm)

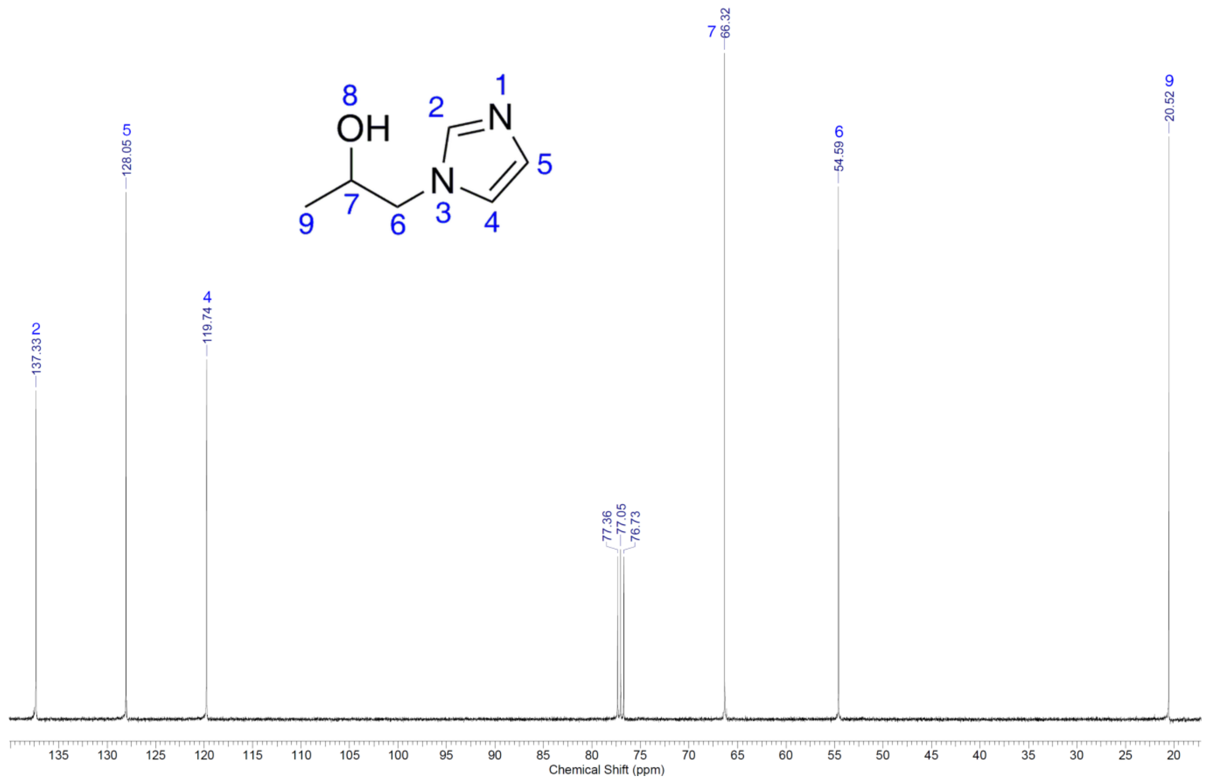

OH
N
N
8
2
1
5
9
7
6
3
4
137.33
128.05
119.74
77.36
77.05
76.73
66.32
54.59
20.52
Chemical Shift (ppm)

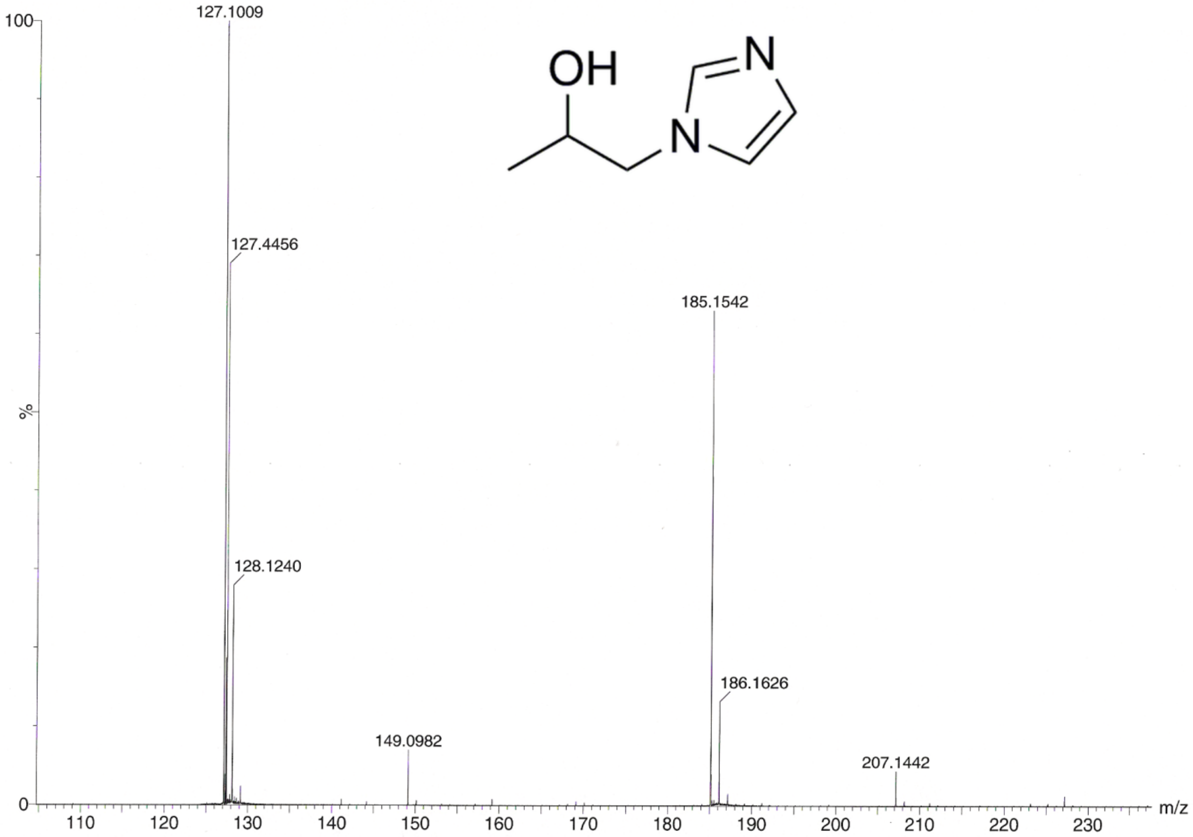

100
%
0
127.1009
127.4456
128.1240
149.0982
185.1542
186.1626
207.1442
110
120
130
140
150
160
170
180
190
200
210
220
230
m/z
OH
N
N

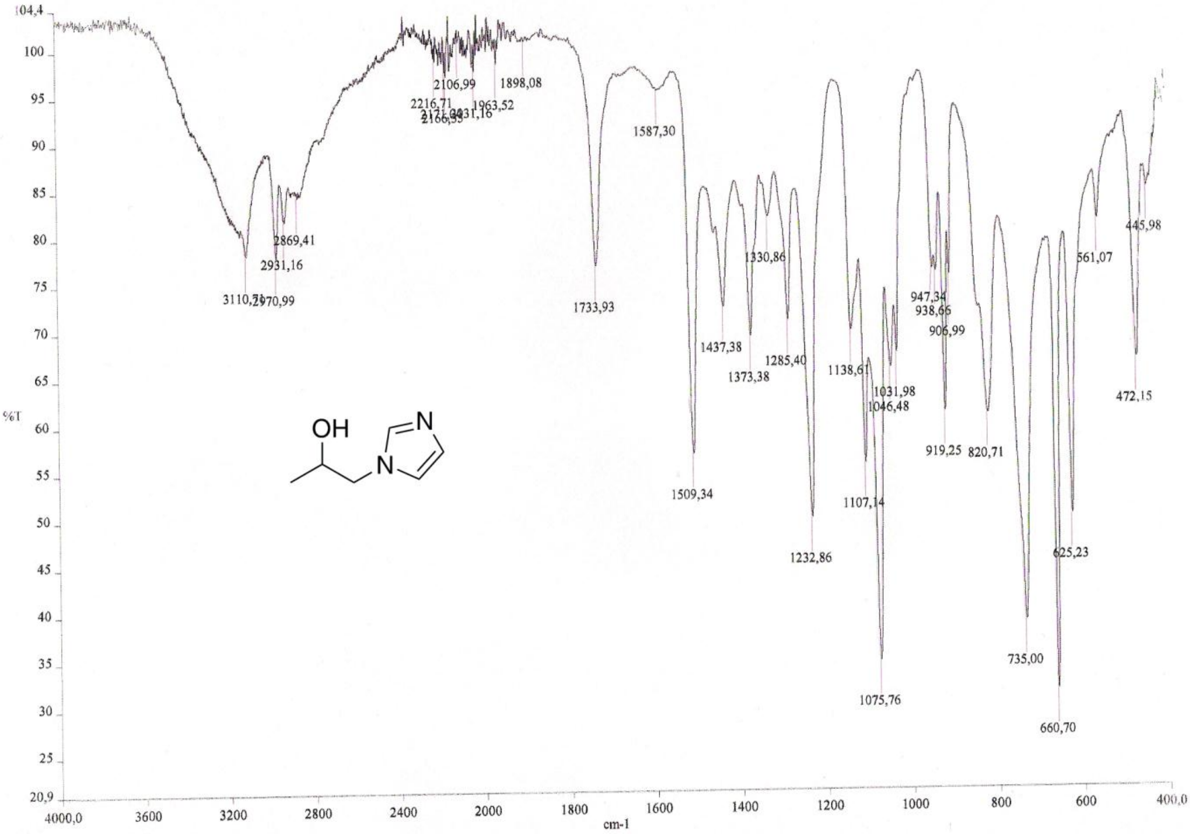

104,4
%T
20,9
4000,0
3600
3200
2800
2400
2000
1800
1600
1400
1200
1000
800
600
400,0
cm-1
3110,71
2970,99
2931,16
2869,41
1733,93
1587,30
1509,34
1437,38
1373,38
1330,86
1285,40
1232,86
1138,61
1107,14
1075,76
1046,48
1031,98
947,34
938,66
919,25
906,99
820,71
735,00
660,70
625,23
561,07
472,15
445,98
OH
N
N

1-(1*H*-1,2,4-Triazol-1-yl)propan-2-ol (±)-3b:

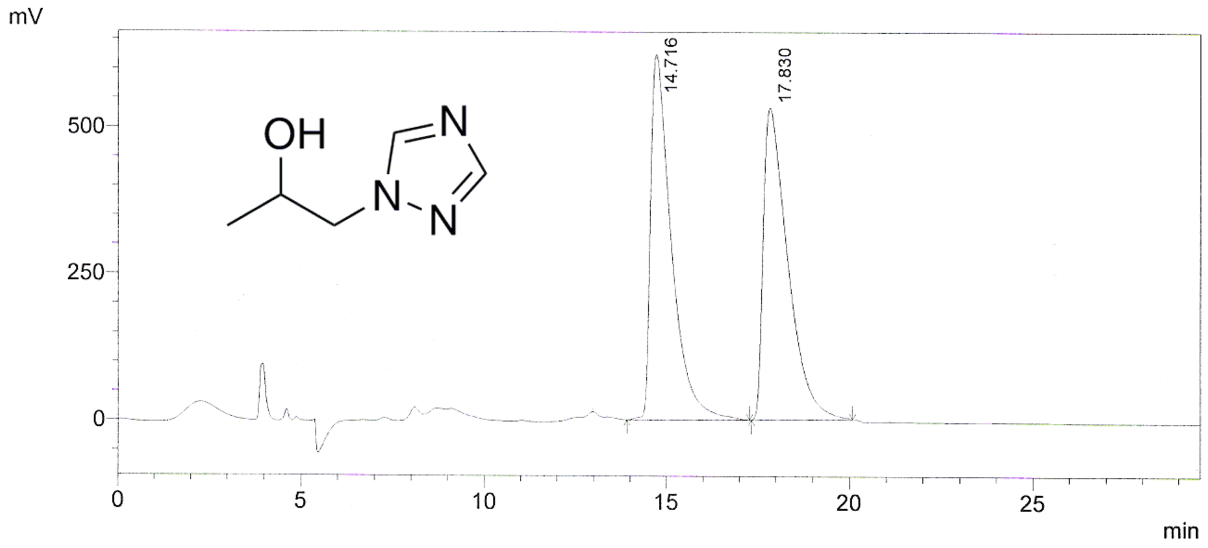

(*S*)-(+)-1-(1*H*-1,2,4-Triazol-1-yl)propan-2-ol [(*S*)-(+)-5b]

$[\alpha]_D^{29}$ = +44 (*c* 1.0, $CHCl_3$) for ee = 98%

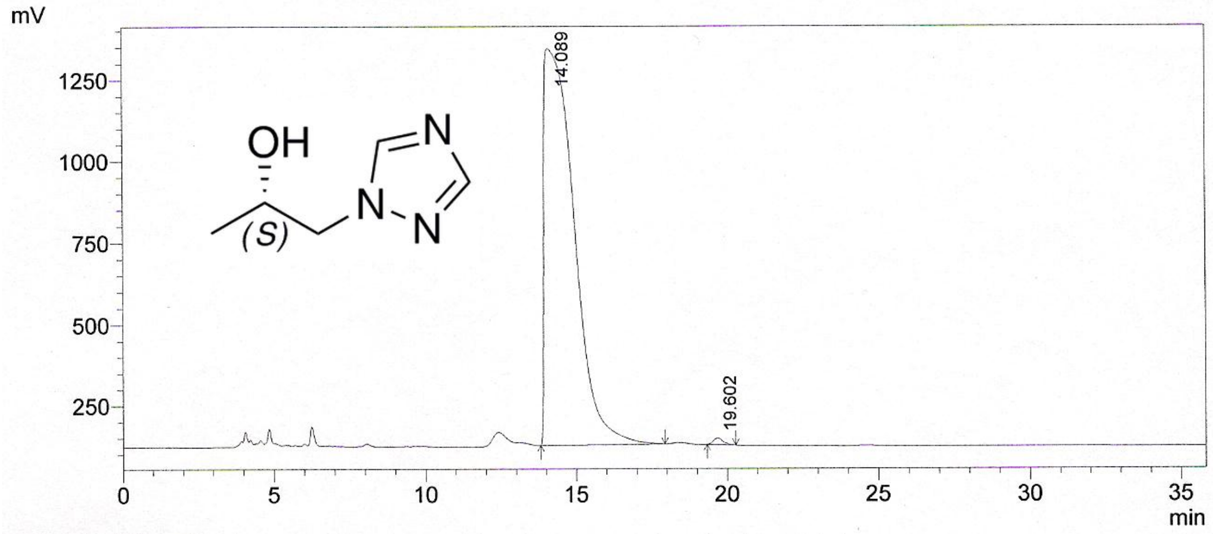

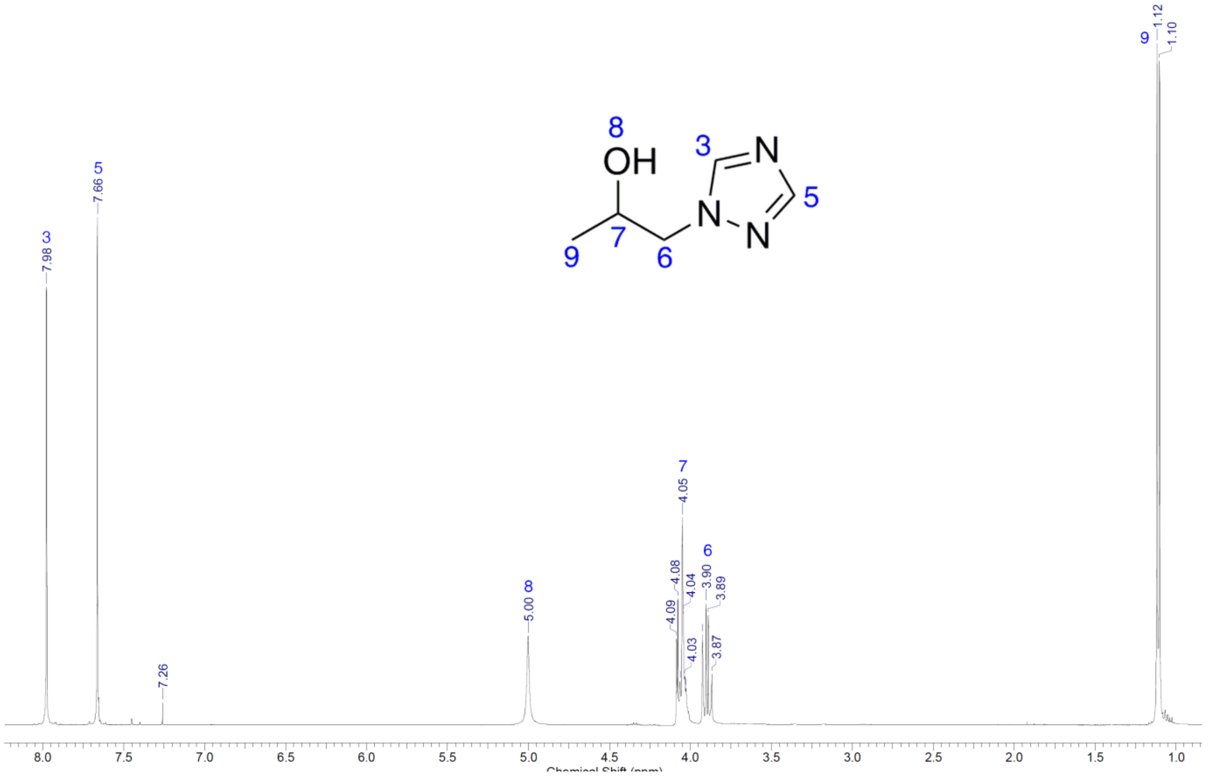
9 1.12
1.10
8
OH
3
N
5
N
N
9
7
6
7.66 5
7.98 3
7.26
5.00 8
7 4.05
4.08
4.09
4.04
4.03
6 3.90
3.89
3.87
8.0 7.5 7.0 6.5 6.0 5.5 5.0 4.5 4.0 3.5 3.0 2.5 2.0 1.5 1.0

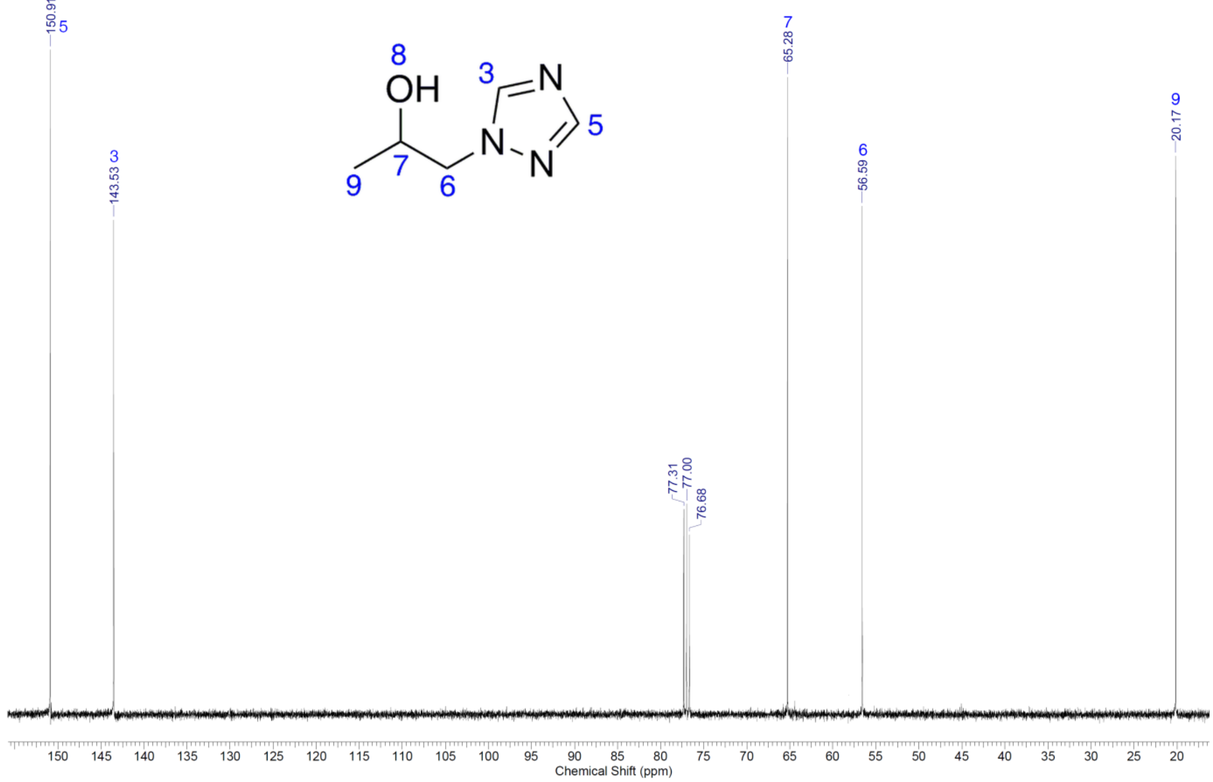
150.91 5
143.53 3
8
OH
3
N
5
N
N
9
7
6
65.28 7
56.59 6
20.17 9
77.31
77.00
76.68
150 145 140 135 130 125 120 115 110 105 100 95 90 85 80 75 70 65 60 55 50 45 40 35 30 25 20
Chemical Shift (ppm)

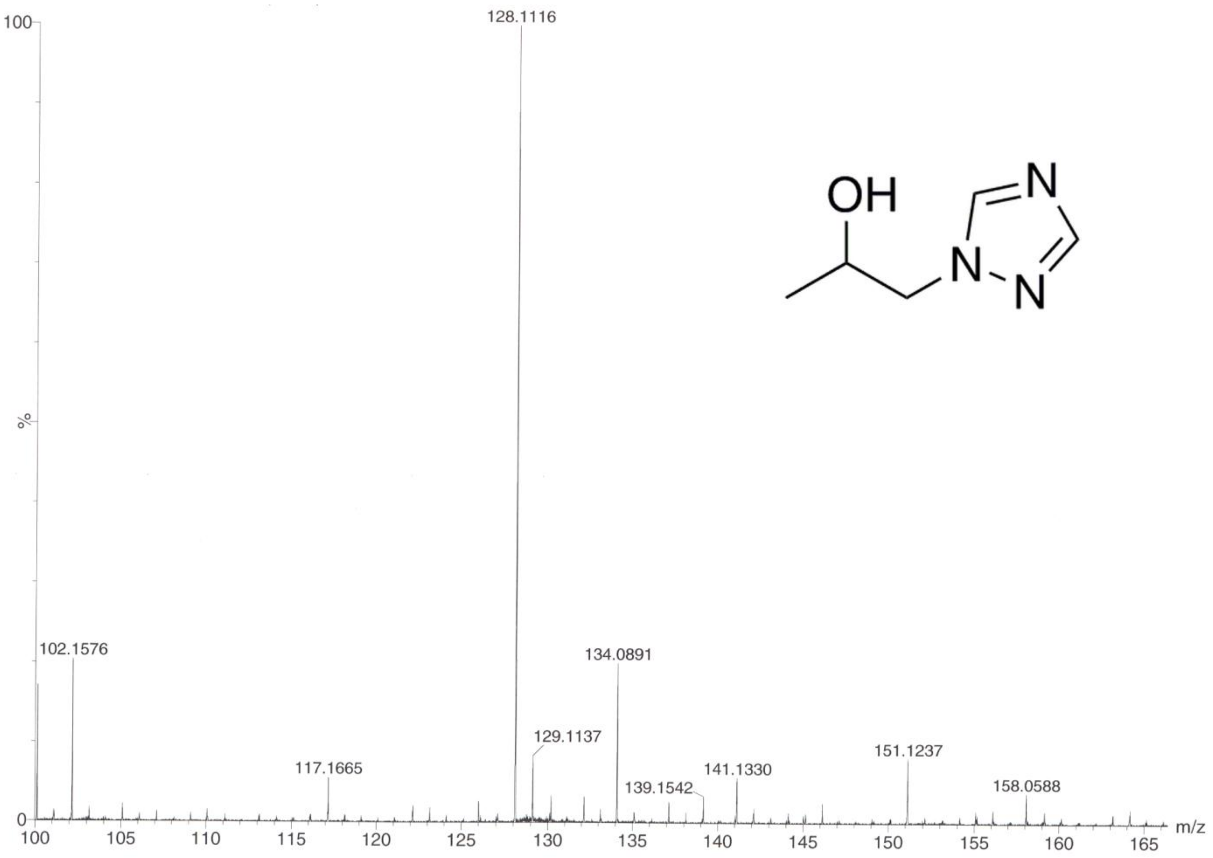

128.1116
102.1576
134.0891
129.1137
117.1665
139.1542
141.1330
151.1237
158.0588
%
m/z

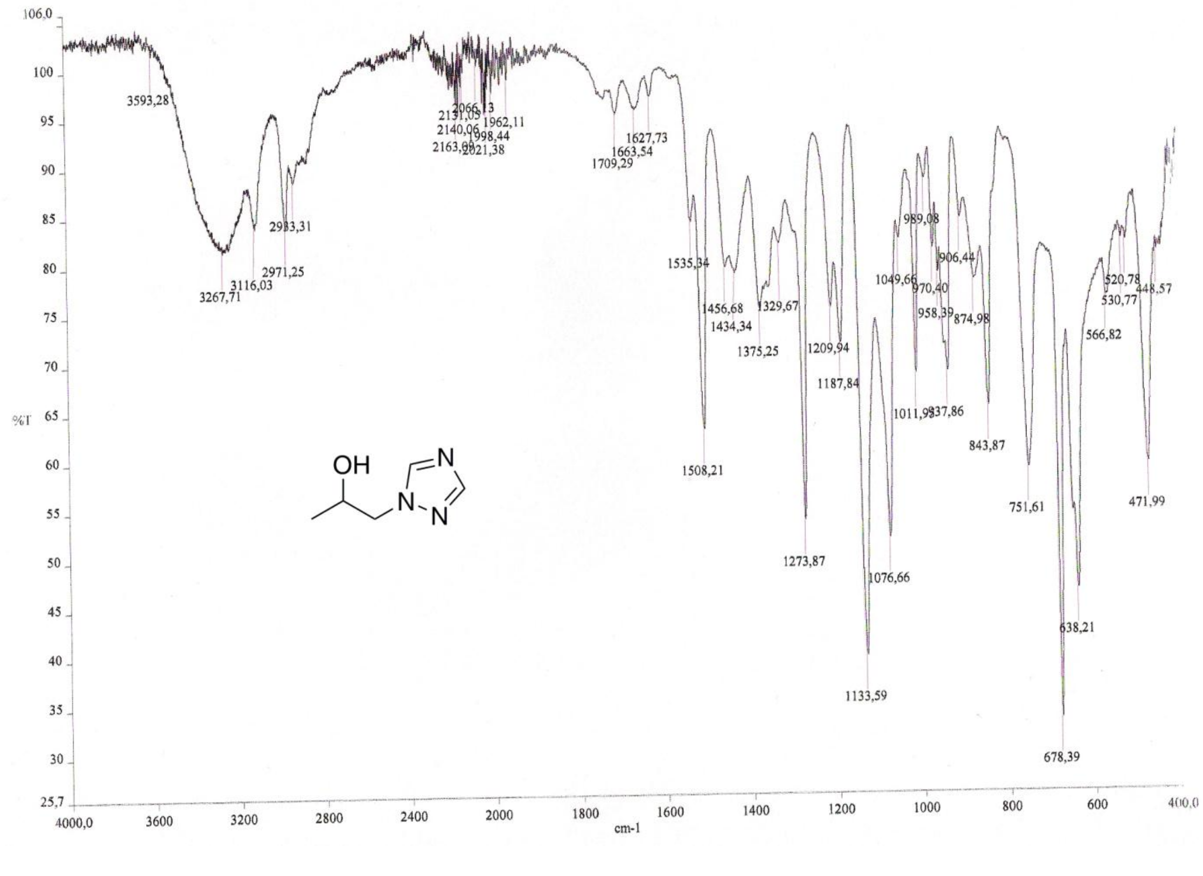

%T
cm-1
3593,28
3267,71
3116,03
2971,25
1709,29
1663,54
1627,73
1535,34
1508,21
1456,68
1434,34
1375,25
1329,67
1273,87
1209,94
1187,84
1133,59
1076,66
1049,66
1011,98
989,08
970,40
958,39
906,44
874,98
843,87
751,61
678,39
638,21
566,82
530,77
520,78
471,99
448,57

1-(1*H*-Imidazol-1-yl)propan-2-yl acetate (±)-4a:

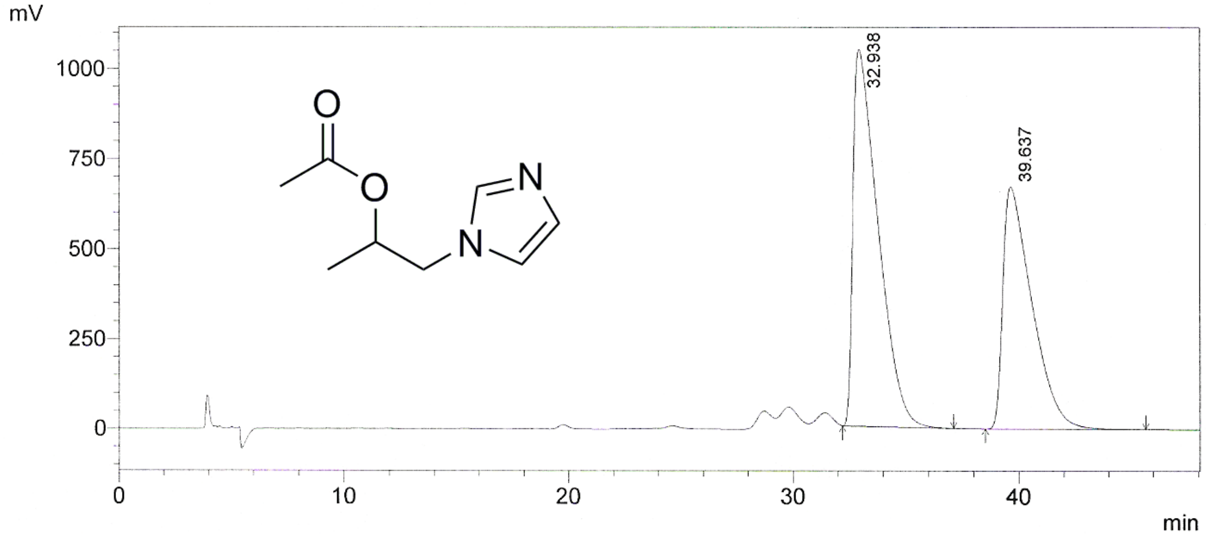

(*R*)-(+)-1-(1*H*-Imidazol-1-yl)propan-2-yl acetate [(*R*)-(+)-6a]

$[\alpha]_D^{29}$ = +9.2 (*c* 1.0, $CHCl_3$) for ee > 99%

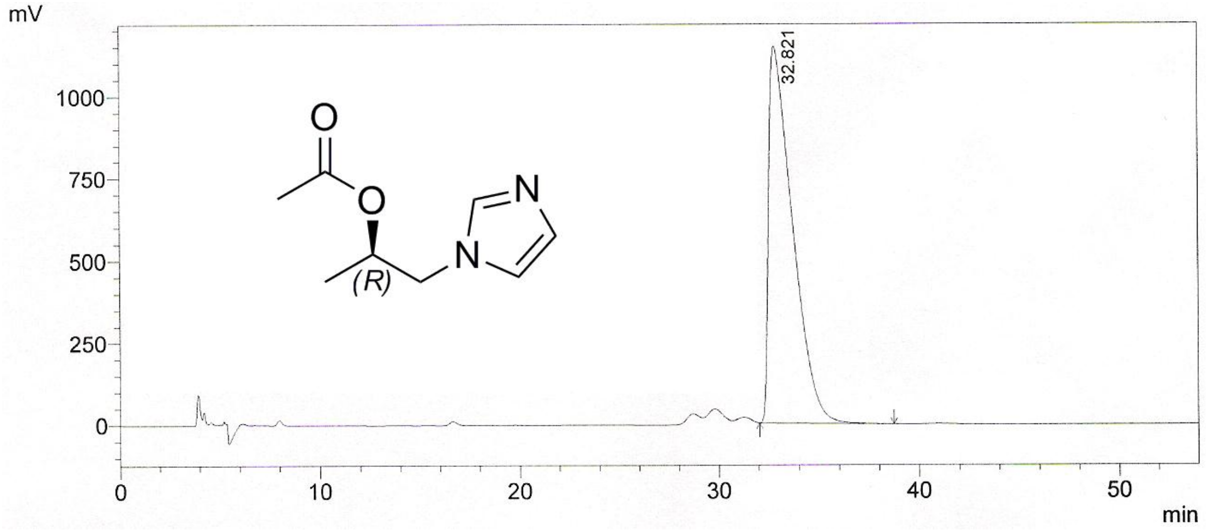

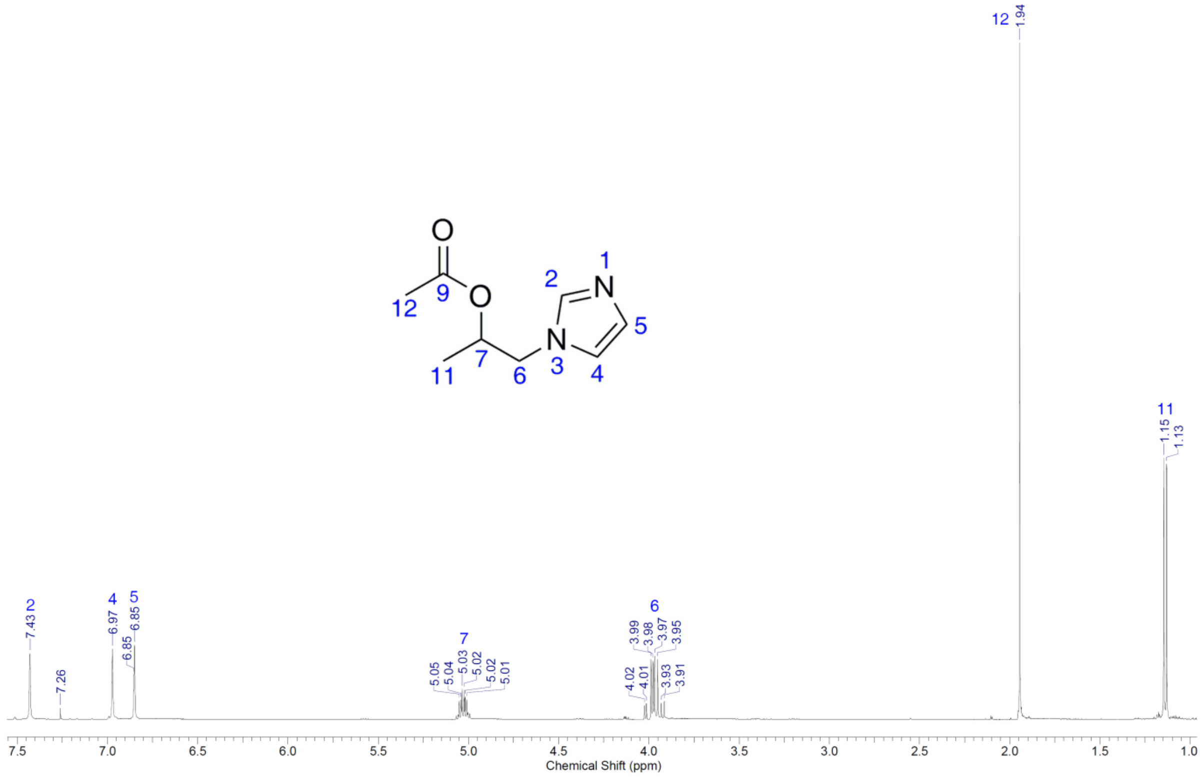
12 1.94
11 1.15 1.13
7.43 2
7.26
4 6.97
5 6.85 6.85
7 5.05 5.04 5.03 5.02 5.02 5.01
6 3.99 3.98 3.97 3.95 4.02 4.01 3.93 3.91
O
9
12
O
2
1
N
5
N
3
4
7
11
6
7.5 7.0 6.5 6.0 5.5 5.0 4.5 4.0 3.5 3.0 2.5 2.0 1.5 1.0
Chemical Shift (ppm)

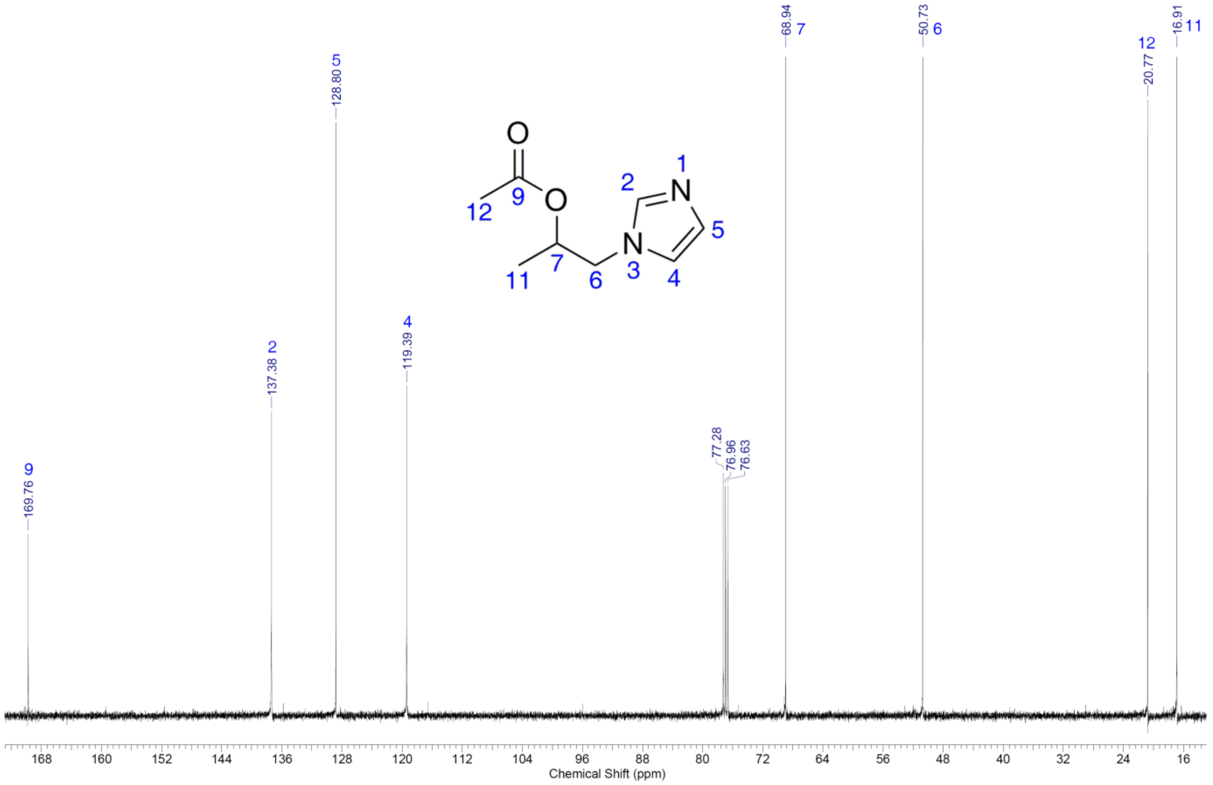
68.94 7
50.73 6
12 20.77
16.91 11
128.80 5
137.38 2
119.39 4
77.28 76.96 76.63
169.76 9
O
9
12
O
2
1
N
5
N
3
4
7
11
6
168 160 152 144 136 128 120 112 104 96 88 80 72 64 56 48 40 32 24 16
Chemical Shift (ppm)

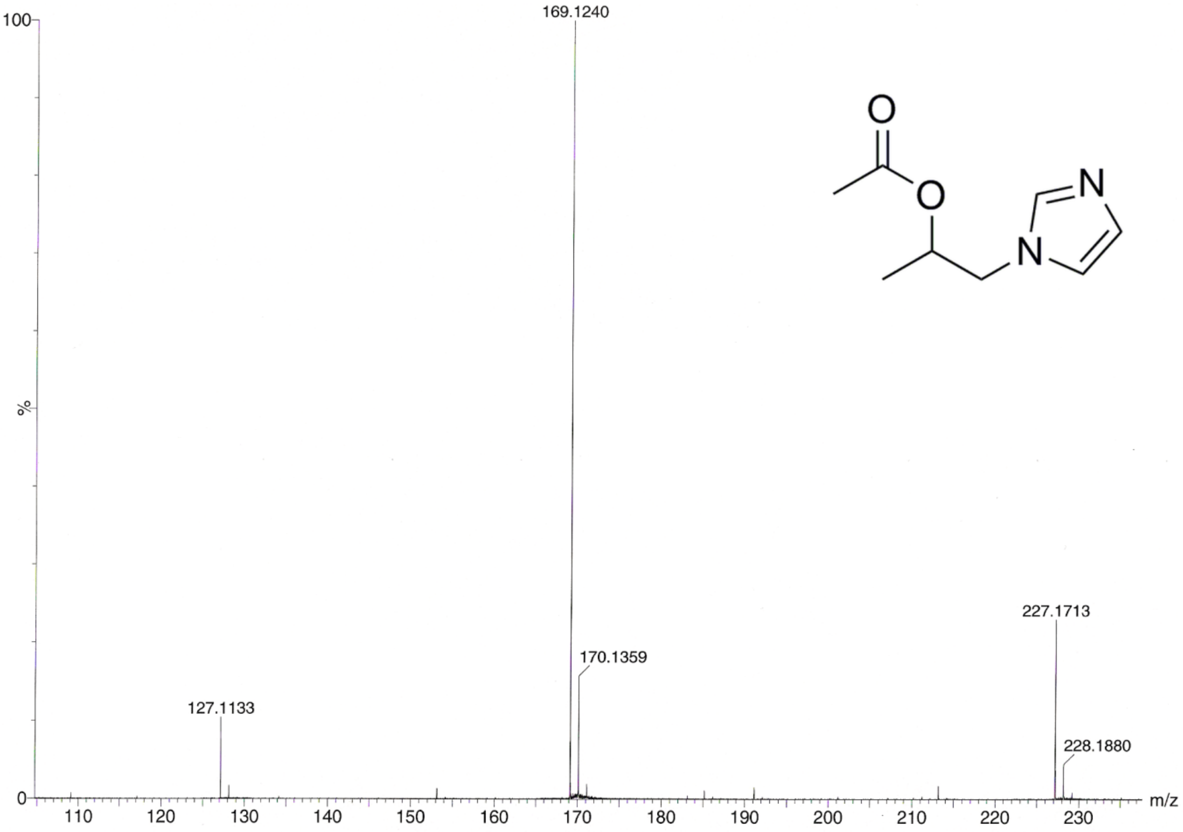

169.1240
227.1713
170.1359
127.1133
228.1880
100
%
0
m/z
110
120
130
140
150
160
170
180
190
200
210
220
230
O
O
N
N

1-(1*H*-1,2,4-Triazol-1-yl)propan-2-yl acetate (±)-4b:

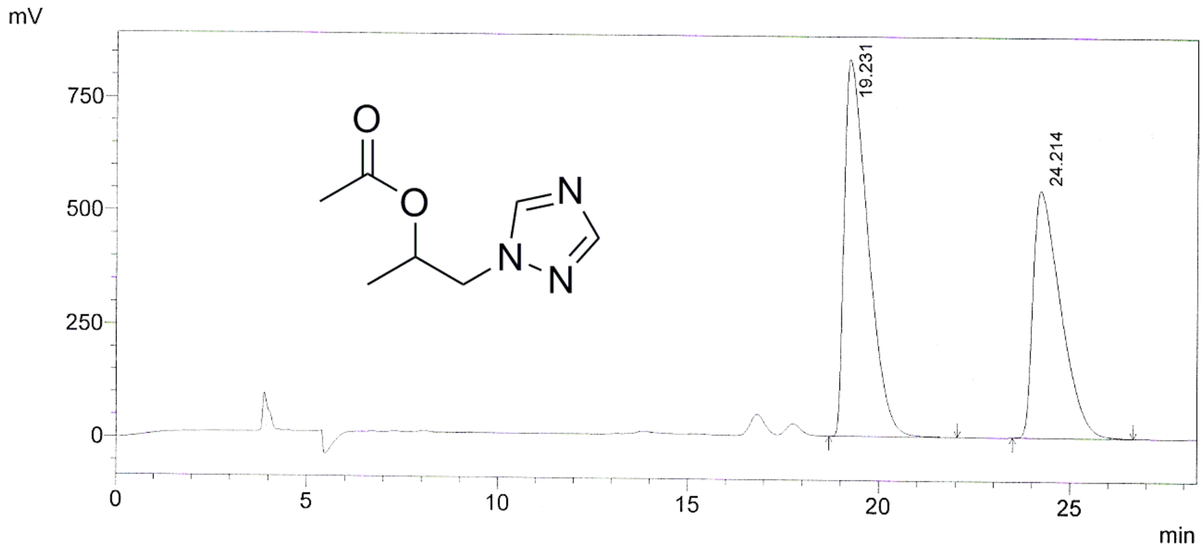

(*R*)-(+)-1-(1*H*-1,2,4-Triazol-1-yl)propan-2-yl acetate [(*R*)-(+)-6b]

$[\alpha]_D^{29}$ = +5.2 (*c* 1.0, $CHCl_3$) for ee = 90%

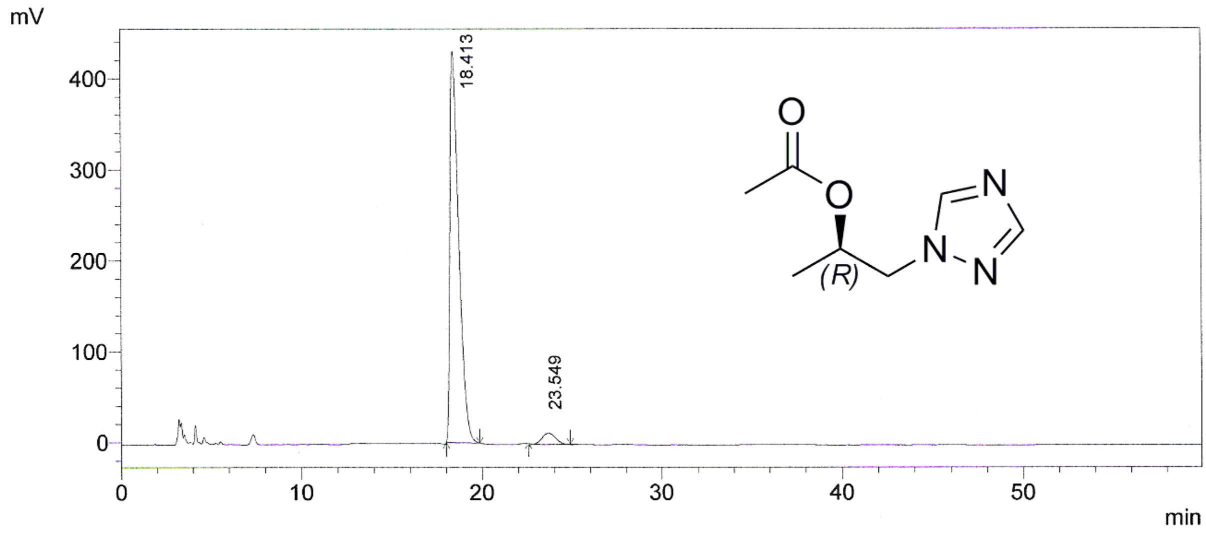

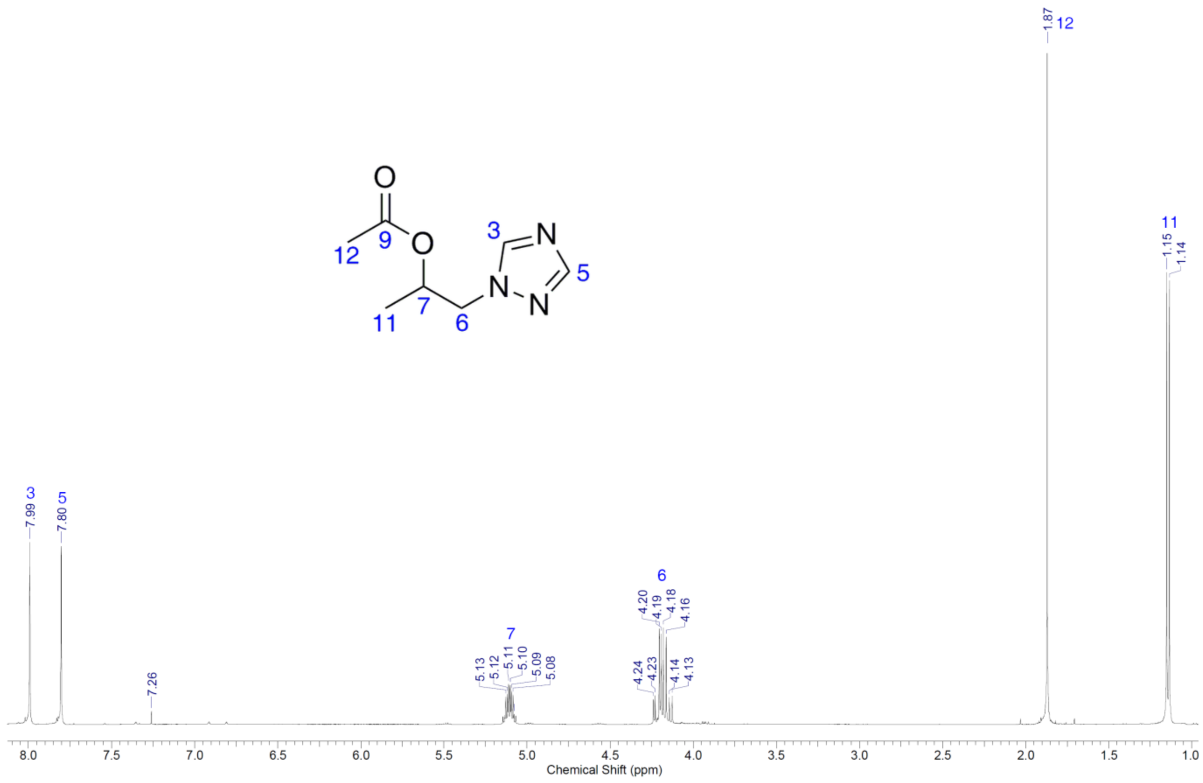

O
9
12
O
3
N
5
N
N
7
11
6
1.87
12
11
1.15
1.14
3
7.99
5
7.80
7.26
7
5.13
5.12
5.11
5.10
5.09
5.08
6
4.20
4.19
4.18
4.16
4.24
4.23
4.14
4.13
8.0
7.5
7.0
6.5
6.0
5.5
5.0
4.5
4.0
3.5
3.0
2.5
2.0
1.5
1.0
Chemical Shift (ppm)

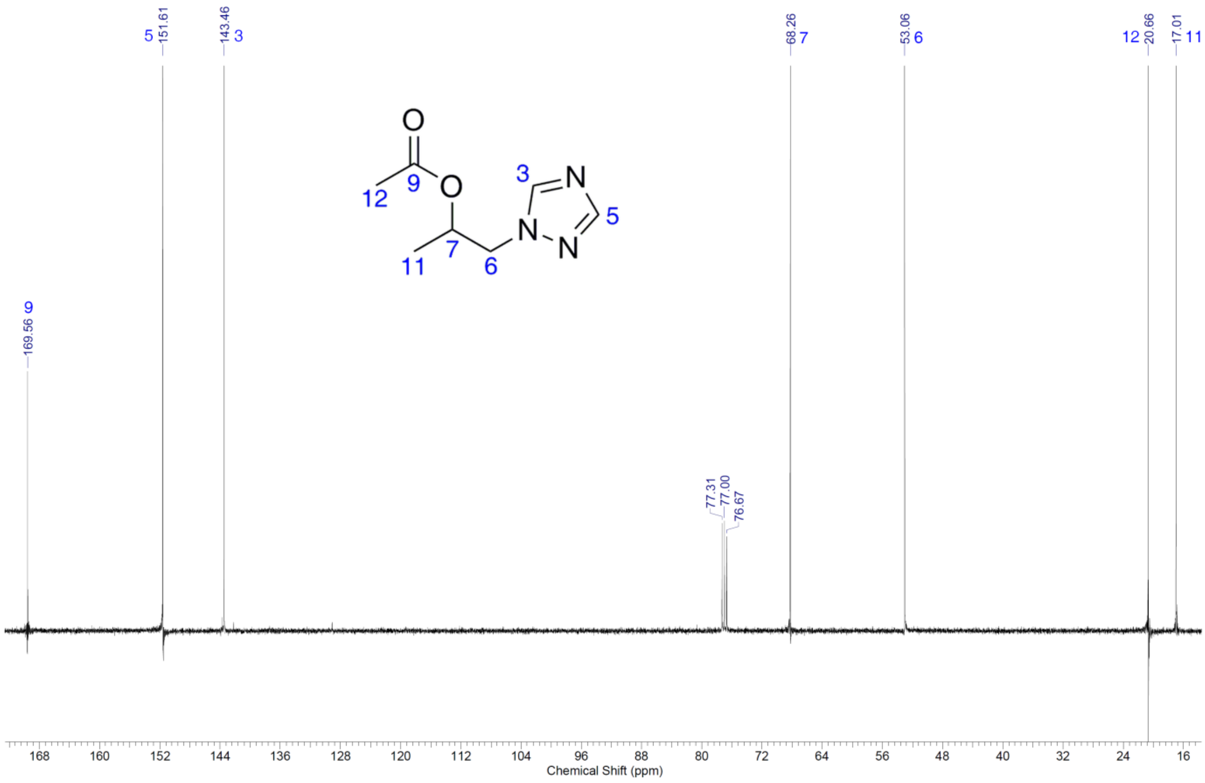

5
151.61
143.46
3
68.26
7
53.06
6
12
20.66
17.01
11
9
169.56
77.31
77.00
76.67
168
160
152
144
136
128
120
112
104
96
88
80
72
64
56
48
40
32
24
16
Chemical Shift (ppm)

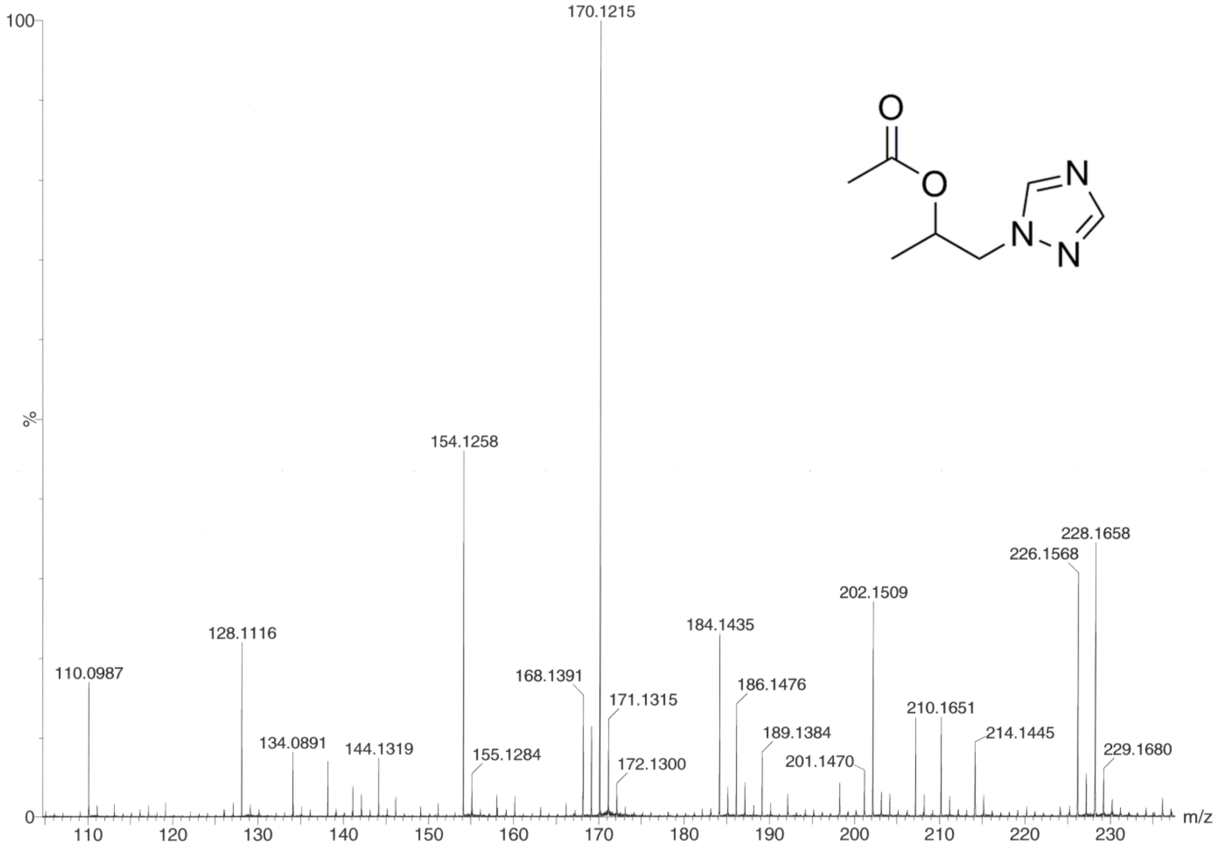
170.1215
154.1258
228.1658
226.1568
202.1509
184.1435
128.1116
110.0987
168.1391
186.1476
171.1315
210.1651
189.1384
214.1445
134.0891
144.1319
201.1470
229.1680
155.1284
172.1300
100
%
0
110
120
130
140
150
160
170
180
190
200
210
220
230
m/z
O
O
N
N
N

(*S*)-((*S*)-1-(1*H*-Imidazol-1-yl)propan-2-yl) 2-methoxy-2-phenylacetate (9a):

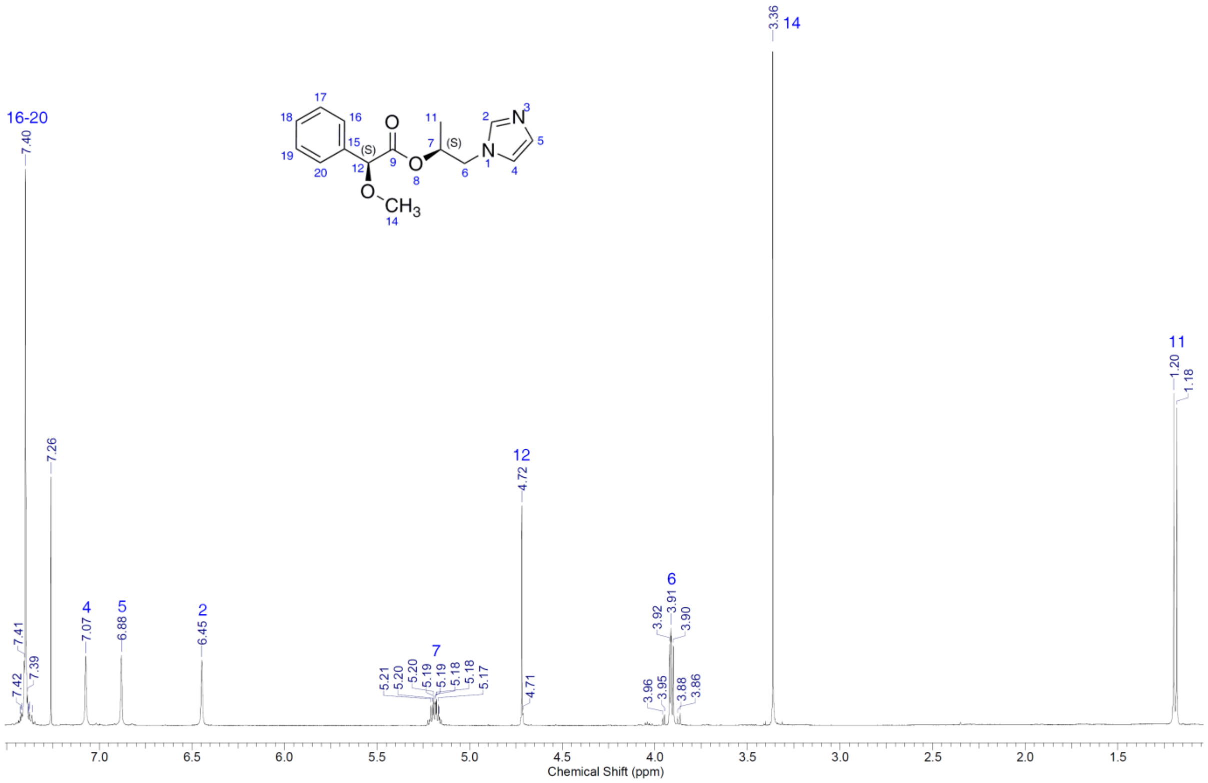

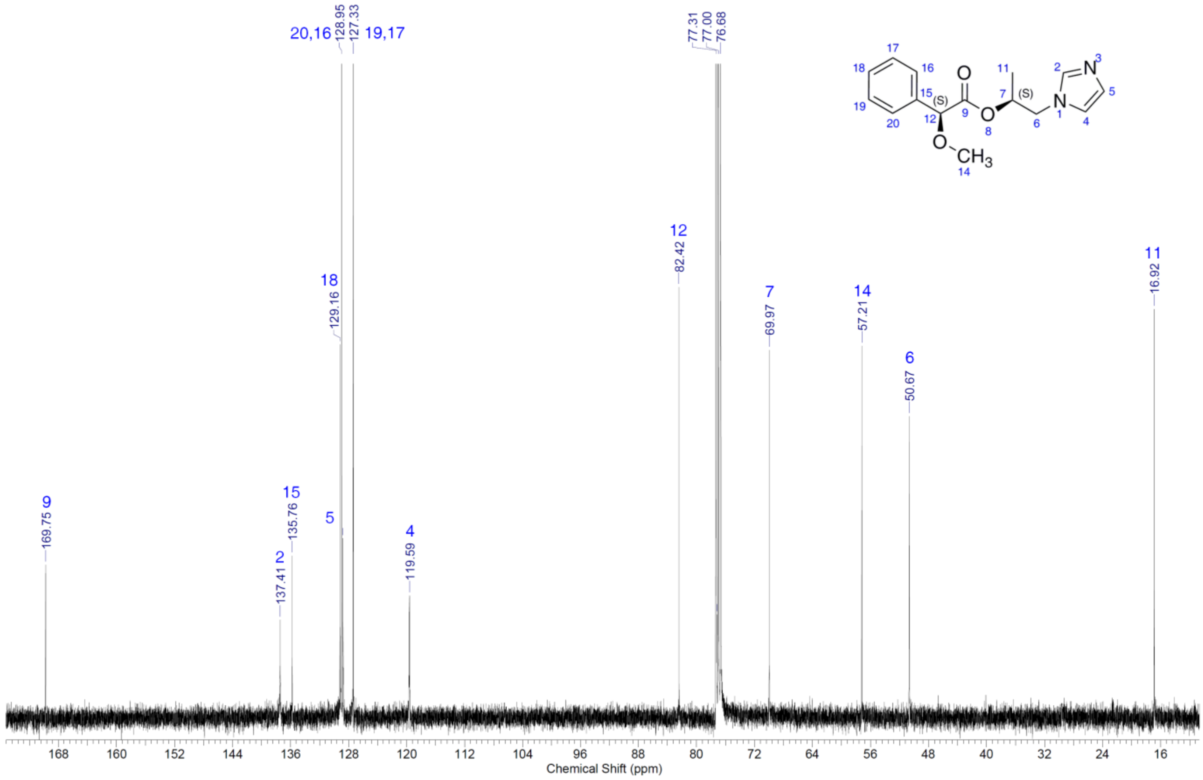

((*R*)-((*S*)-1-(1*H*-Imidazol-1-yl)propan-2-yl) 2-methoxy-2-phenylacetate (10a):

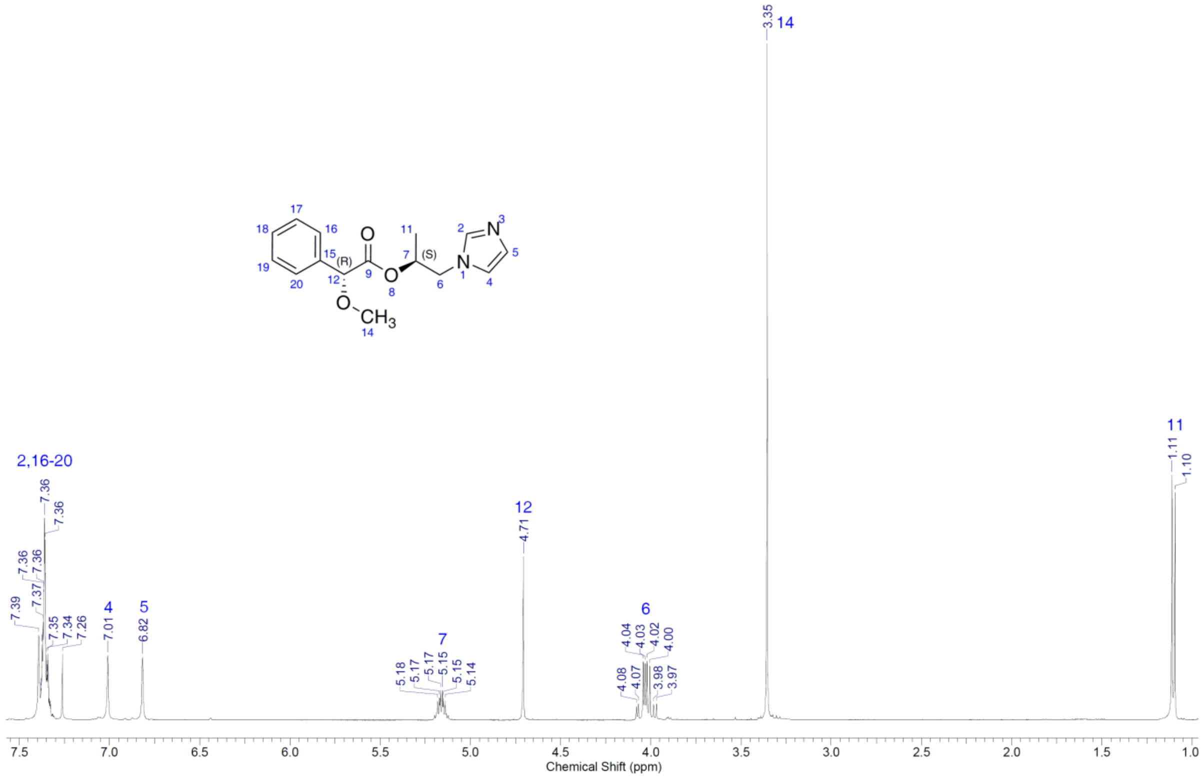

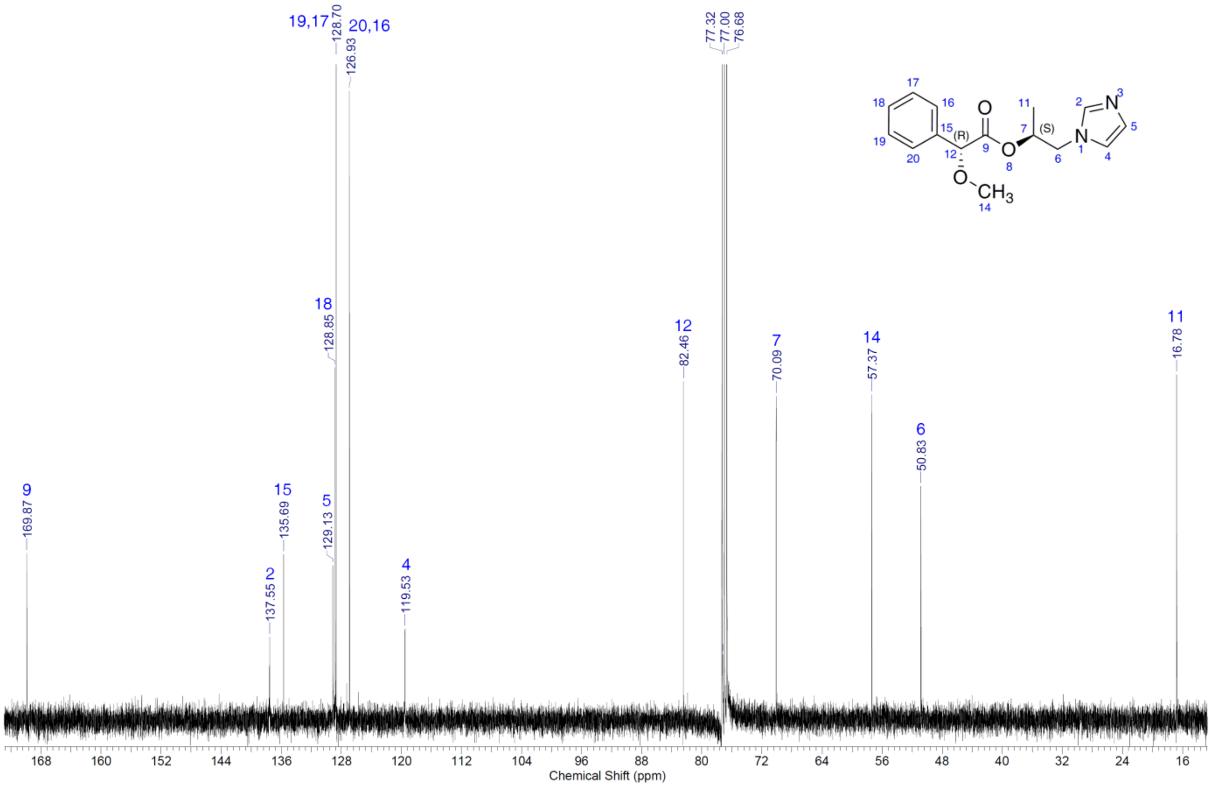

(*S*)-((*S*)-1-(1*H*-1,2,4-Triazol-1-yl)propan-2-yl) 2-methoxy-2-phenylacetate (9b):

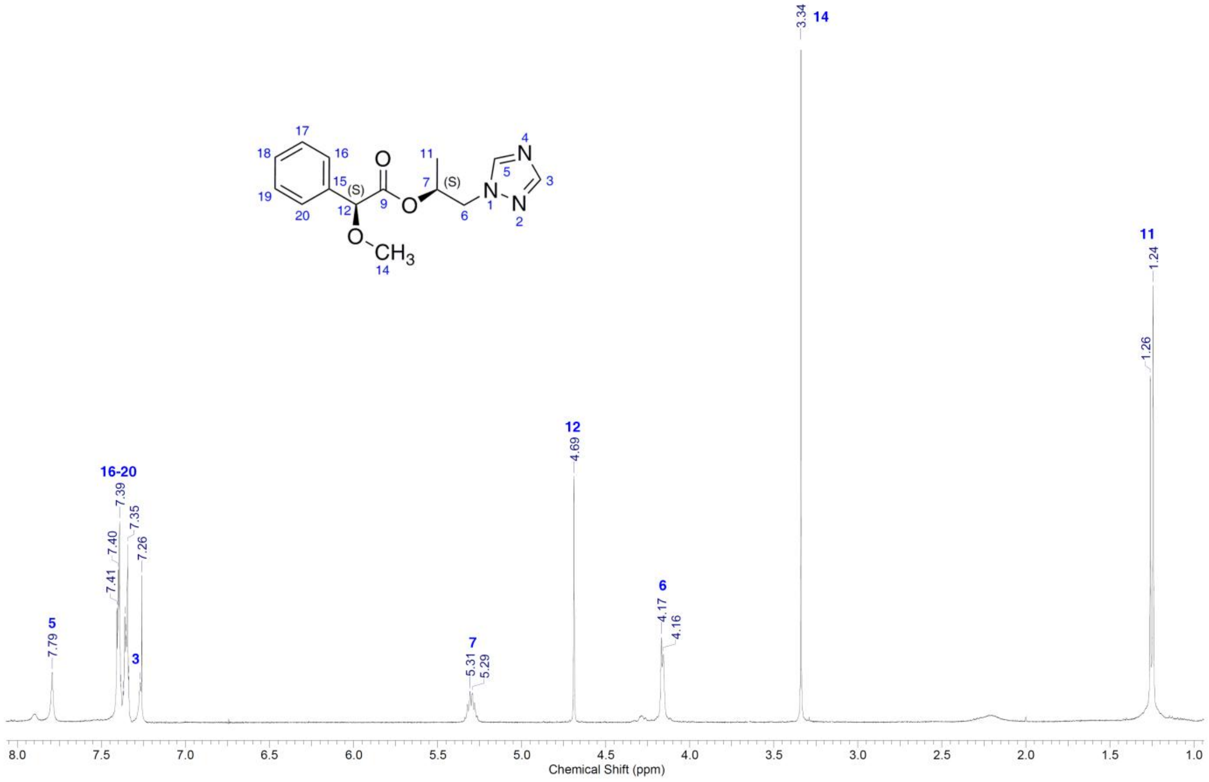

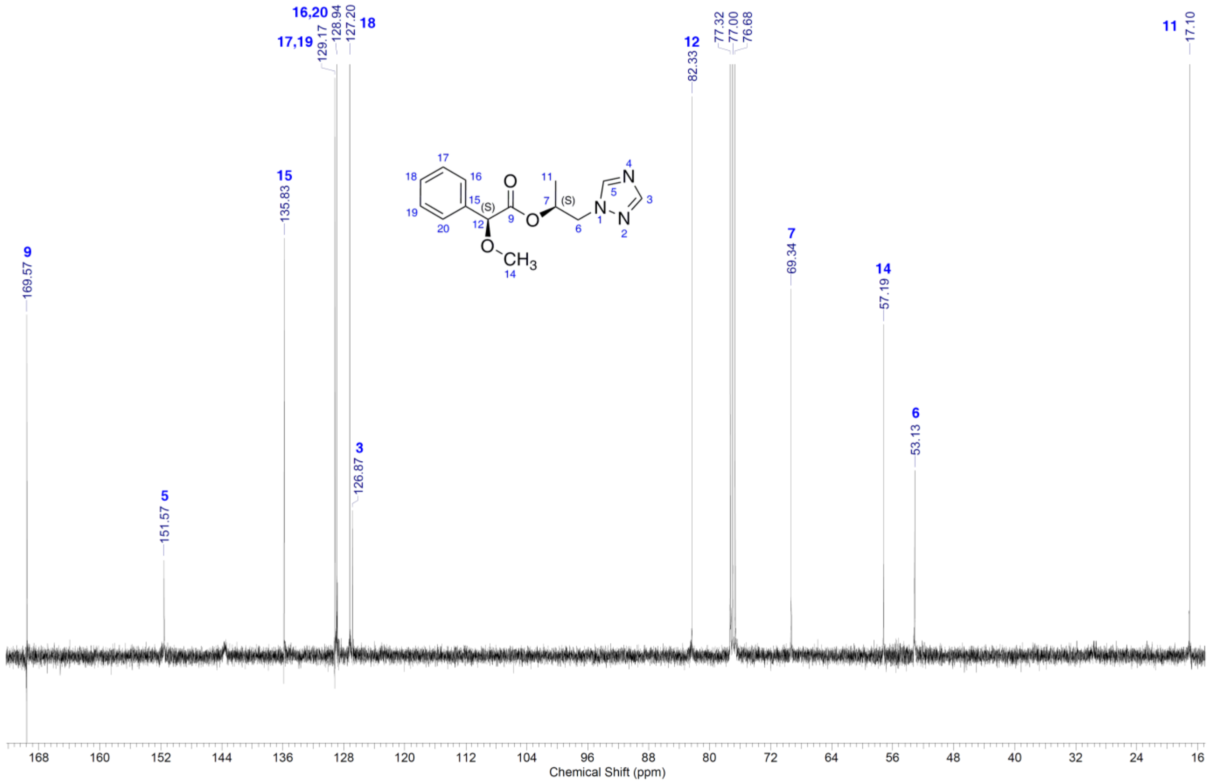

(*R*)-((*S*)-1-(1*H*-1,2,4-Triazol-1-yl)propan-2-yl) 2-methoxy-2-phenylacetate (10b):

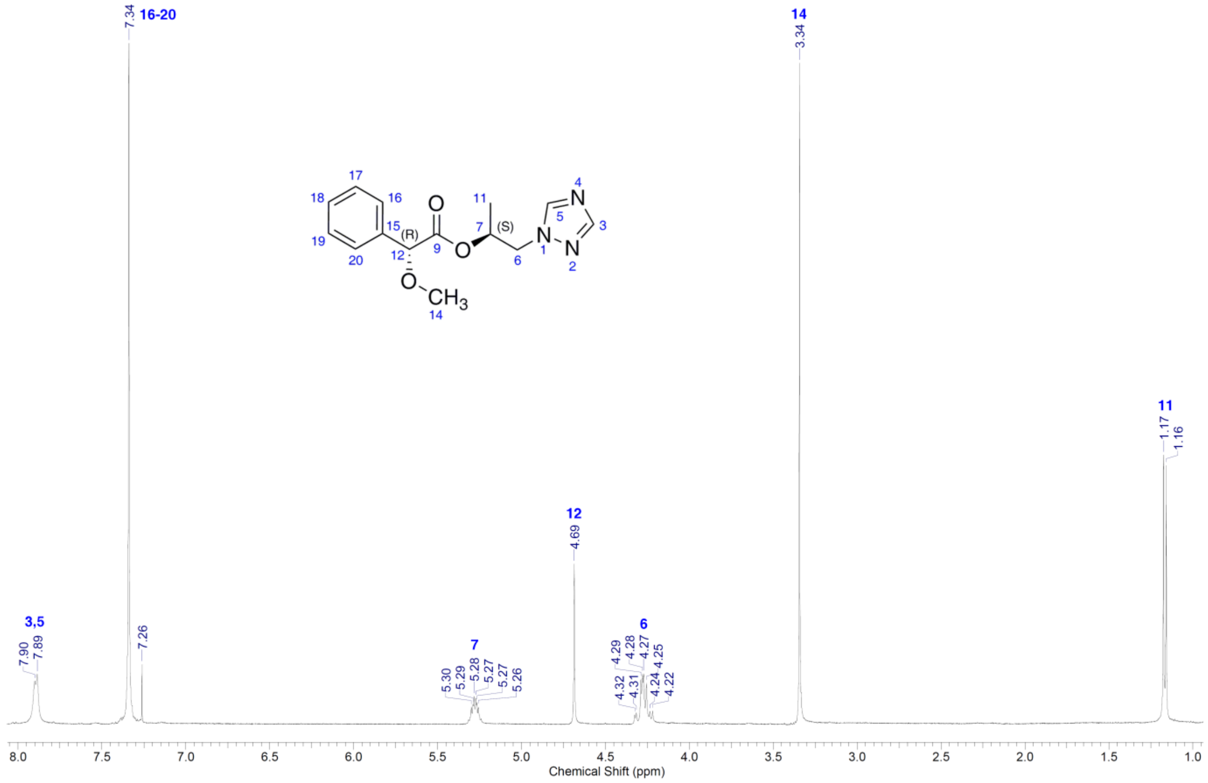

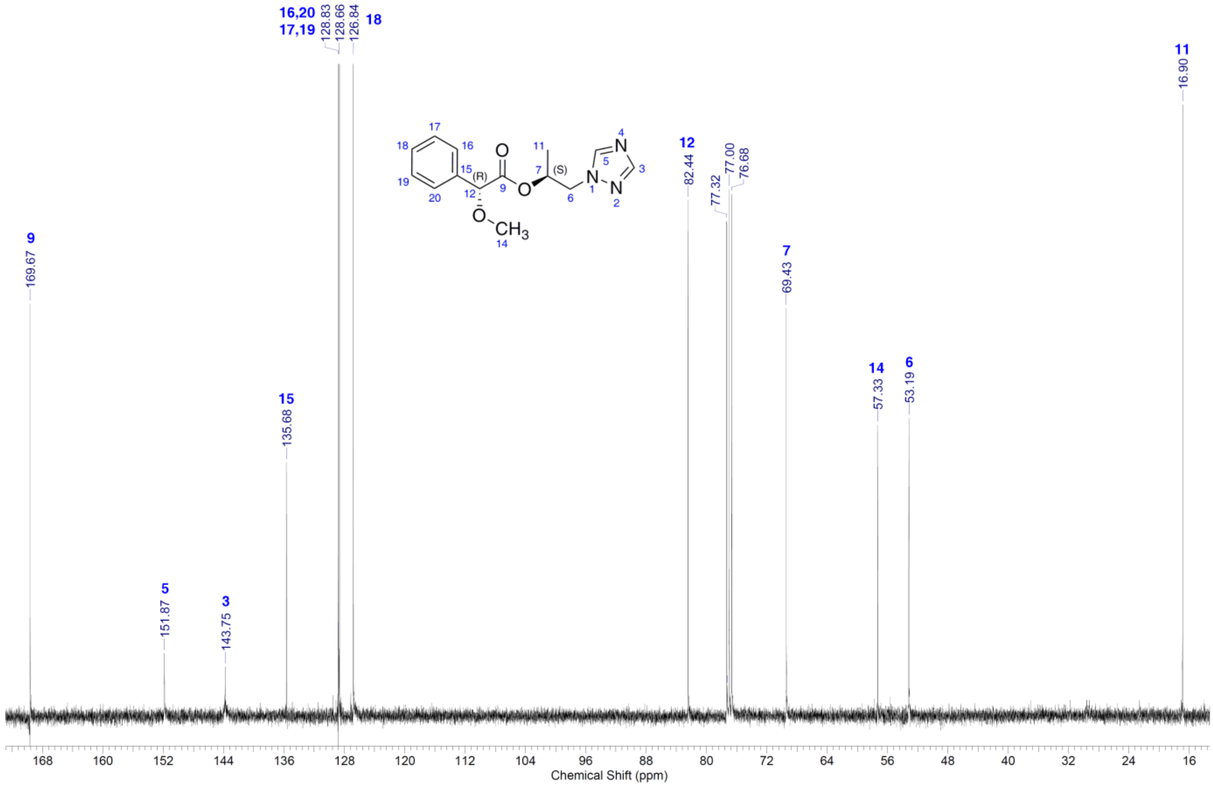

Spectra of the imidazolium salts [(+)-7a–h]

(*S*)-(+)-1-(2-Hydroxypropyl)-3-propyl-1*H*-imidazol-3-ium bromide (+)-7a:

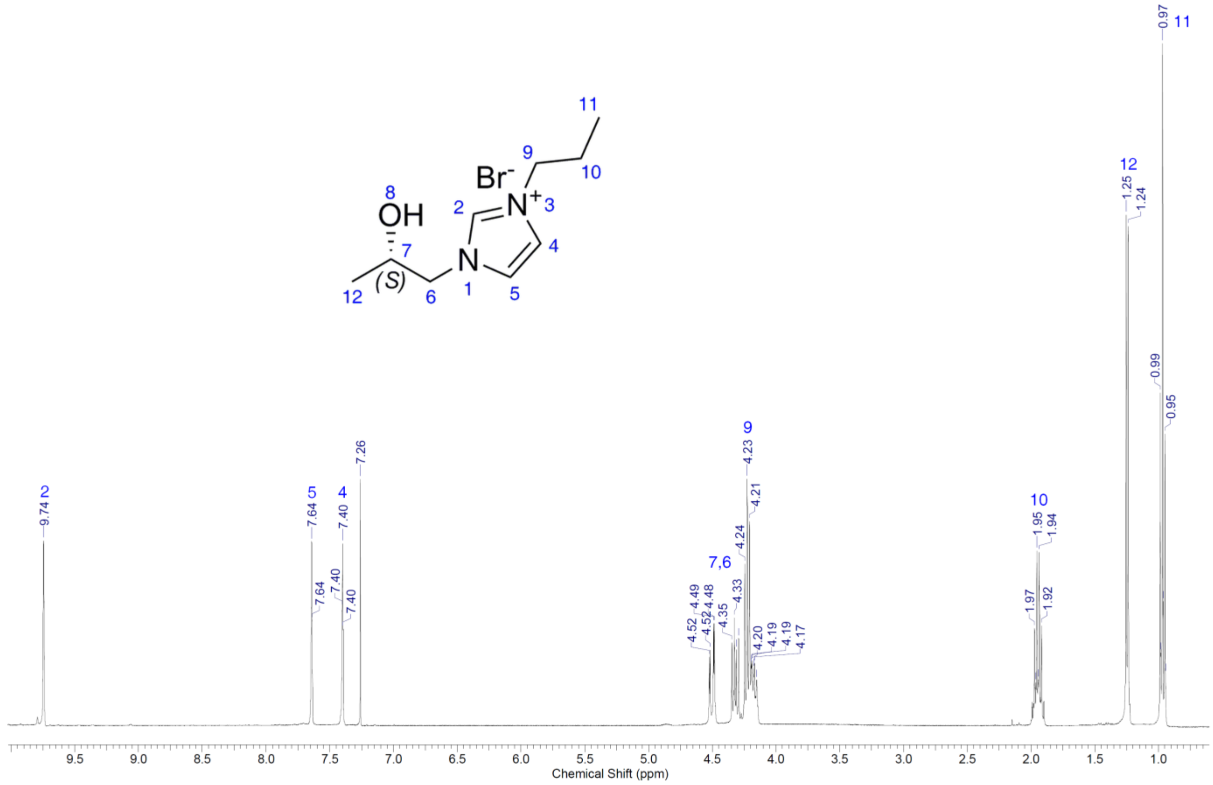

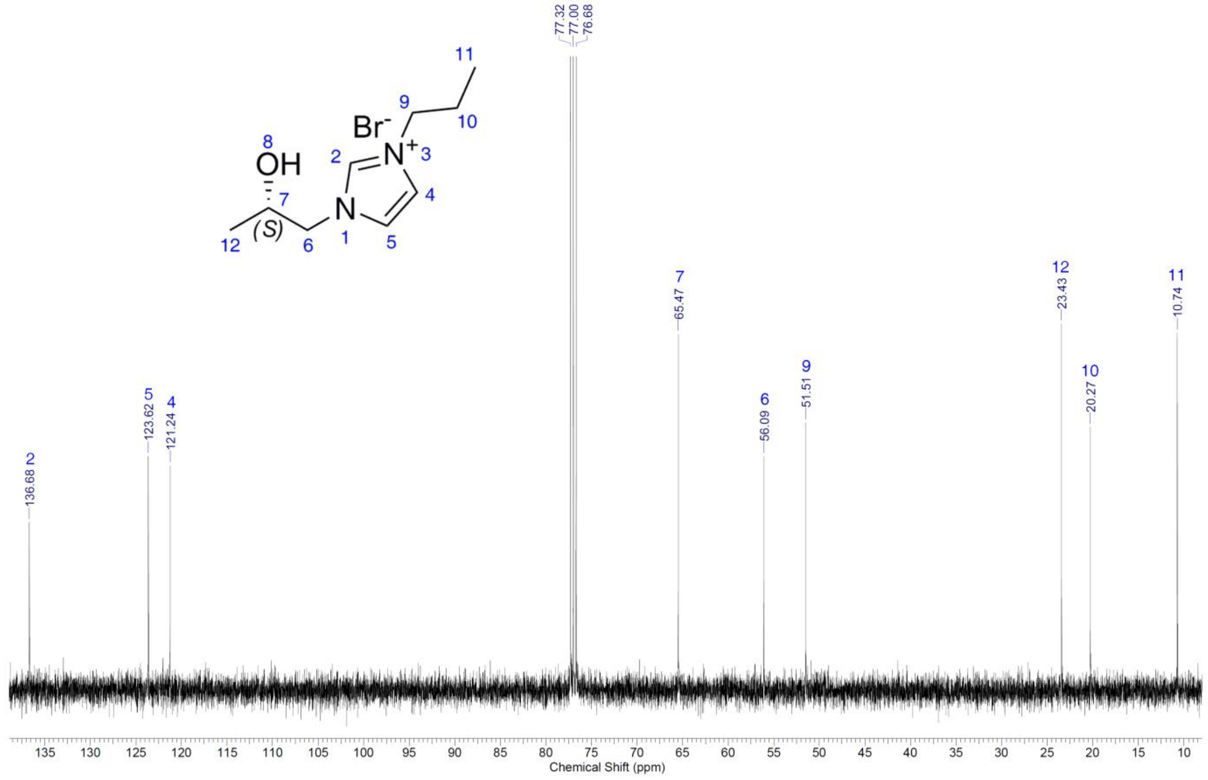

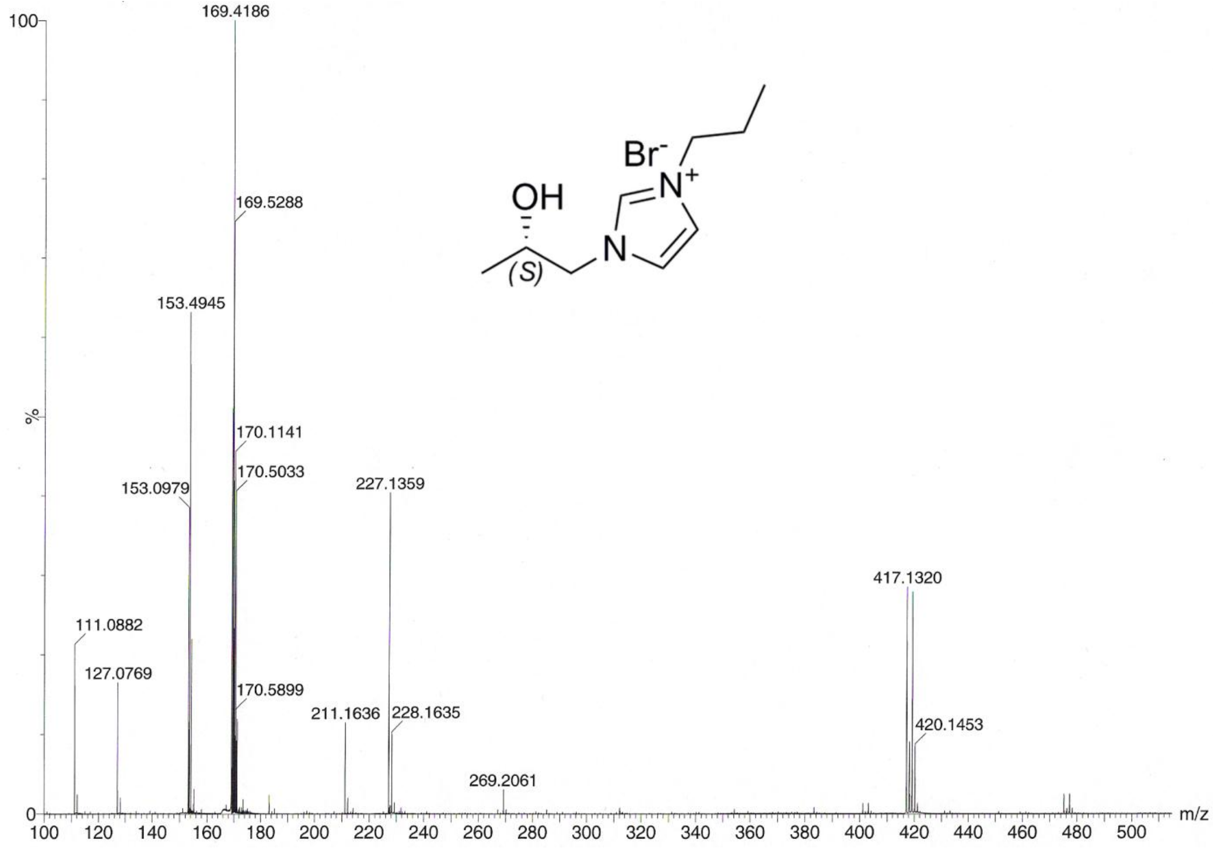

100
%
0
169.4186
169.5288
153.4945
170.1141
170.5033
153.0979
227.1359
417.1320
111.0882
127.0769
170.5899
211.1636
228.1635
420.1453
269.2061
100 120 140 160 180 200 220 240 260 280 300 320 340 360 380 400 420 440 460 480 500
m/z
Br⁻
N⁺
OH
N
(S)

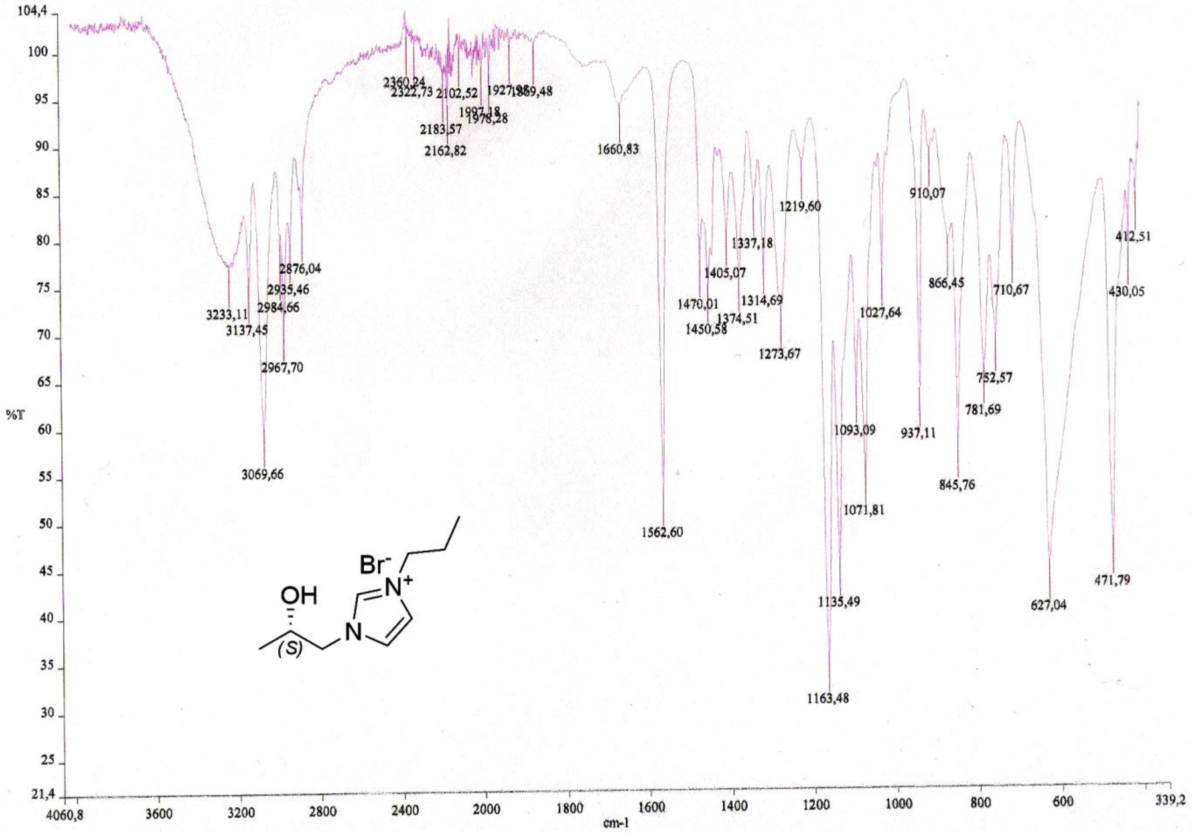

%T
cm-1
4060,8
339,2
104,4
21,4
2360,24
2322,73
2102,52
2183,57
2162,82
1660,83
1562,60
3233,11
3137,45
3069,66
2984,66
2967,70
2935,46
2876,04
1470,01
1450,58
1405,07
1374,51
1337,18
1314,69
1273,67
1219,60
1163,48
1135,49
1093,09
1071,81
1027,64
937,11
910,07
866,45
845,76
781,69
752,57
710,67
627,04
471,79
430,05
412,51
Br⁻
N⁺
OH
N
(S)

(*S*)-(+)-1-(2-Hydroxypropyl)-3-(prop-2-en-1-yl)-1*H*-imidazol-3-ium bromide (+)-7b:

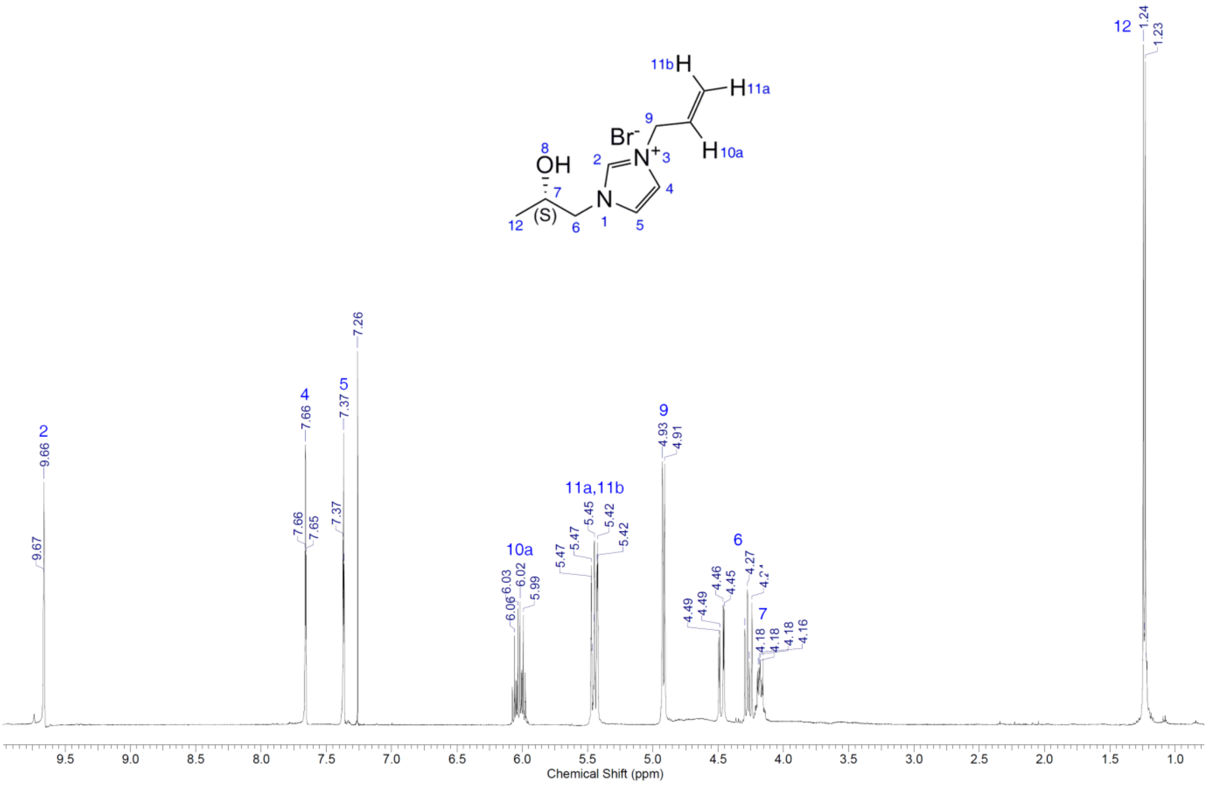

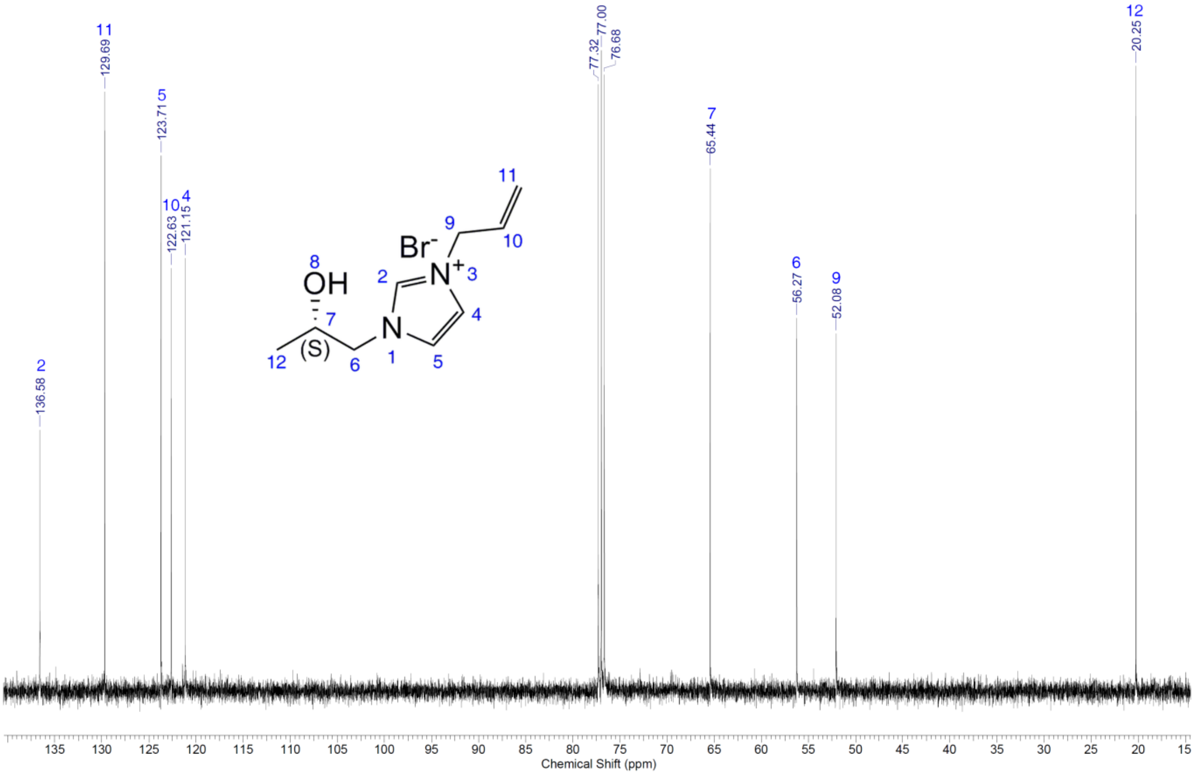

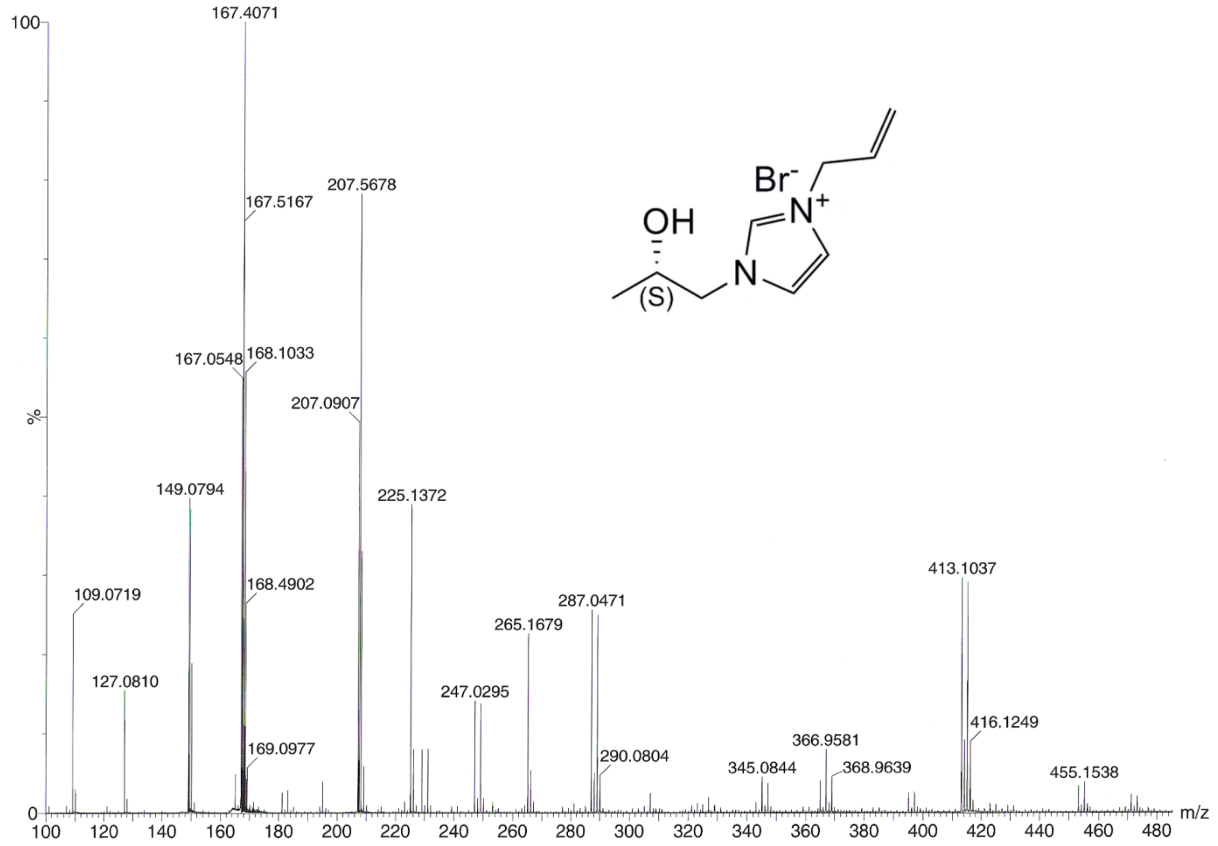

100
%
0
m/z
167.4071
167.5167
207.5678
167.0548
168.1033
207.0907
149.0794
225.1372
109.0719
168.4902
287.0471
265.1679
127.0810
247.0295
413.1037
416.1249
169.0977
366.9581
290.0804
345.0844
368.9639
455.1538
OH
(S)
N
Br-
N+

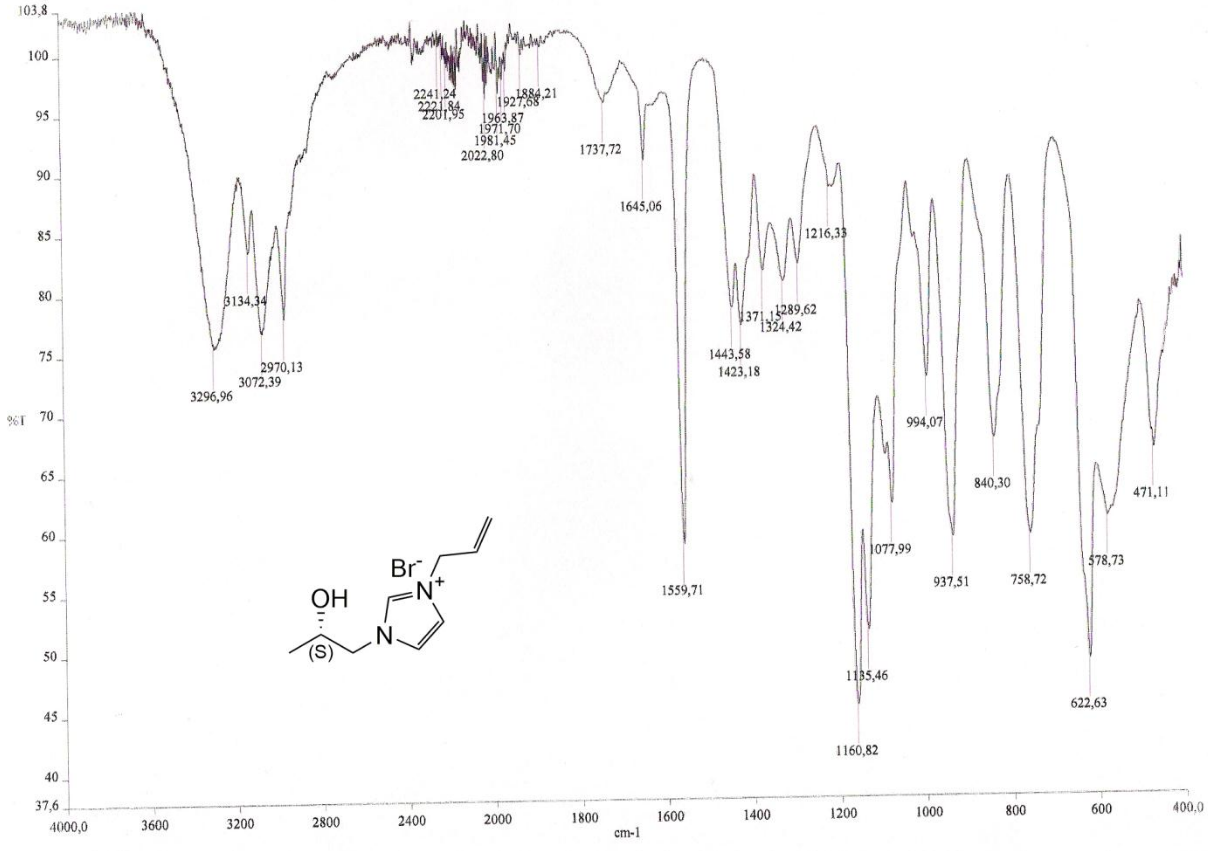

%T
cm-1
4000,0
400,0
3296,96
3134,34
3072,39
2970,13
2241,24
2221,84
2201,95
2022,80
1981,45
1971,70
1963,87
1927,68
1884,21
1737,72
1645,06
1559,71
1443,58
1423,18
1371,15
1324,42
1289,62
1216,33
1160,82
1135,46
1077,99
994,07
937,51
840,30
758,72
622,63
578,73
471,11
OH
(S)
N
Br-
N+

(*S*)-(+)-3-Butyl-1-(2-hydroxypropyl)-1*H*-imidazol-3-ium bromide (+)-7c:

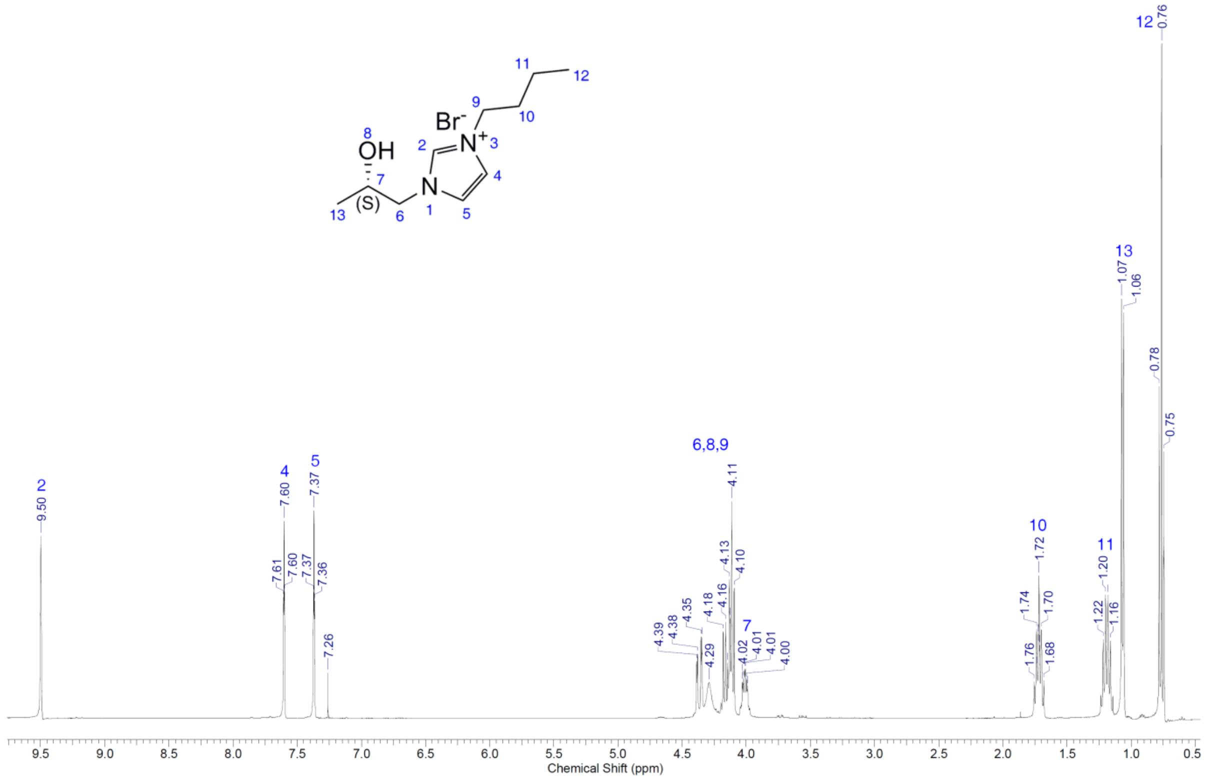

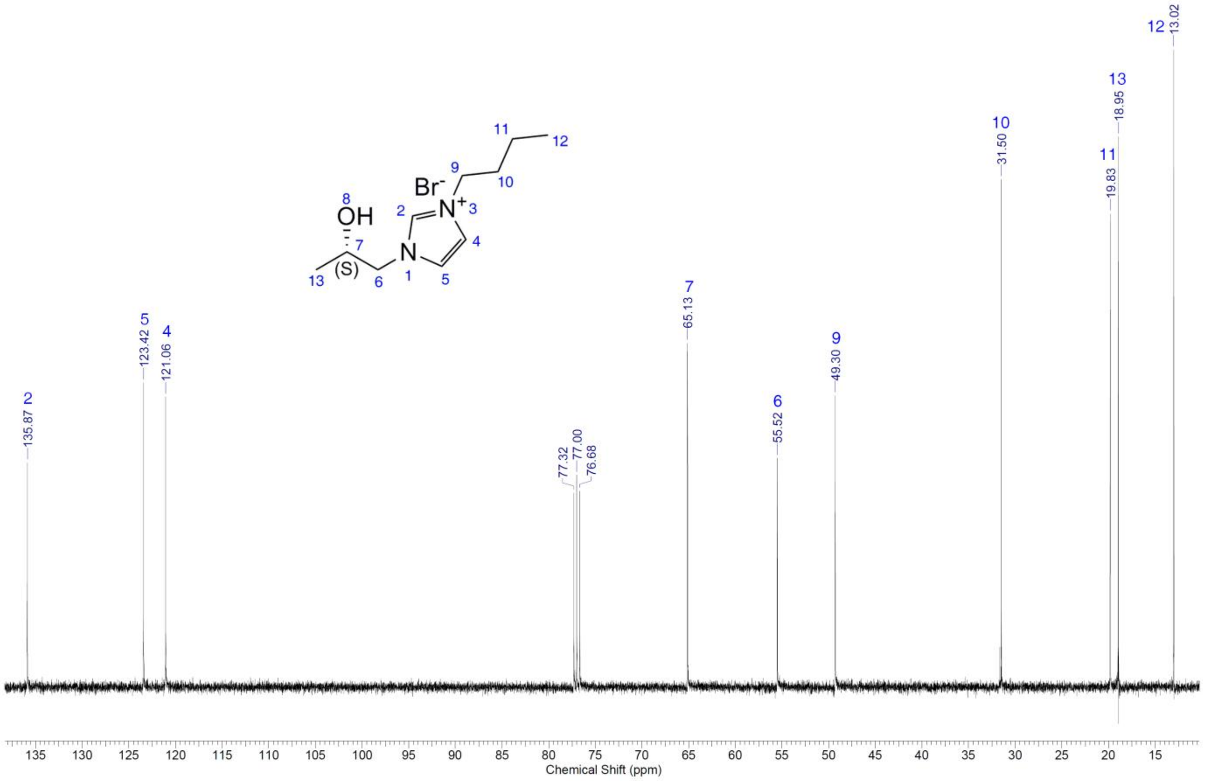

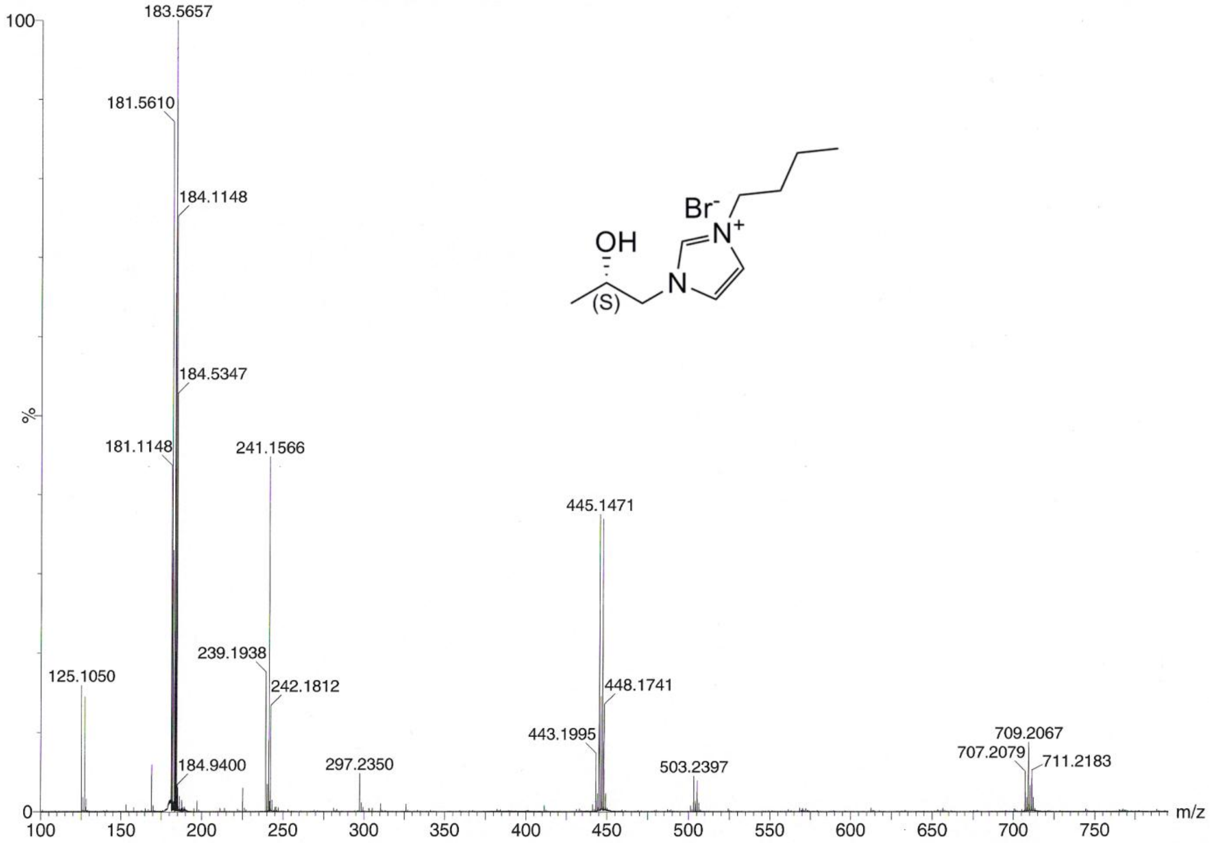
183.5657
181.5610
184.1148
184.5347
181.1148
241.1566
445.1471
125.1050
239.1938
242.1812
448.1741
443.1995
709.2067
707.2079
711.2183
184.9400
297.2350
503.2397
%
m/z
OH
(S)
Br⁻
N⁺
N

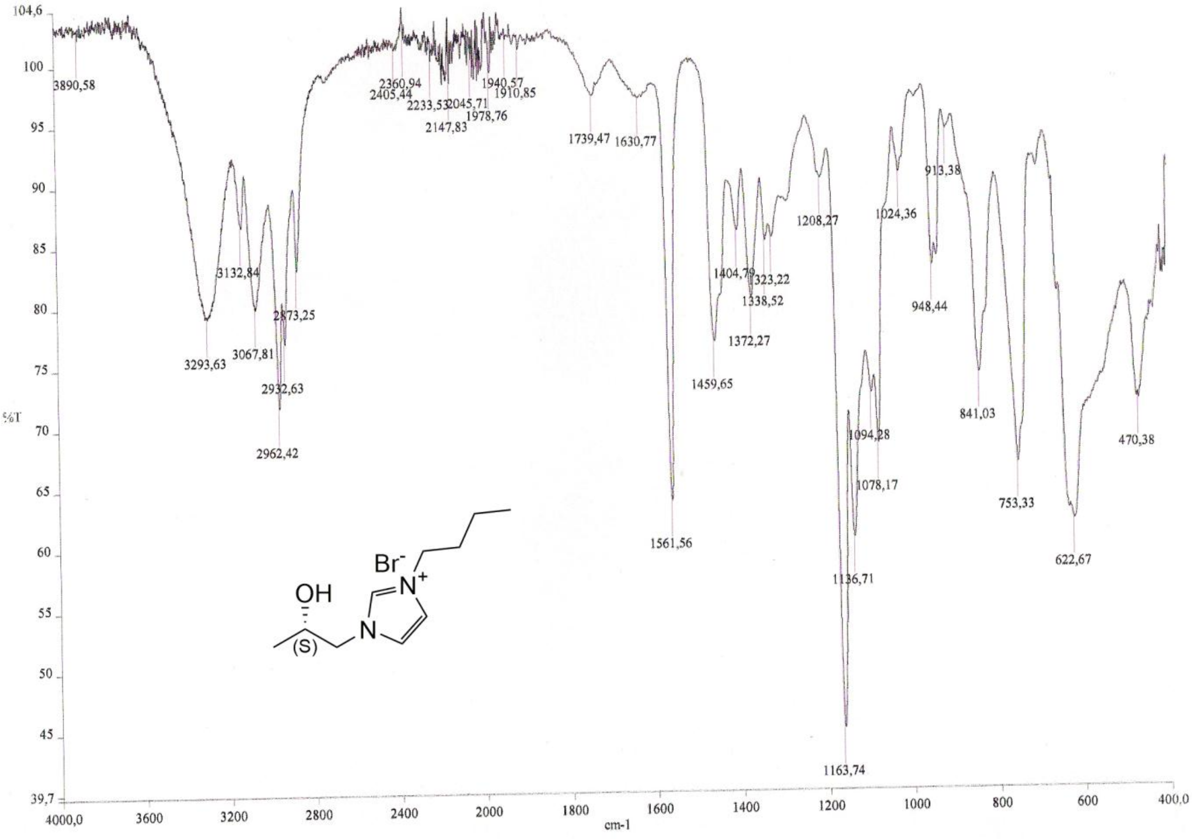
3890,58
2360,94
2405,44
2233,53
2045,71
2147,83
1978,76
1940,57
1910,85
1739,47
1630,77
3132,84
3293,63
3067,81
2873,25
2932,63
2962,42
1561,56
1459,65
1404,79
1323,22
1338,52
1372,27
1208,27
1094,28
1078,17
1136,71
1163,74
1024,36
913,38
948,44
841,03
753,33
622,67
470,38
%T
cm-1
OH
(S)
Br⁻
N⁺
N

(*S*)-(+)-1-(2-Hydroxypropyl)-3-pentyl-1*H*-imidazol-3-ium bromide (+)-7d:

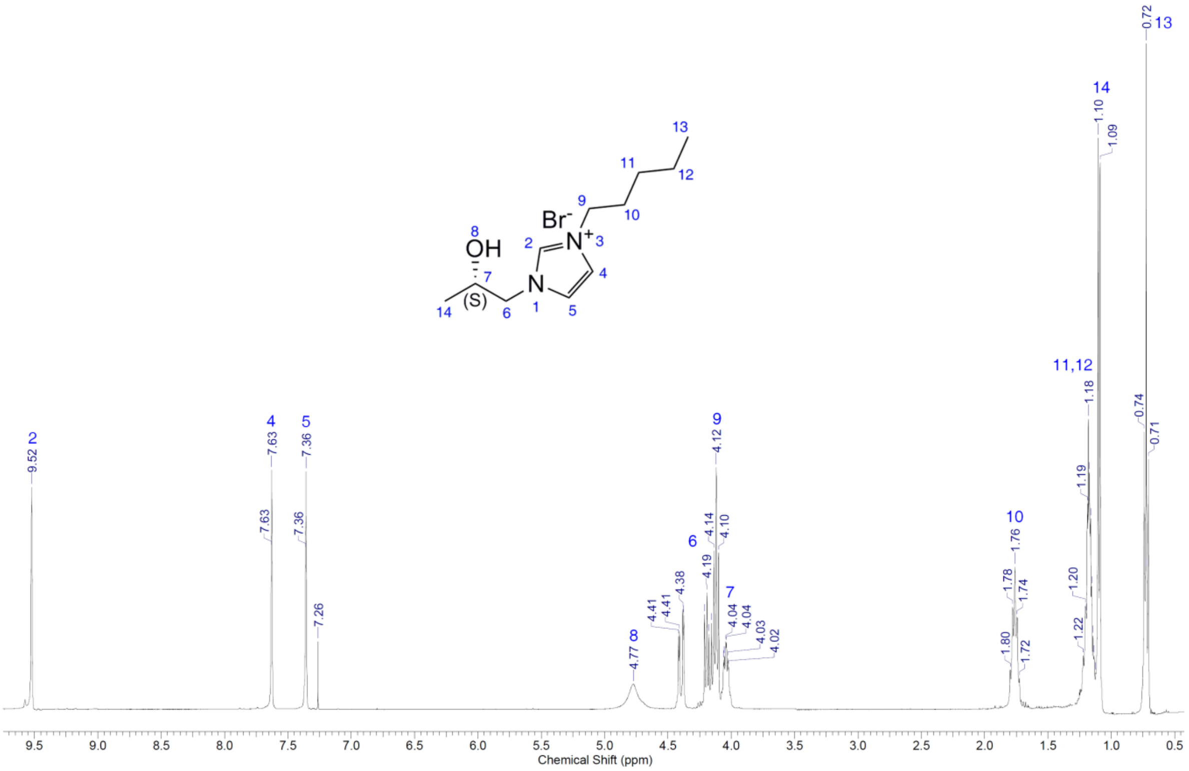

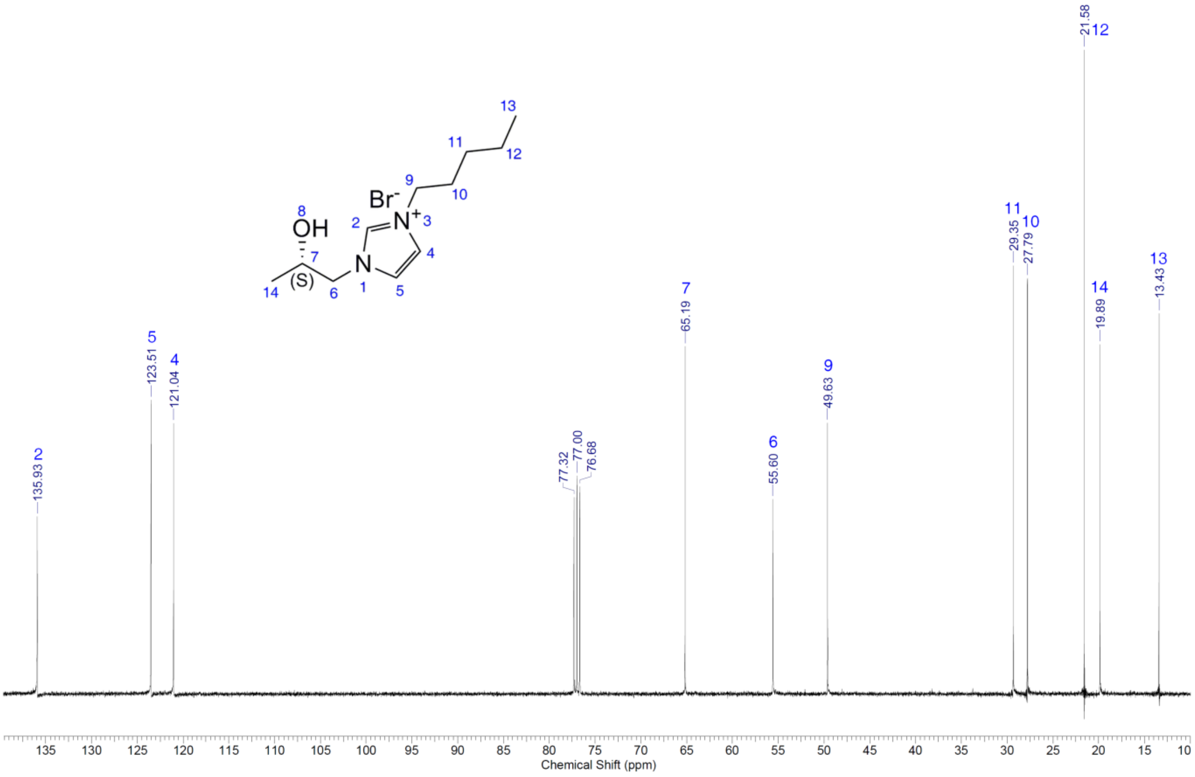

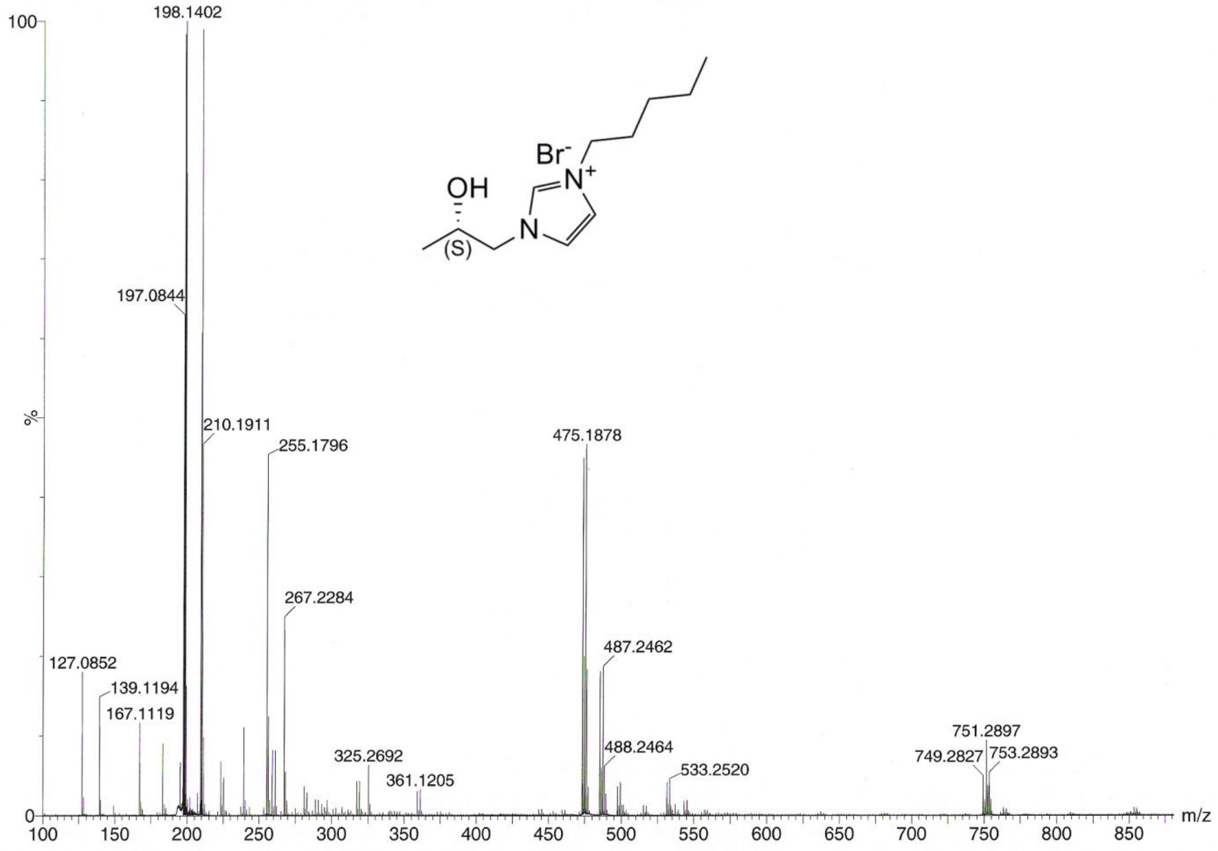

198.1402
197.0844
210.1911
255.1796
475.1878
267.2284
487.2462
127.0852
139.1194
167.1119
488.2464
325.2692
361.1205
533.2520
751.2897
749.2827
753.2893
%
m/z
Br⁻
OH
(S)
N
N⁺

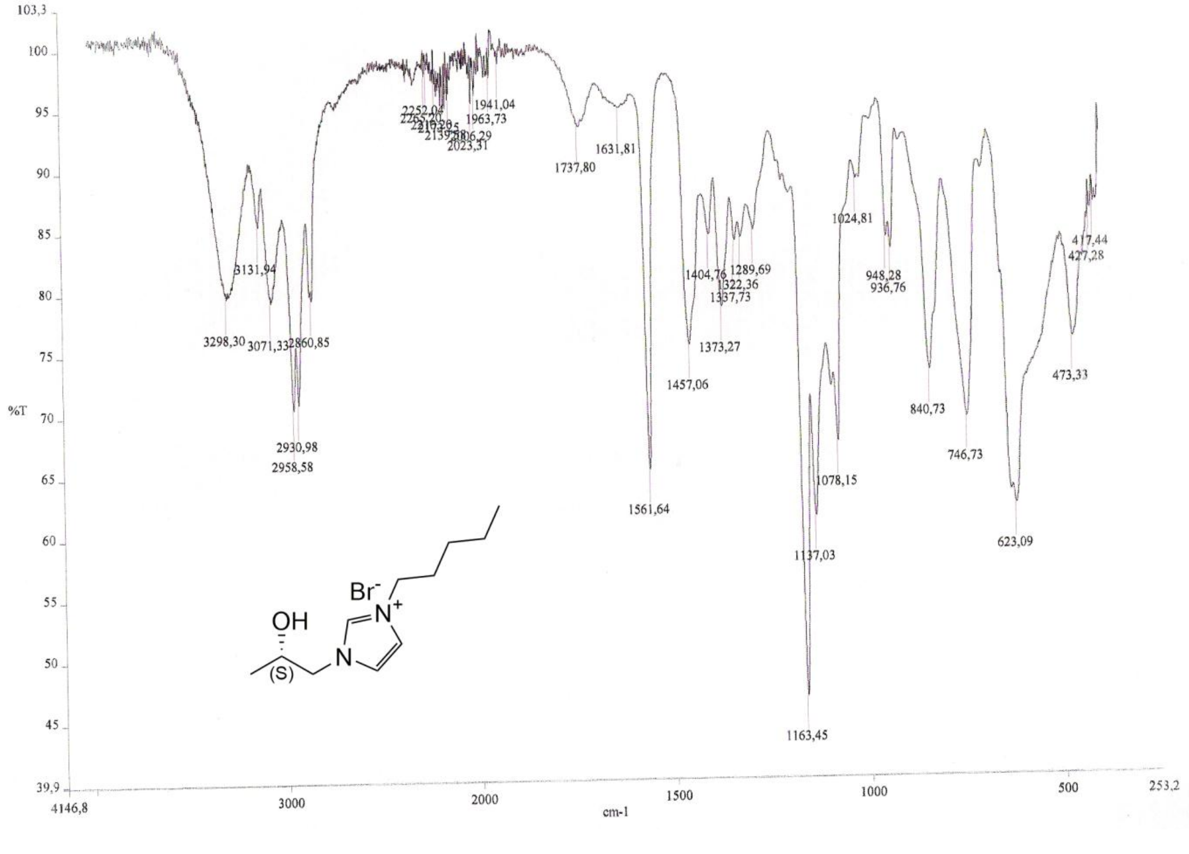

%T
cm-1
3298,30
3131,94
3071,33
2860,85
2930,98
2958,58
1941,04
1963,73
2023,31
1737,80
1631,81
1561,64
1457,06
1404,76
1373,27
1337,73
1322,36
1289,69
1163,45
1137,03
1078,15
1024,81
948,28
936,76
840,73
746,73
623,09
473,33
417,44
427,28
Br⁻
OH
(S)
N
N⁺

(*S*)-(+)-3-Heptyl-1-(2-hydroxypropyl)-1*H*-imidazol-3-ium iodide (+)-7e:

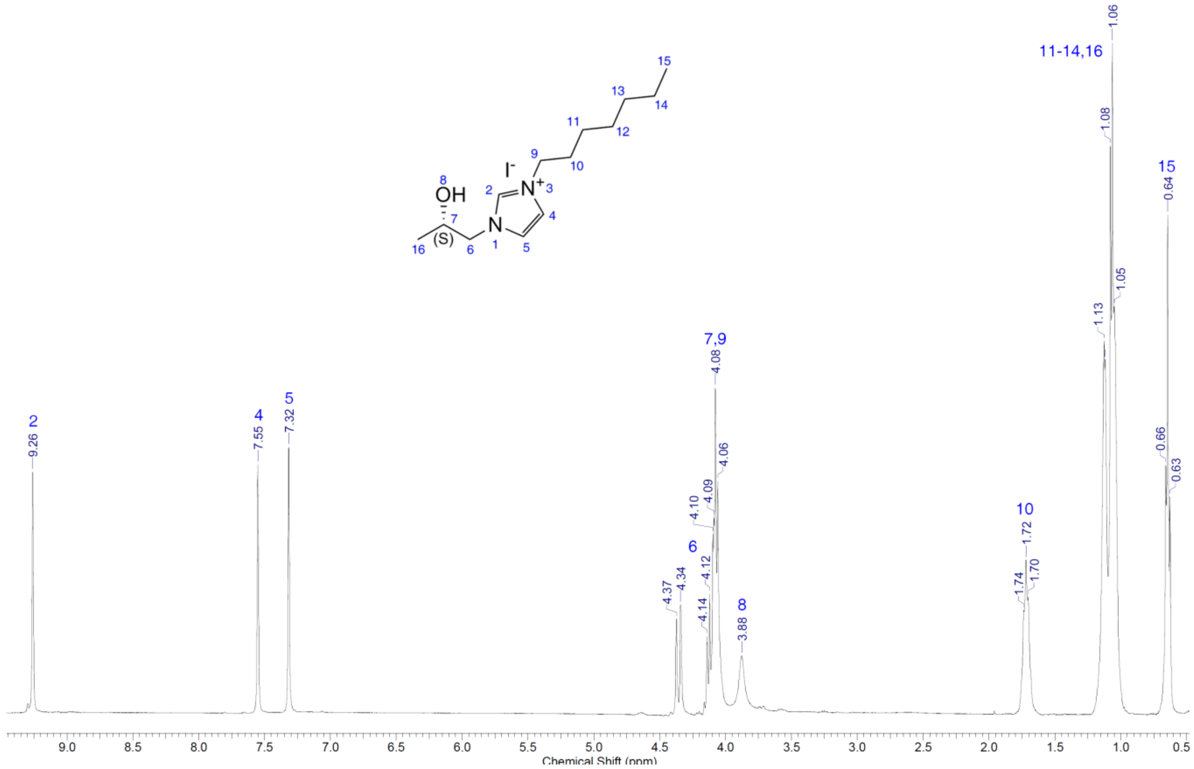

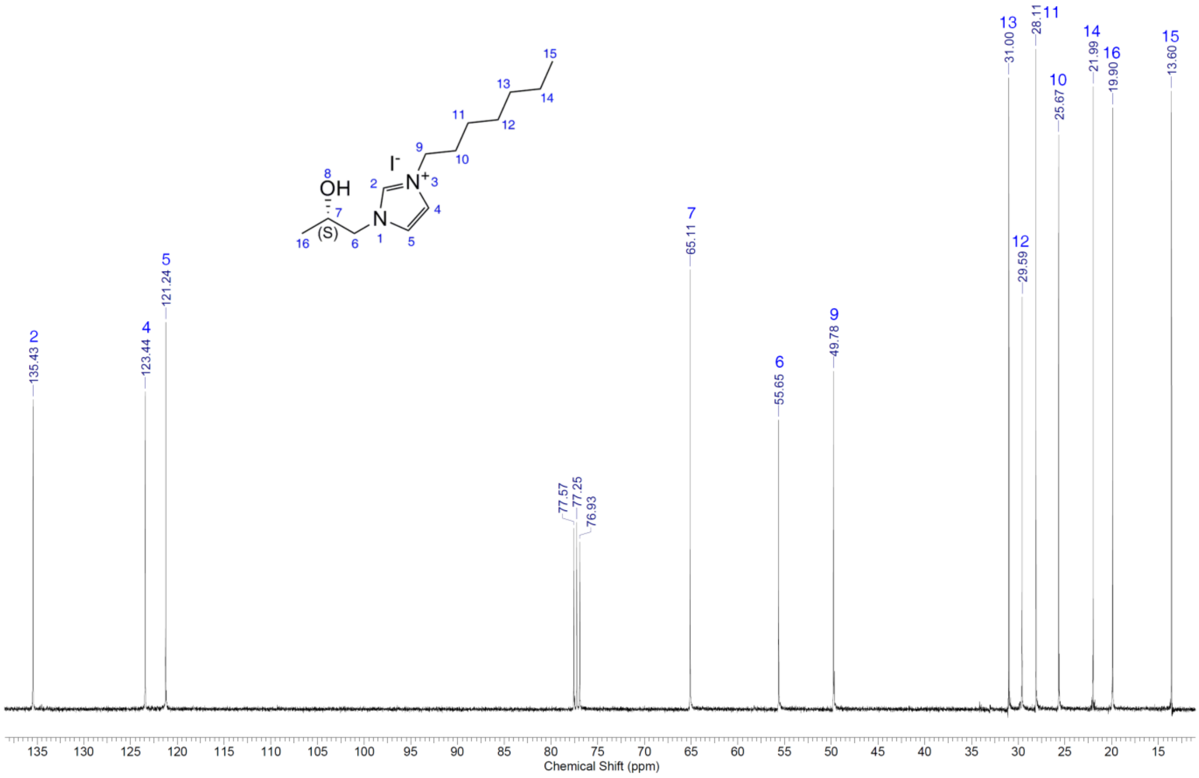

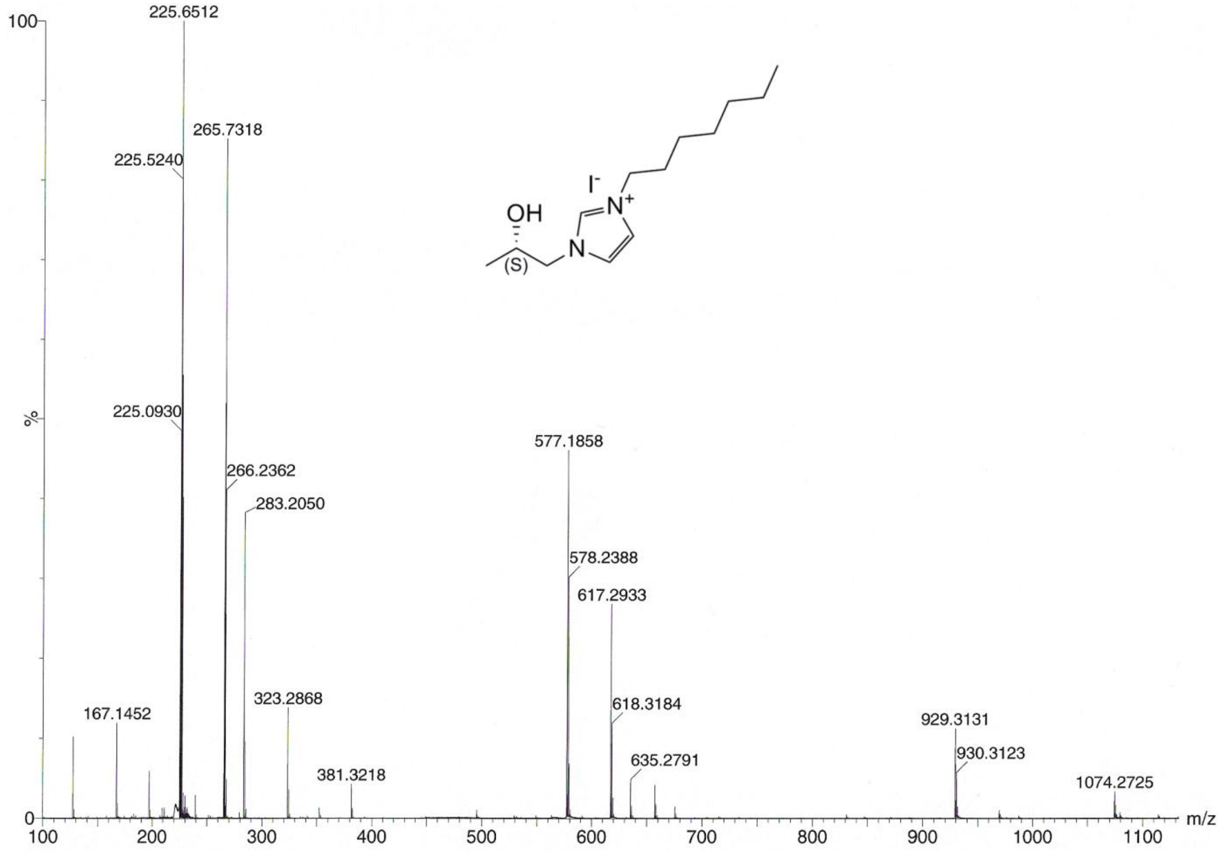
225.6512
265.7318
225.5240
225.0930
266.2362
283.2050
577.1858
578.2388
617.2933
618.3184
323.2868
167.1452
381.3218
635.2791
929.3131
930.3123
1074.2725
%
m/z
OH
(S)
N
N+
I-

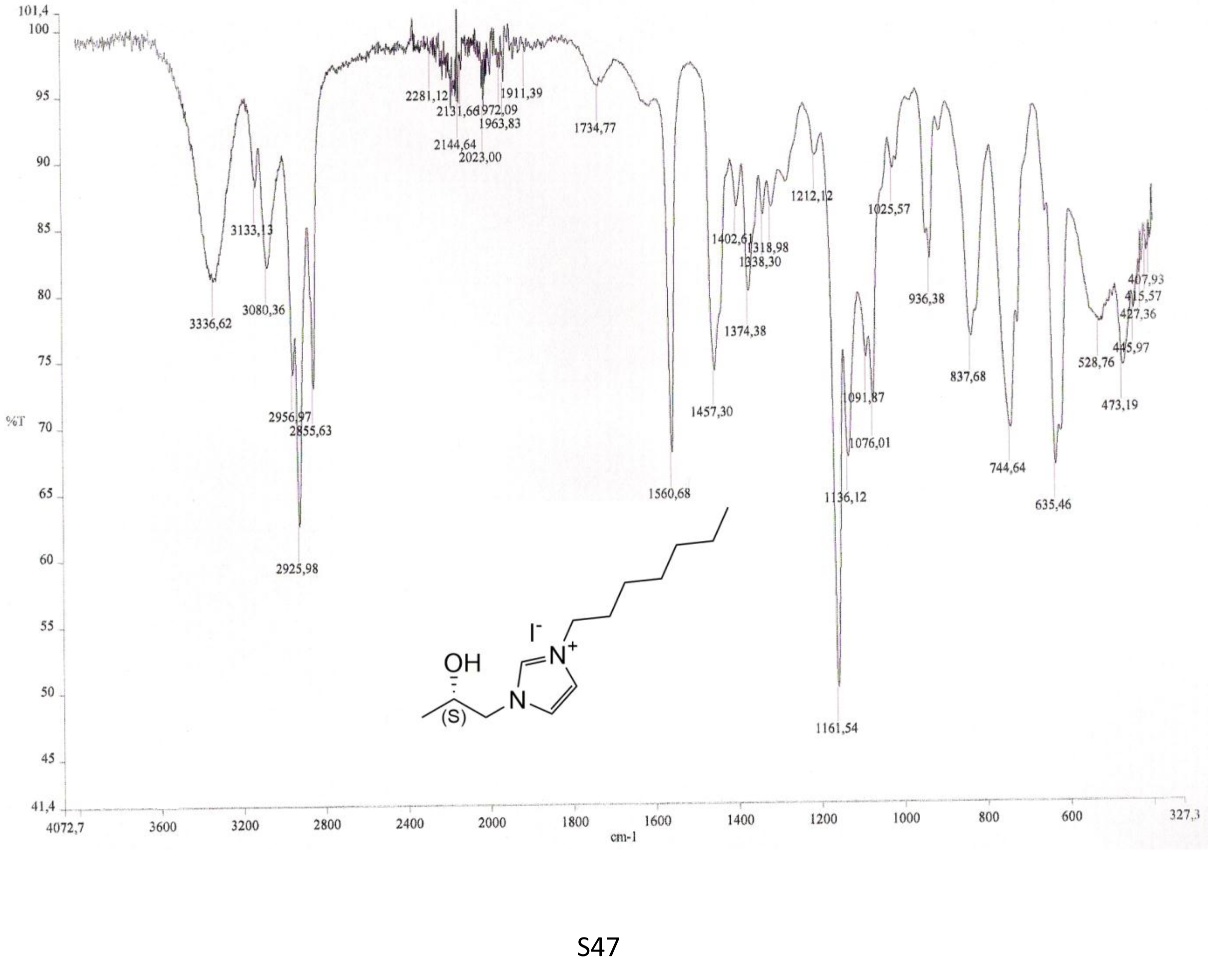
%T
cm-1
3336,62
3133,13
3080,36
2956,97
2925,98
2855,63
2281,12
2131,66
2144,64
1972,09
1963,83
2023,00
1911,39
1734,77
1560,68
1457,30
1402,61
1374,38
1338,30
1318,98
1212,12
1161,54
1136,12
1091,87
1076,01
1025,57
936,38
837,68
744,64
635,46
528,76
473,19
445,97
427,36
415,57
407,93
OH
(S)
N
N+
I-

(*S*)-(+)-3-Decyl-1-(2-hydroxypropyl)-1*H*-imidazol-3-ium iodide (+)-7f:

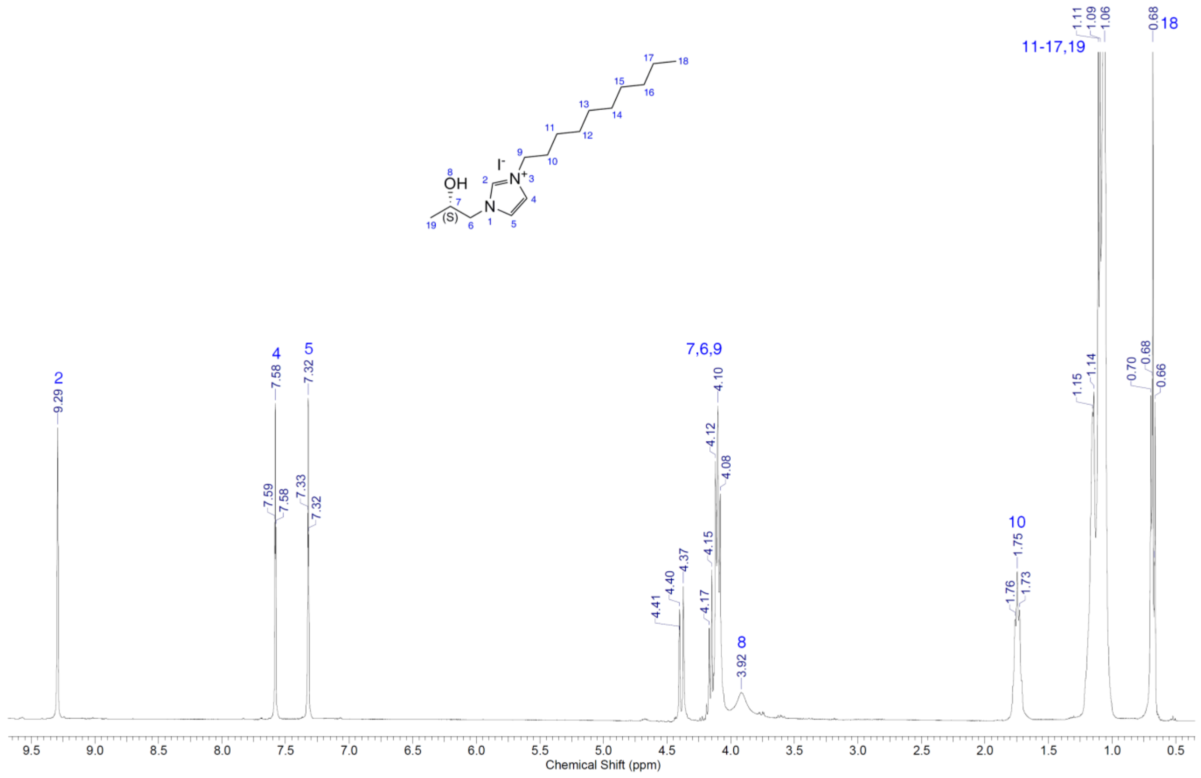

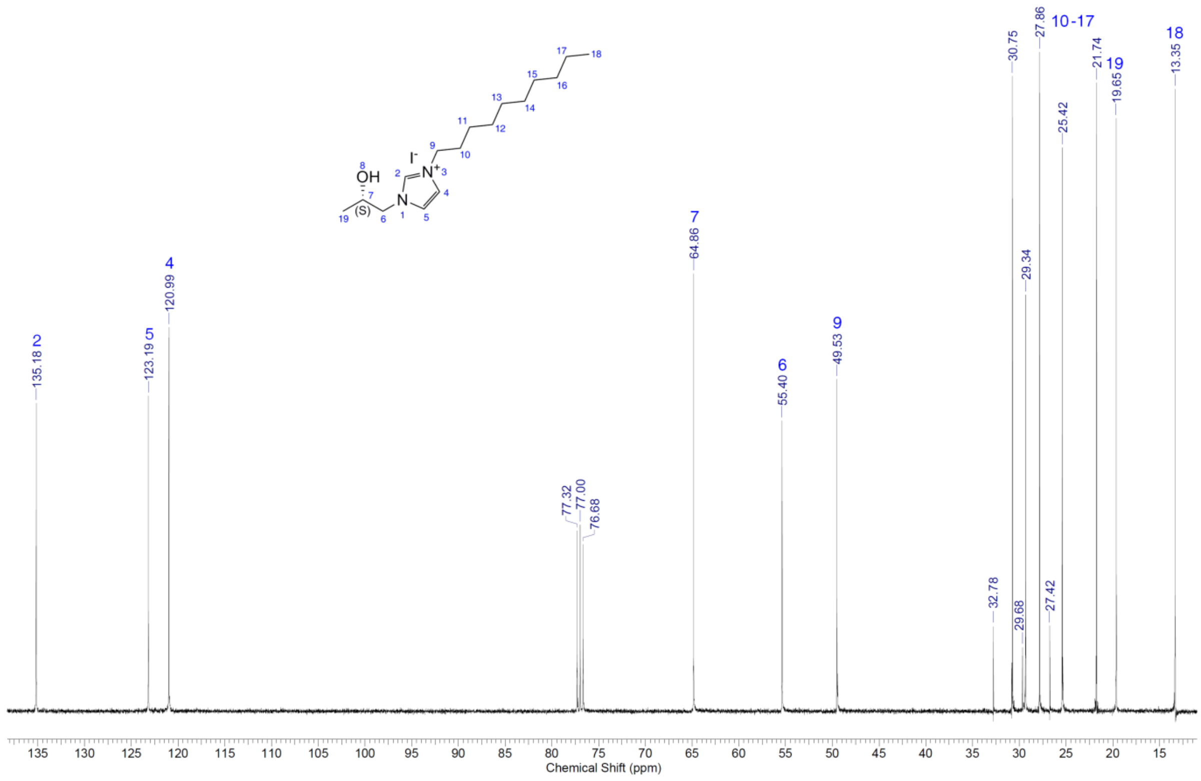

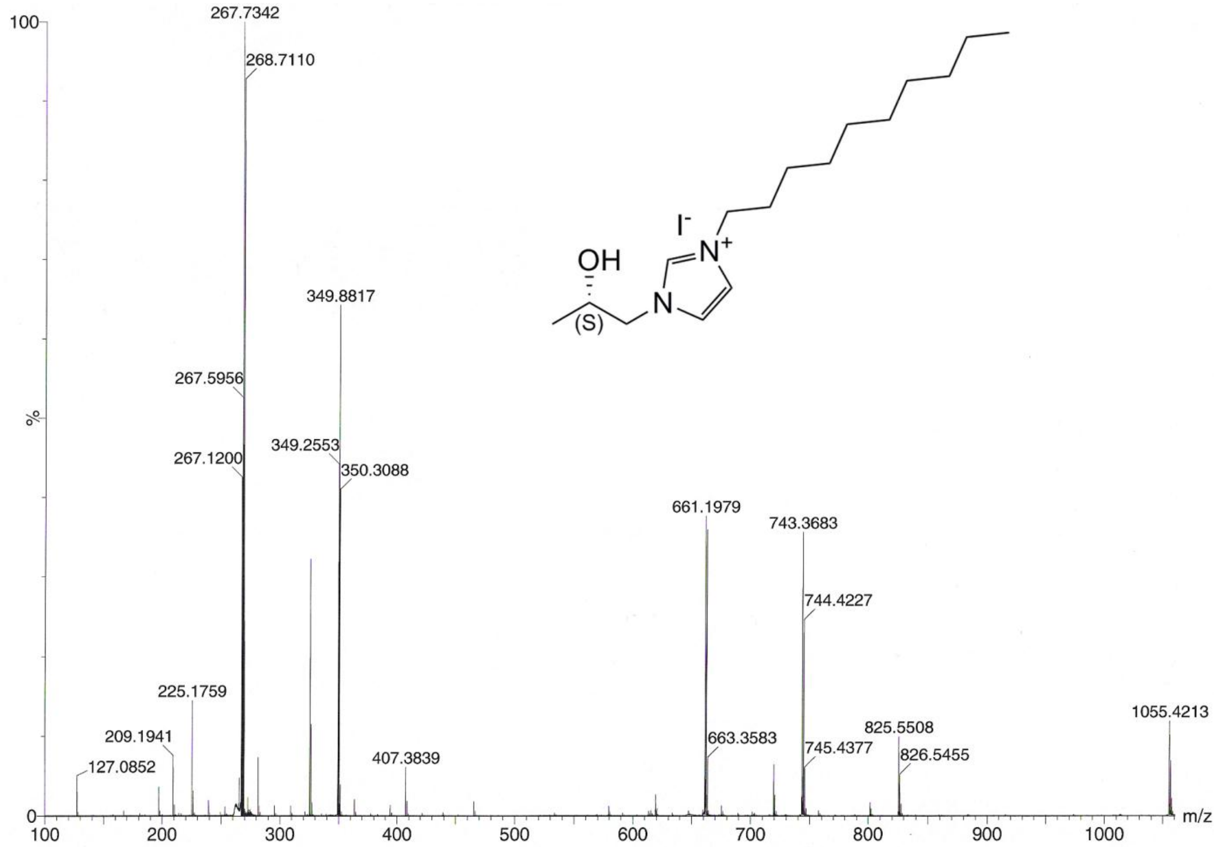
267.7342
268.7110
349.8817
267.5956
267.1200
349.2553
350.3088
661.1979
743.3683
744.4227
225.1759
209.1941
127.0852
407.3839
663.3583
745.4377
825.5508
826.5455
1055.4213
%
m/z
OH
(S)
I⁻
N
N+

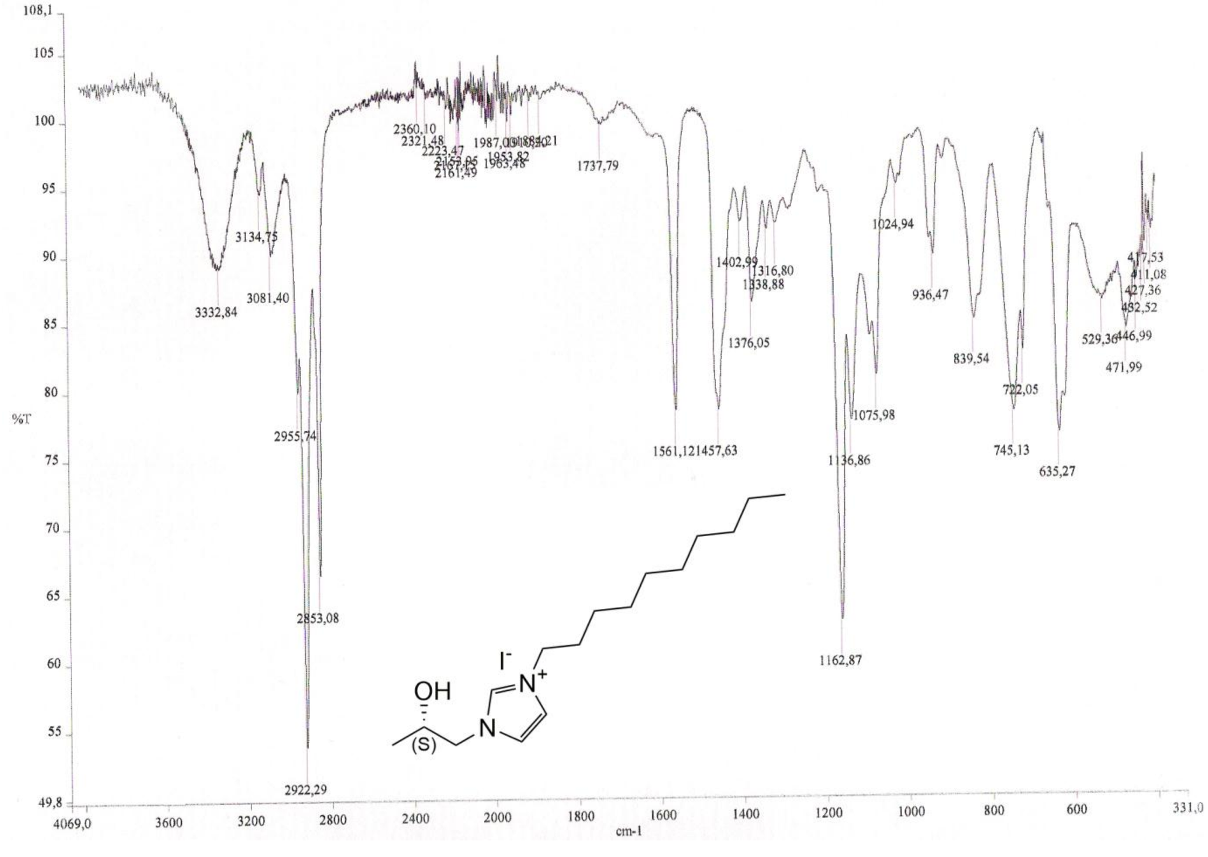
%T
cm-1
3332,84
3134,75
3081,40
2955,74
2922,29
2853,08
1737,79
1561,12
1457,63
1402,99
1376,05
1338,88
1316,80
1162,87
1136,86
1075,98
1024,94
936,47
839,54
745,13
722,05
635,27
529,36
471,99
446,99
OH
(S)
I⁻
N
N+

(*S*)-(+)-3-Dodecyl-1-(2-hydroxypropyl)-1*H*-imidazol-3-ium iodide (+)-7g:

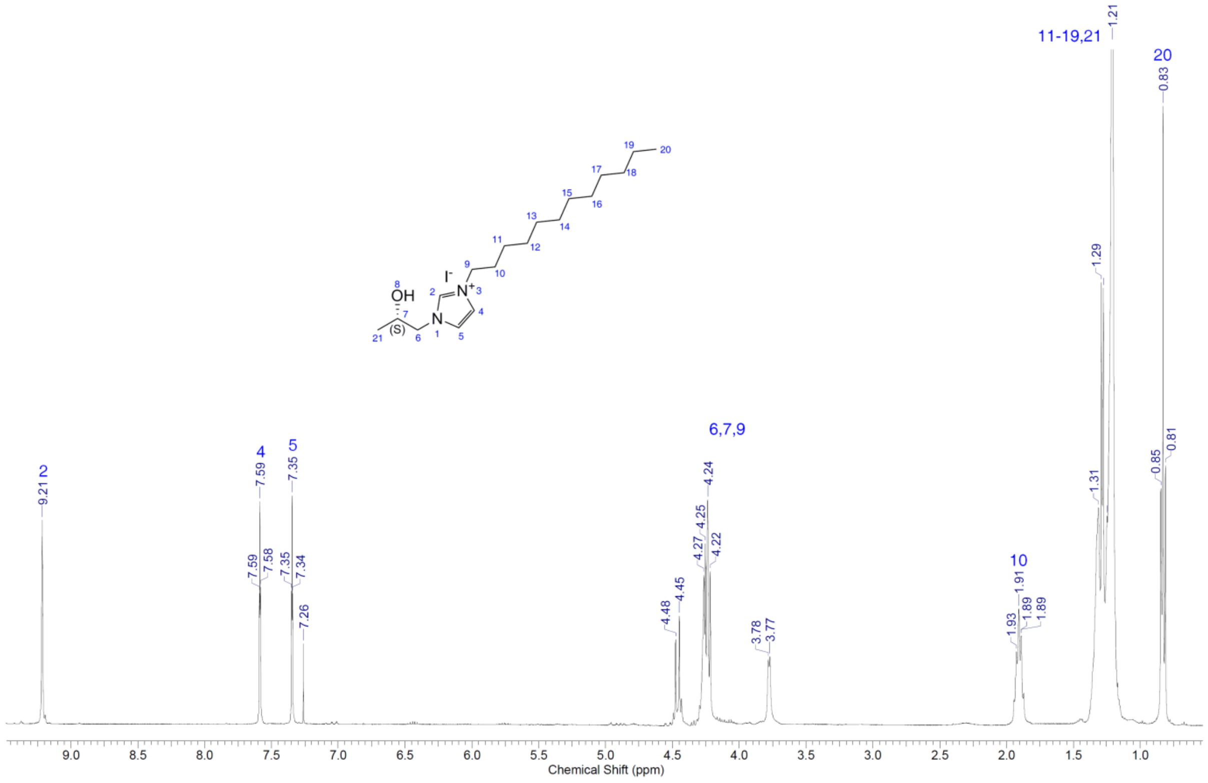

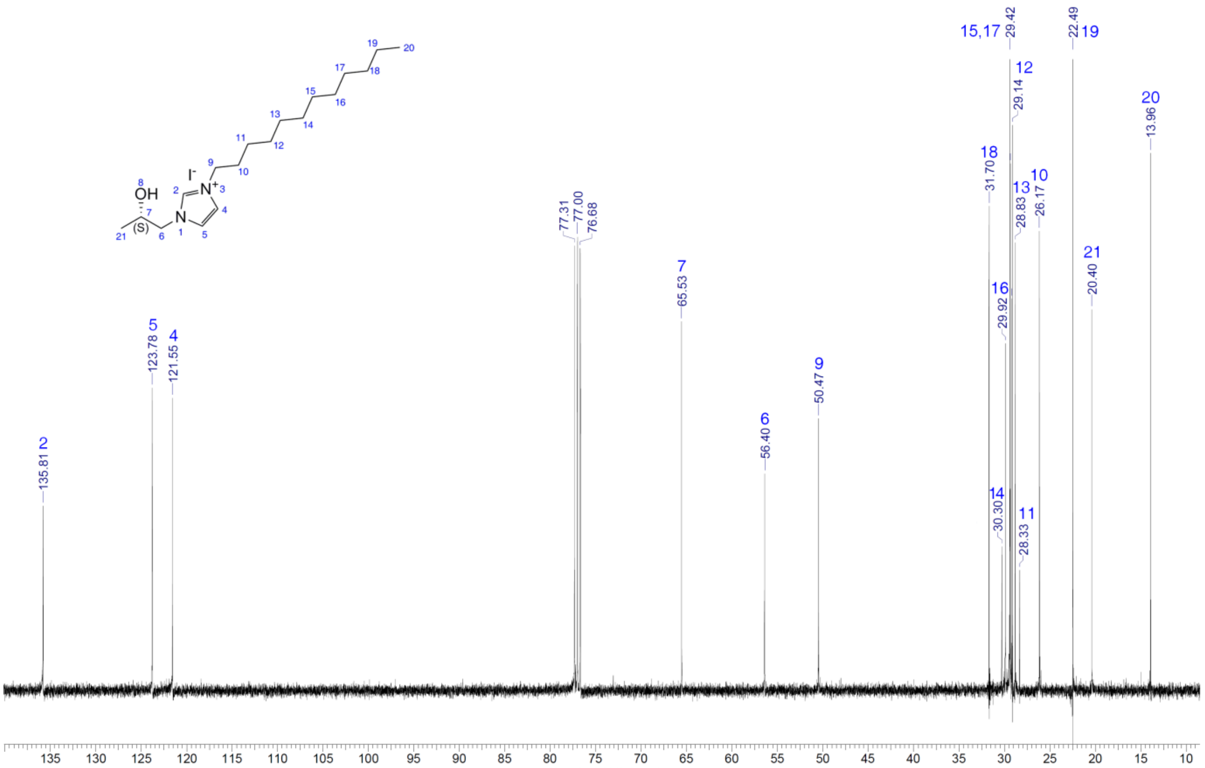

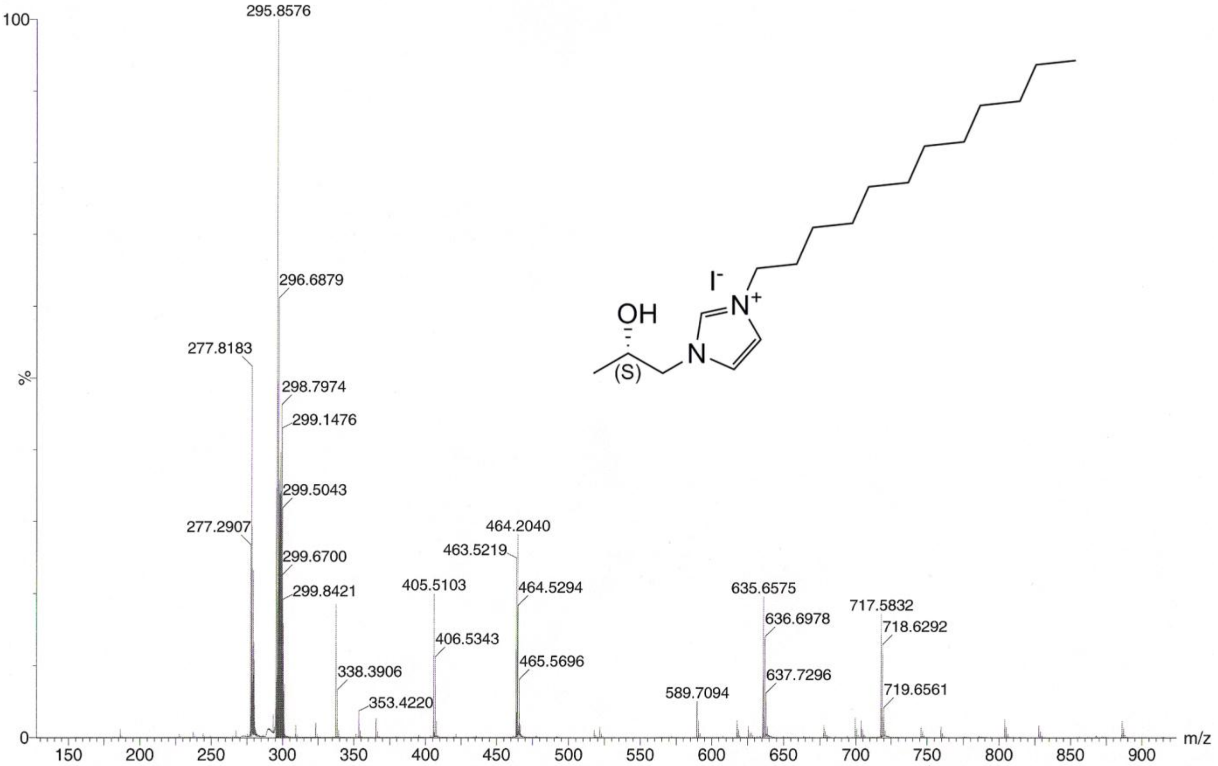
295.8576
296.6879
277.8183
298.7974
299.1476
299.5043
277.2907
464.2040
463.5219
299.6700
299.8421
405.5103
464.5294
635.6575
717.5832
636.6978
718.6292
406.5343
465.5696
338.3906
589.7094
637.7296
719.6561
353.4220
%
m/z
I⁻
OH
(S)
N
N⁺

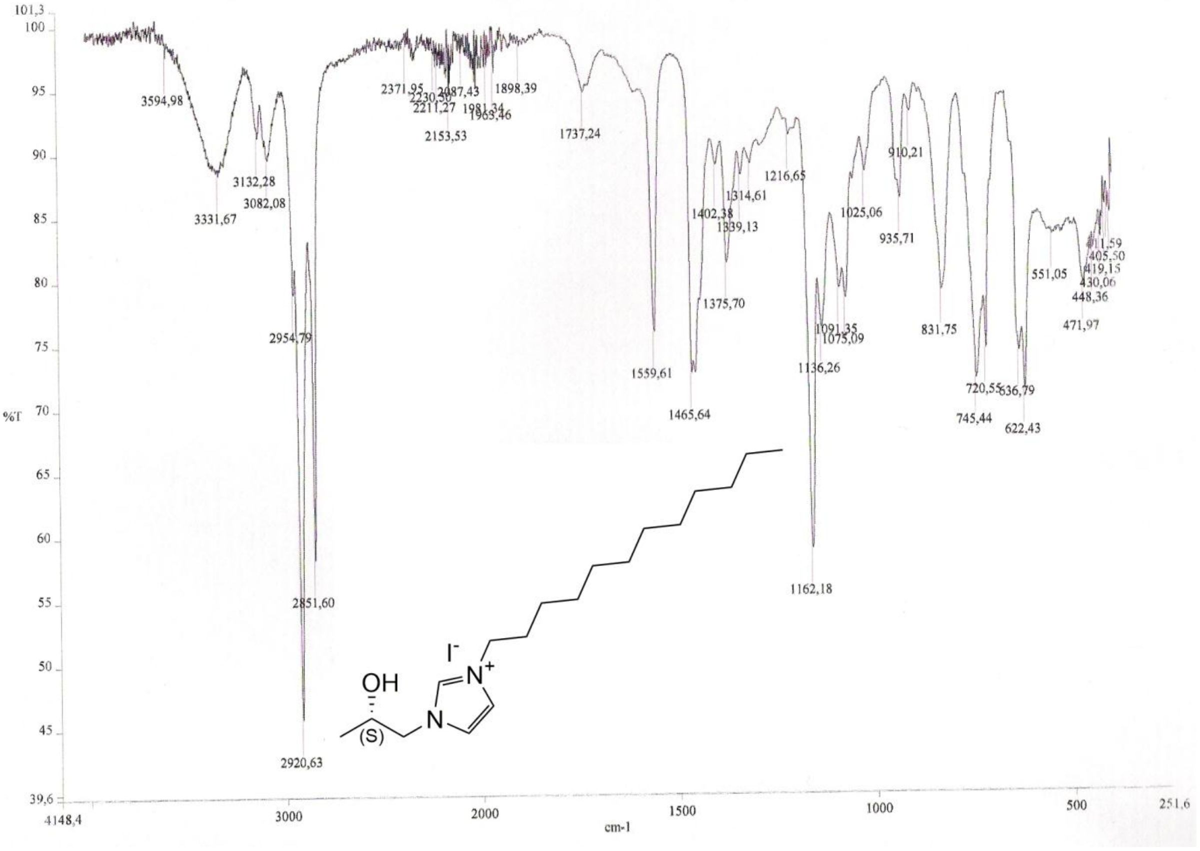
%T
cm-1
3594,98
3331,67
3132,28
3082,08
2954,79
2920,63
2851,60
2371,95
2153,53
1898,39
1737,24
1559,61
1465,64
1402,38
1375,70
1339,13
1314,61
1216,65
1162,18
1136,26
1091,35
1075,09
1025,06
935,71
910,21
831,75
745,44
720,55
636,79
622,43
551,05
471,97
448,36
430,06
419,15
405,50
I⁻
OH
(S)
N
N⁺

(*S*)-(+)-3-Hexadecyl-1-(2-hydroxypropyl)-1*H*-imidazol-3-ium iodide (+)-7h:

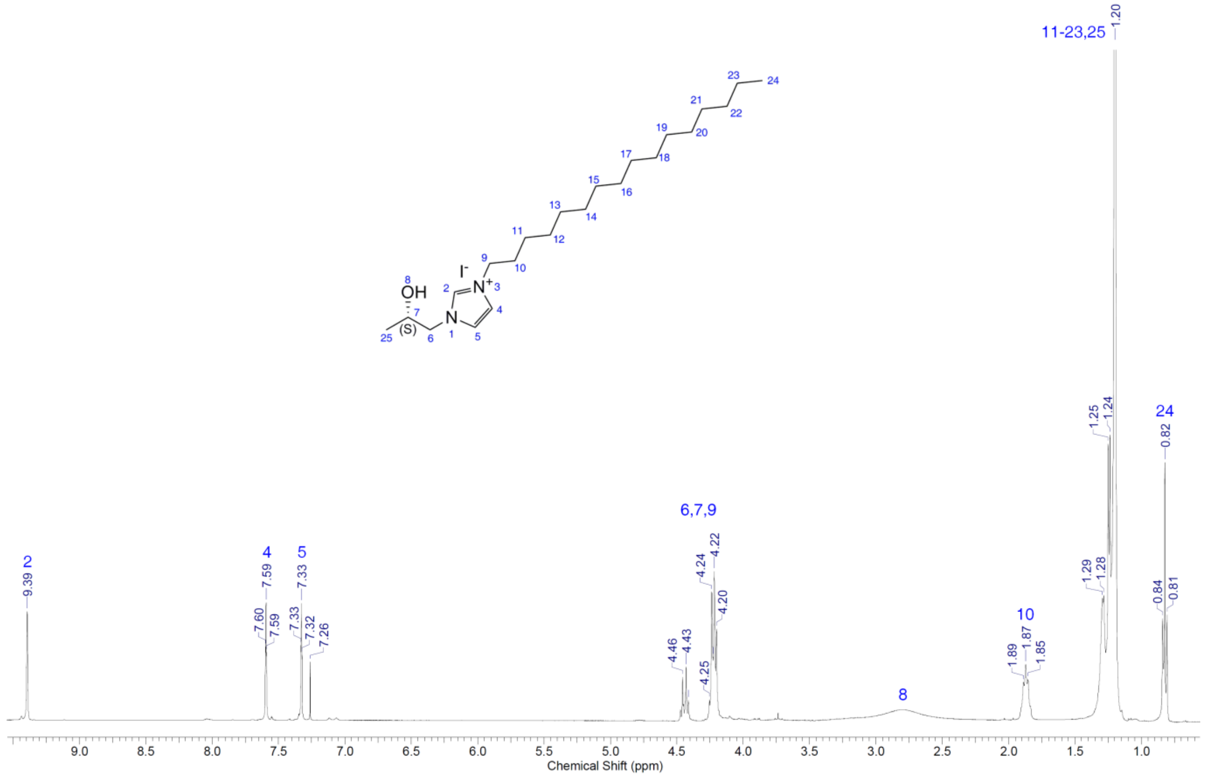

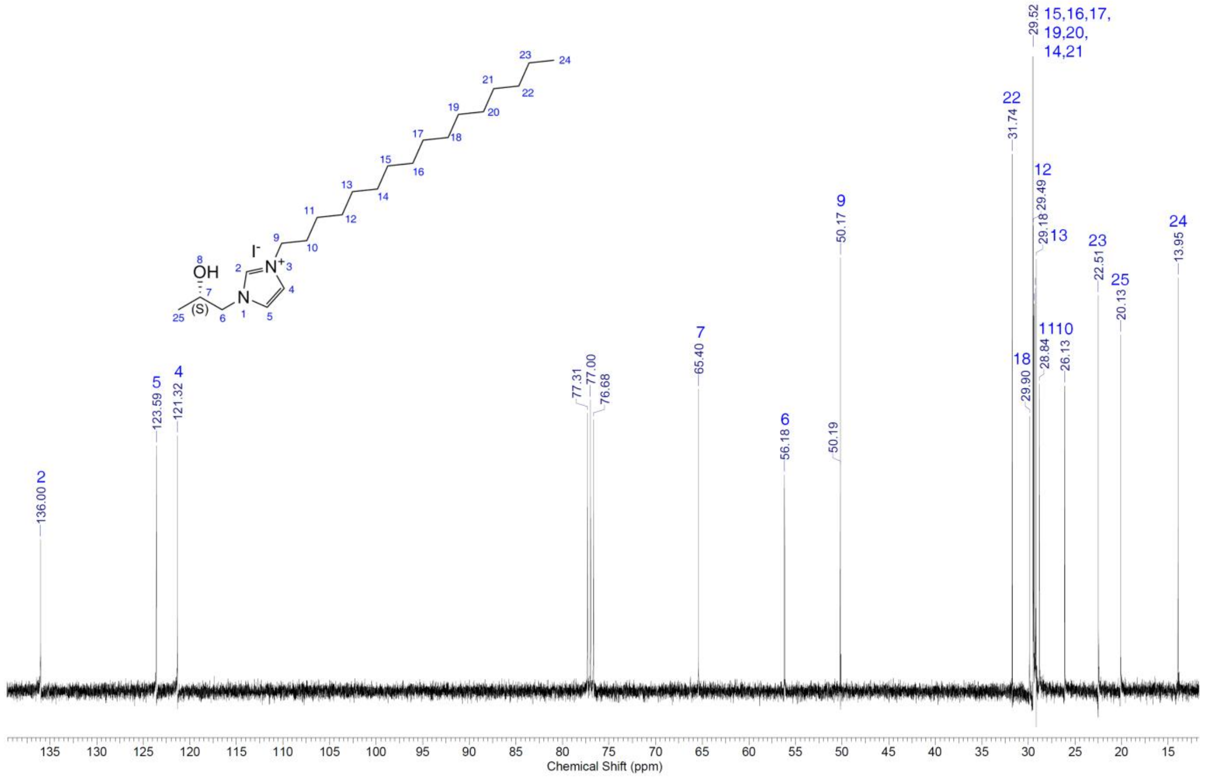

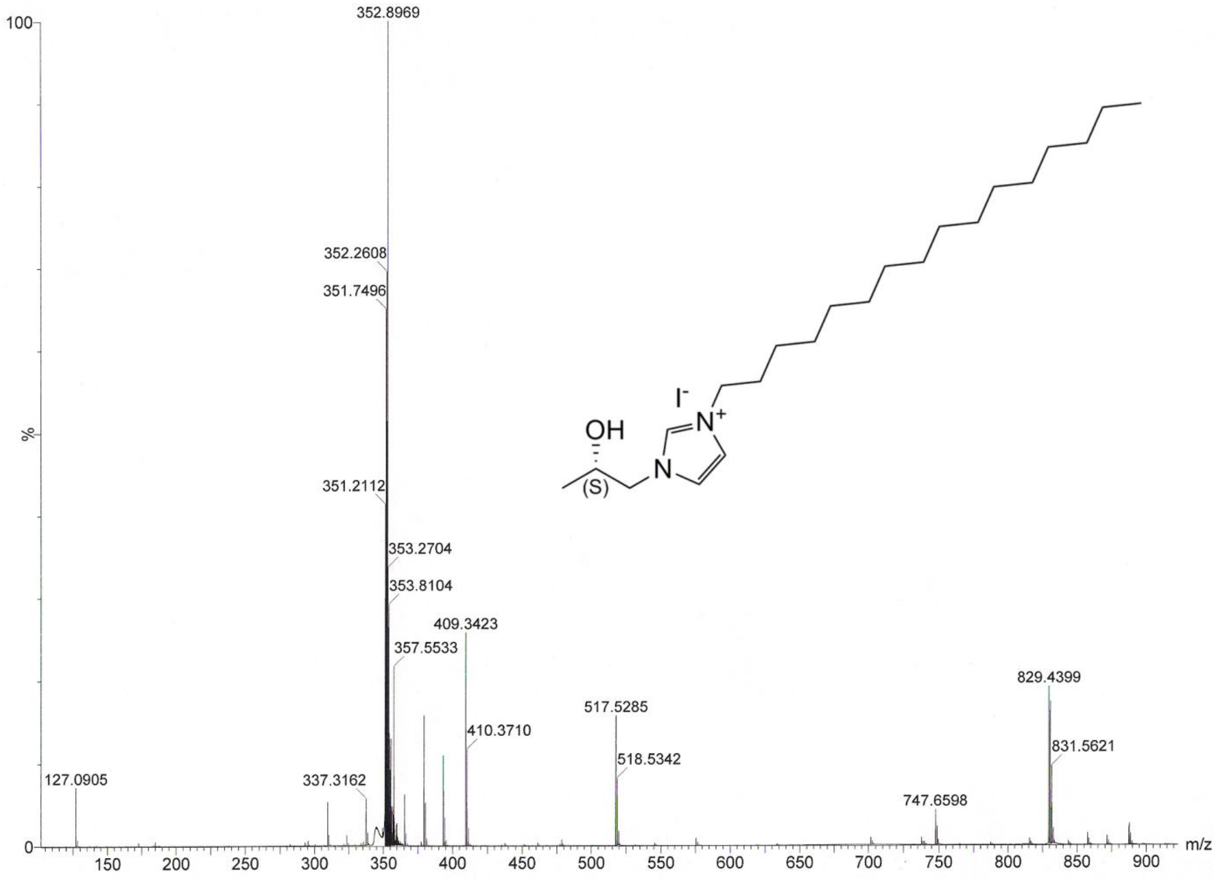
352.8969
352.2608
351.7496
351.2112
353.2704
353.8104
409.3423
357.5533
829.4399
517.5285
410.3710
831.5621
518.5342
127.0905
337.3162
747.6598
OH
I-
N+
N
(S)
m/z
%

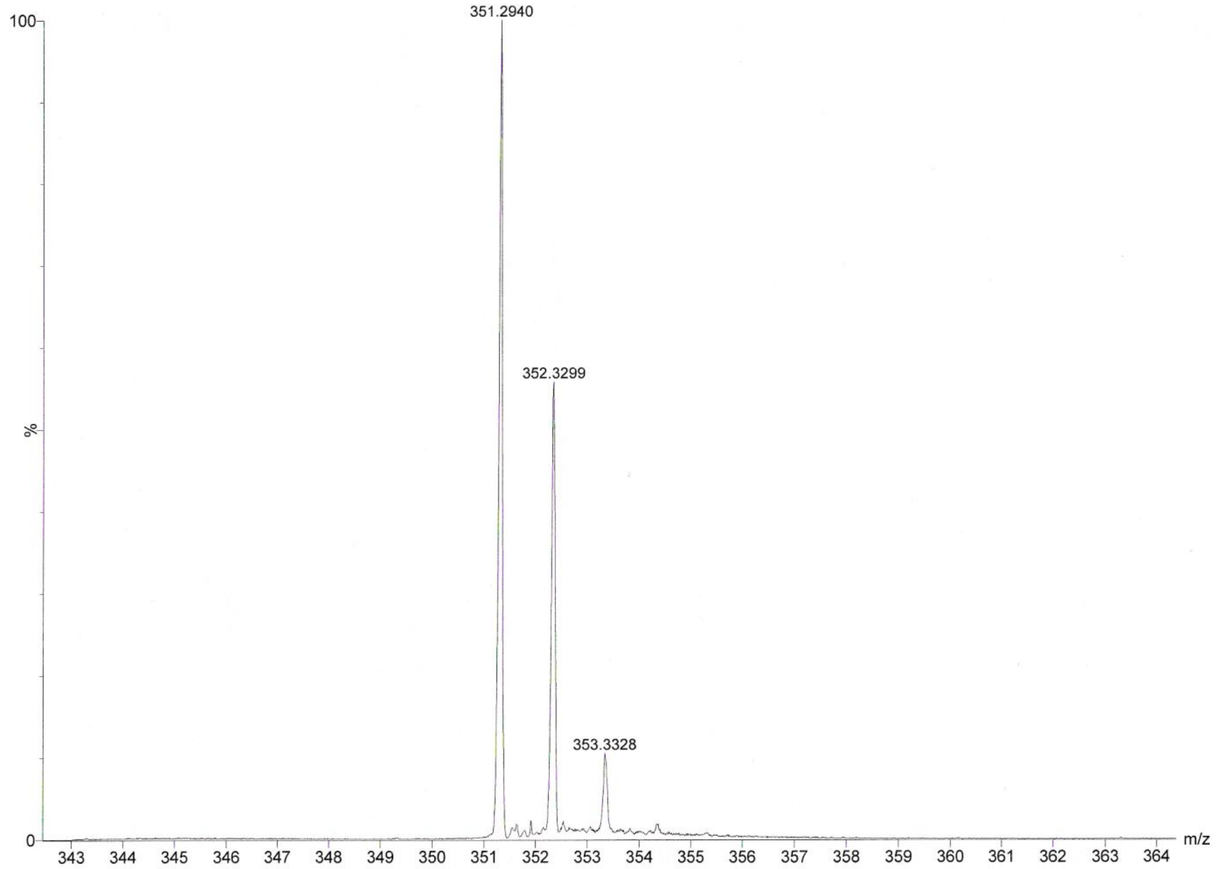
351.2940
352.3299
353.3328
m/z
%

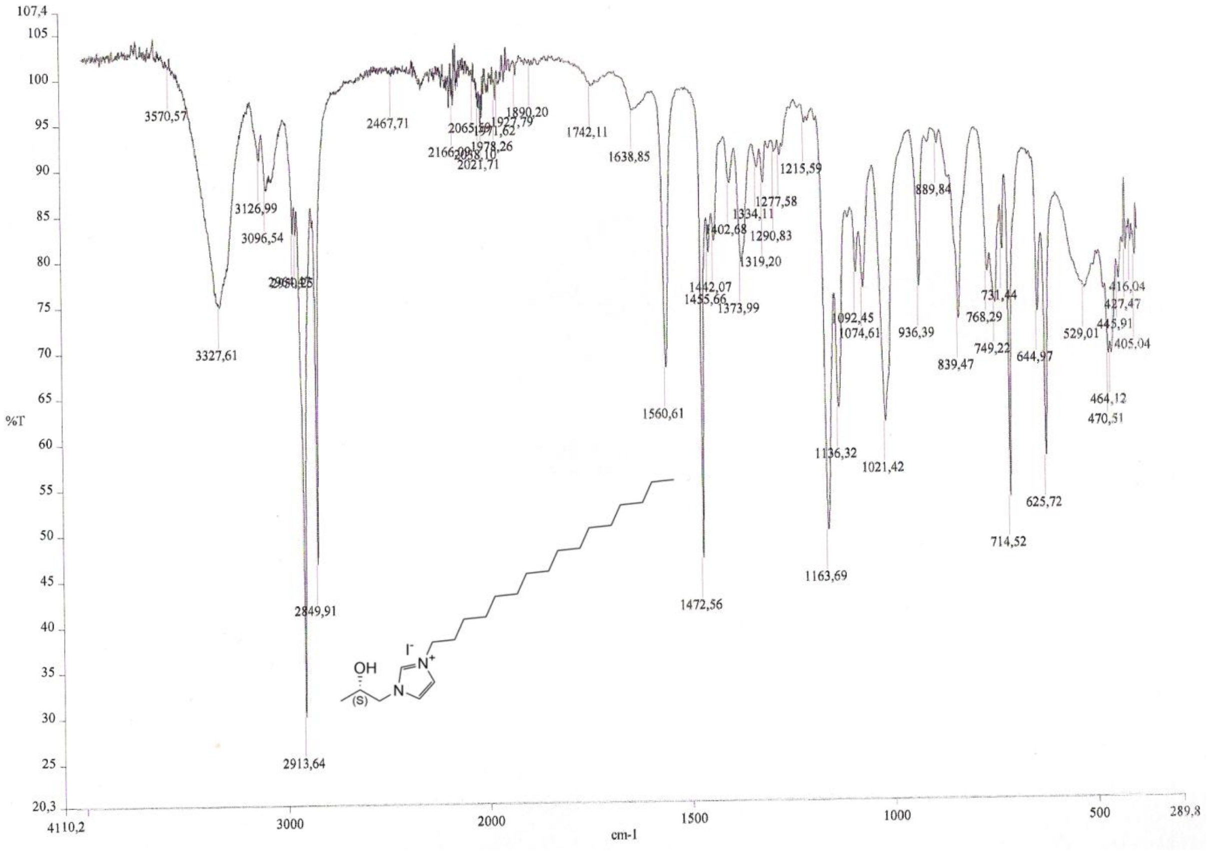

3570,57
3327,61
3126,99
3096,54
2913,64
2849,91
2467,71
2065,59
2166,09
2021,71
1978,26
1927,79
1890,20
1742,11
1638,85
1560,61
1472,56
1455,66
1442,07
1402,68
1373,99
1334,11
1319,20
1290,83
1277,58
1215,59
1163,69
1136,32
1092,45
1074,61
1021,42
936,39
889,84
839,47
768,29
749,22
731,44
714,52
644,97
625,72
529,01
470,51
464,12
445,91
427,47
416,04
405,04
%T
cm-1
4110,2
289,8
107,4
20,3
OH
(S)
N
N+
I-

Spectra of the triazolium salts [(+)-8a–f]

(*S*)-(+)-1-(2-Hydroxypropyl)-4-propyl-1*H*-1,2,4-triazol-4-ium bromide (+)-8a:

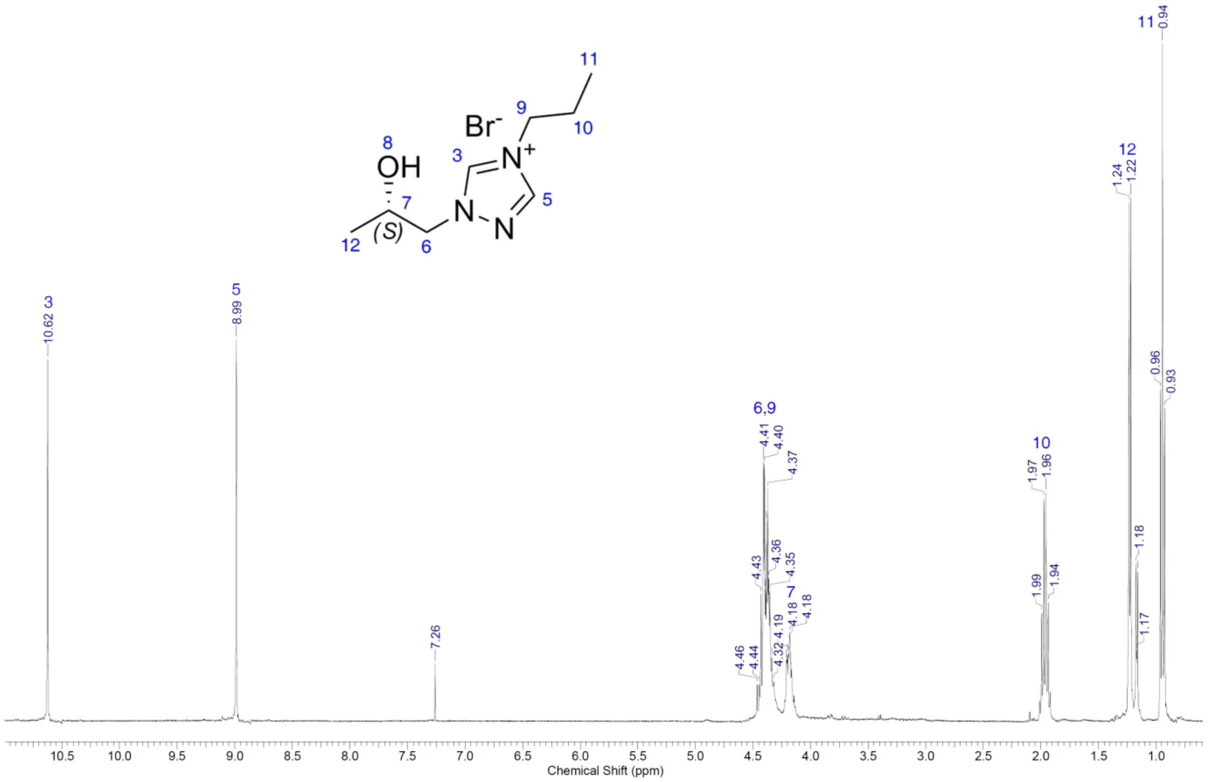

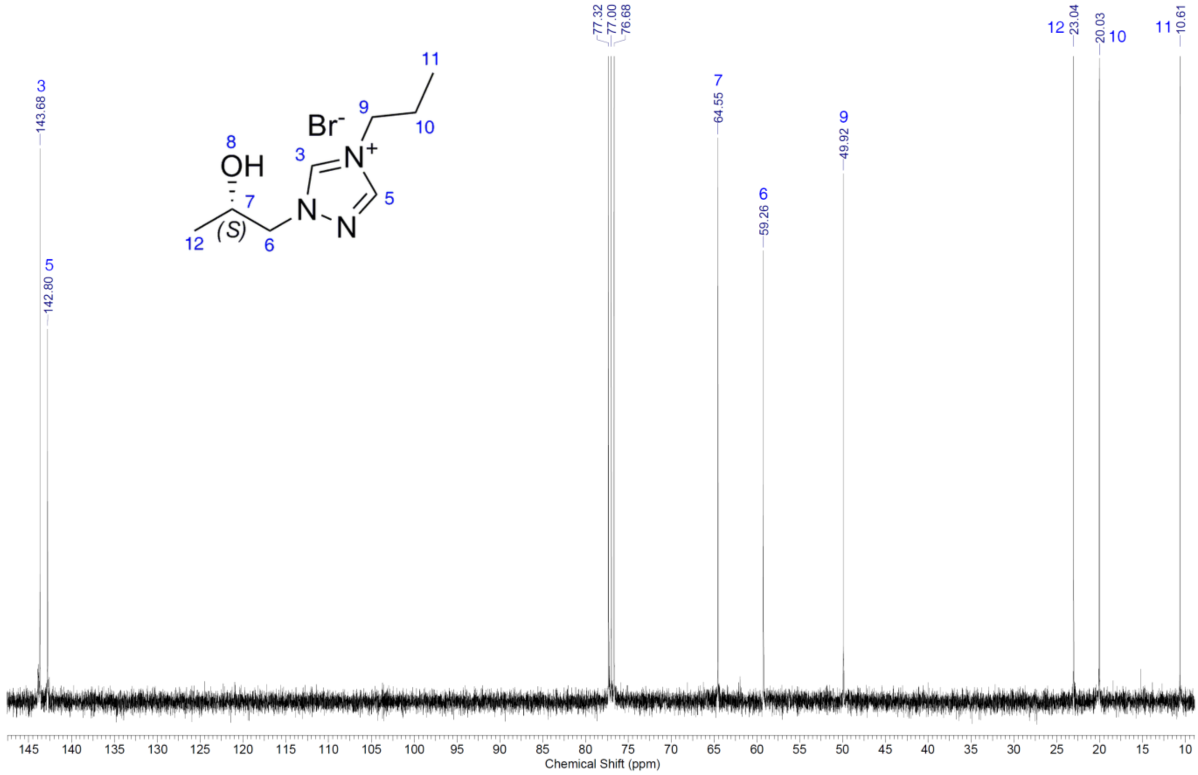

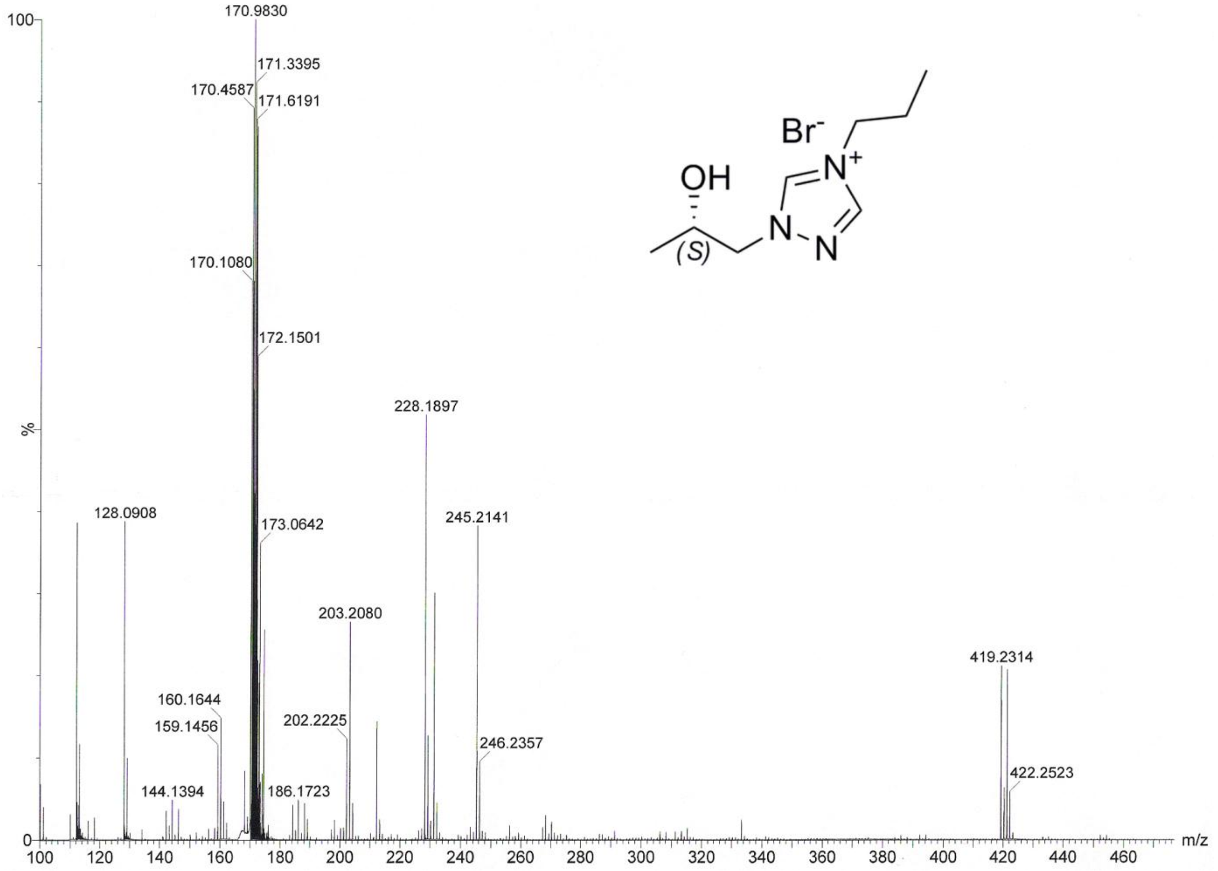
100
170.9830
171.3395
170.4587
171.6191
170.1080
172.1501
228.1897
%
128.0908
173.0642
245.2141
203.2080
419.2314
160.1644
159.1456
202.2225
246.2357
422.2523
144.1394
186.1723
0
m/z
Br⁻
OH
(S)
N
N
N⁺

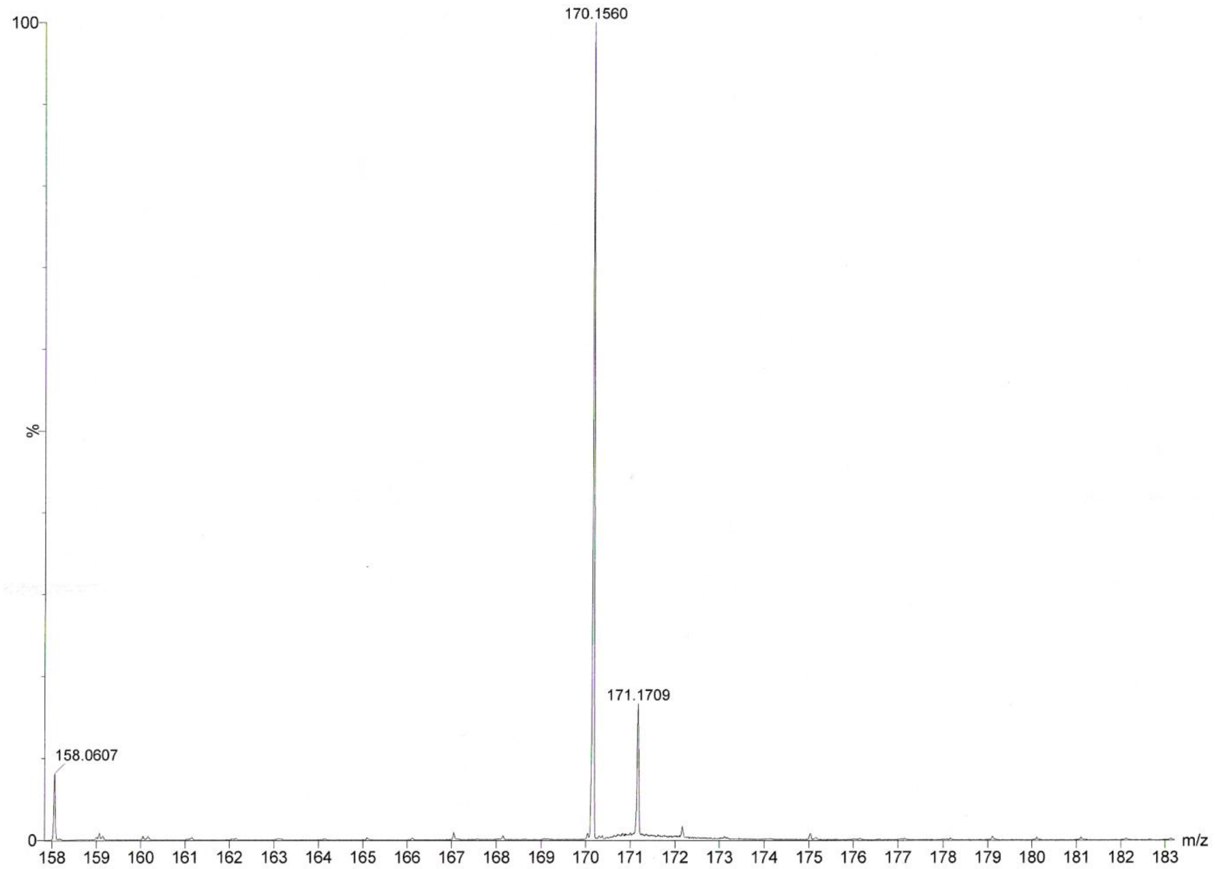
100
170.1560
%
171.1709
158.0607
0
m/z

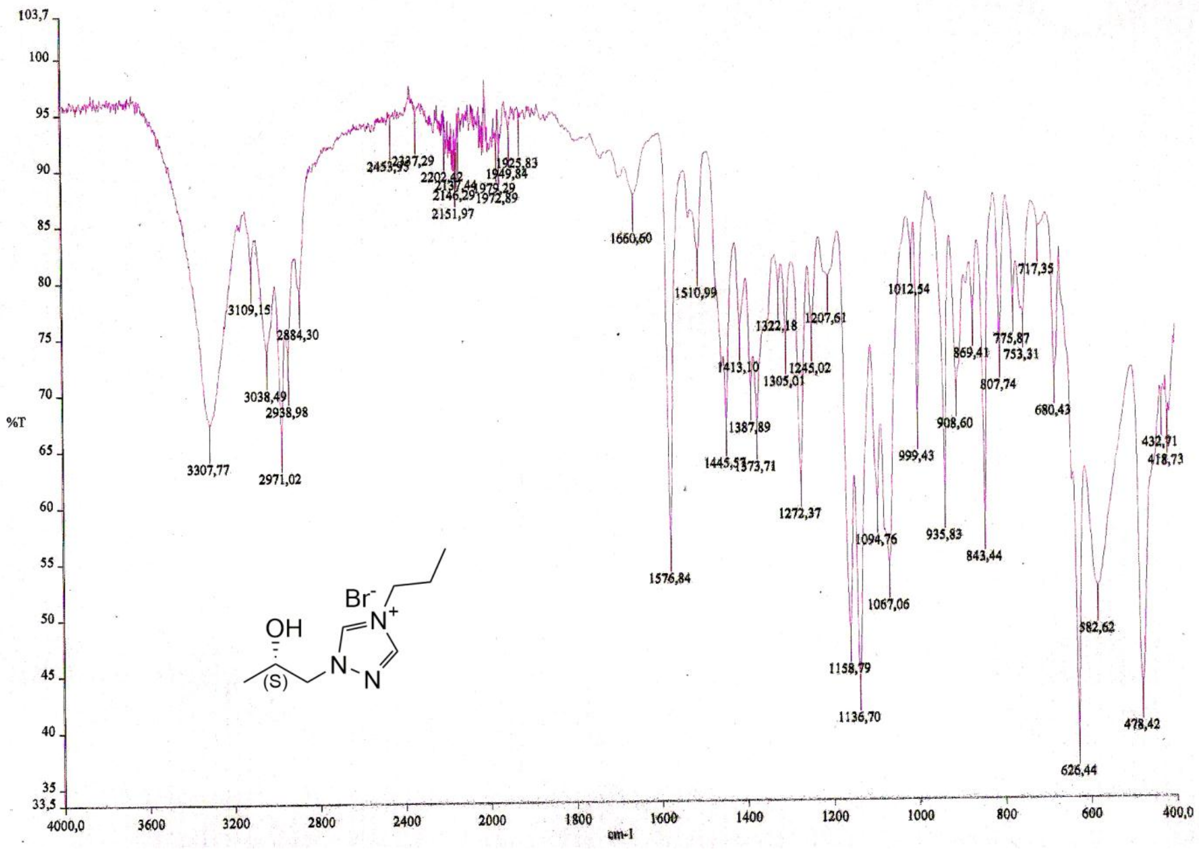

%T
cm-1
4000,0
400,0
3307,77
2971,02
1576,84
1136,70
626,44
478,42
Br⁻
OH
(S)

(*S*)-(+)-1-(2-Hydroxypropyl)-4-(prop-2-en-1-yl)-1*H*-1,2,4-triazol-4-ium bromide (+)-8b:

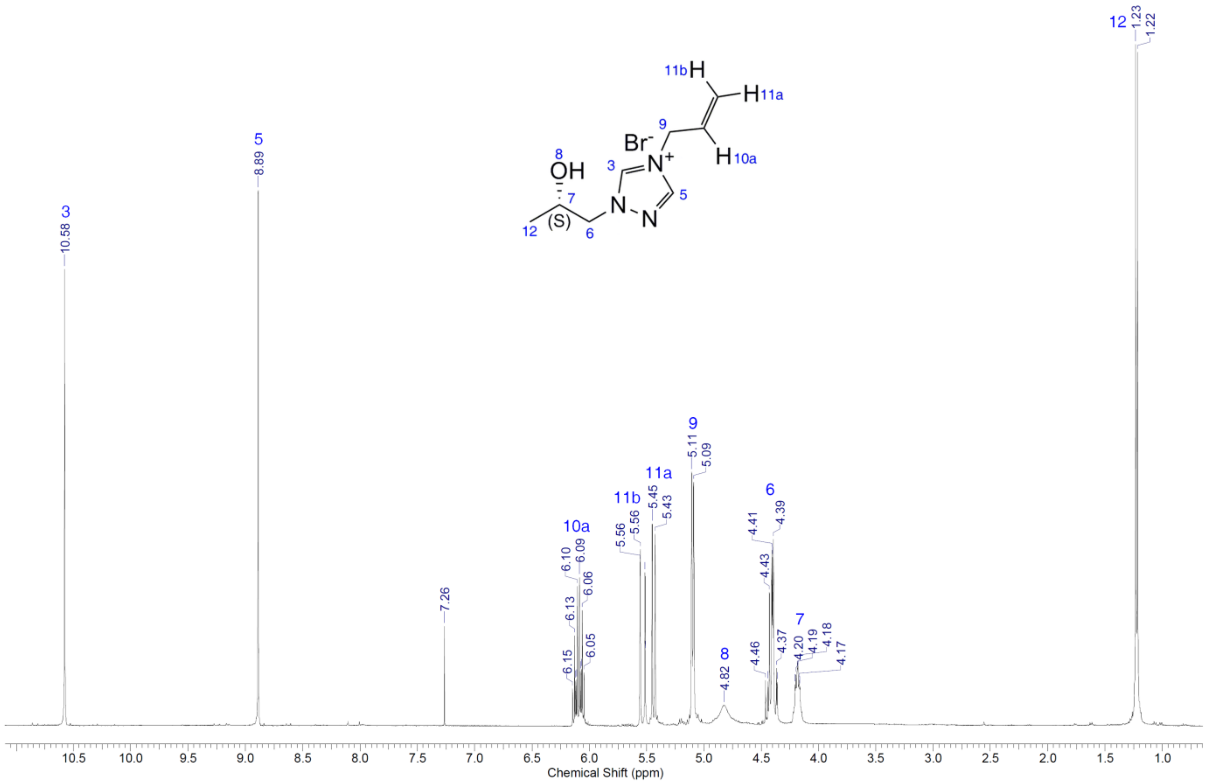

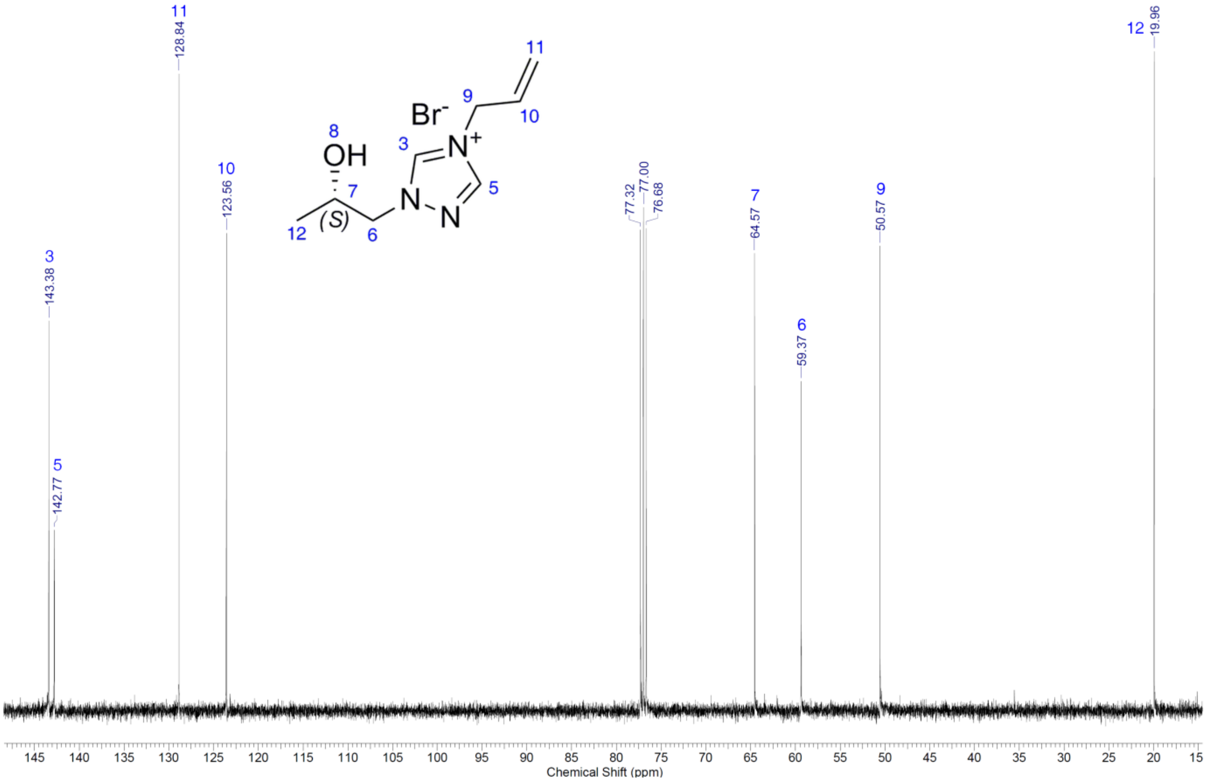

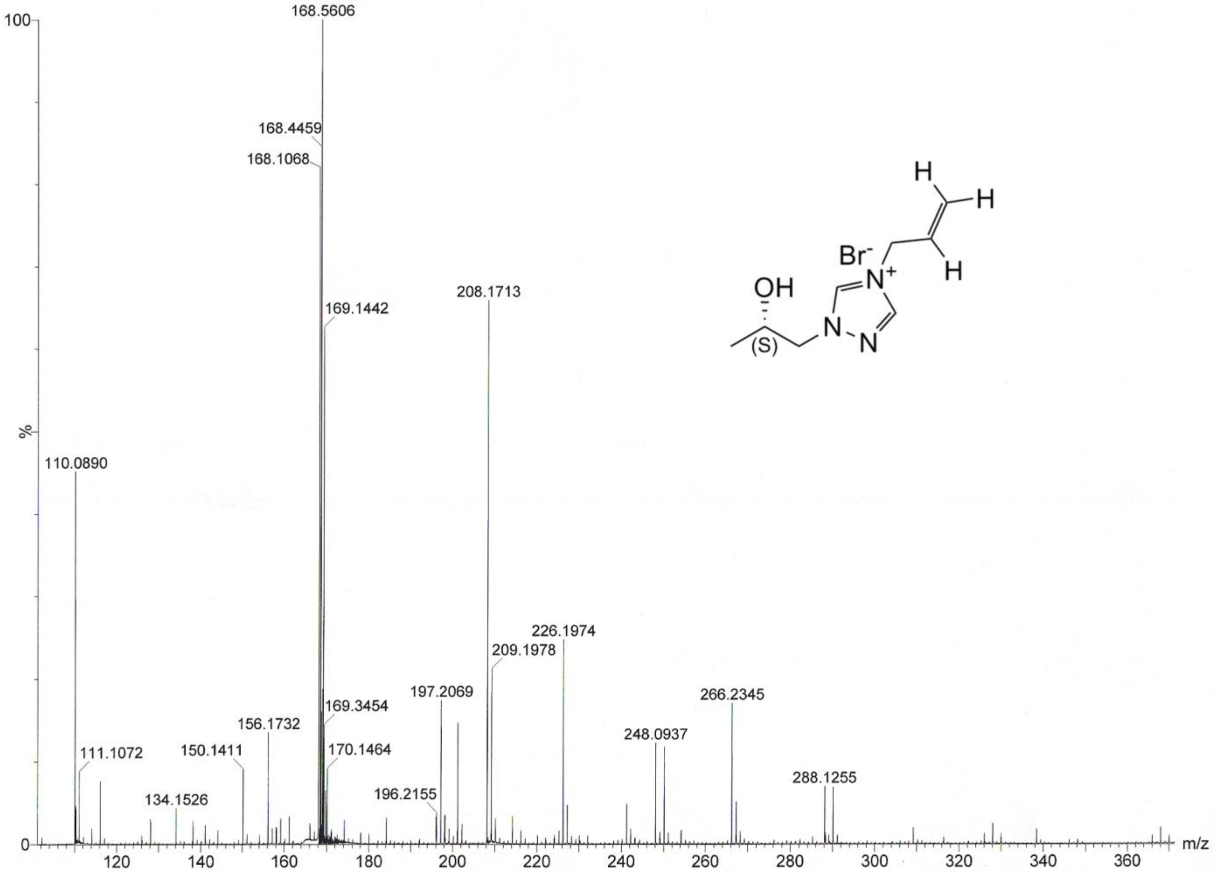

100
168.5606
168.4459
168.1068
H
H
Br-
N+
H
OH
N
N
(S)
169.1442
208.1713
%
110.0890
226.1974
209.1978
197.2069
266.2345
169.3454
156.1732
248.0937
150.1411
170.1464
111.1072
288.1255
196.2155
134.1526
0
m/z
120
140
160
180
200
220
240
260
280
300
320
340
360

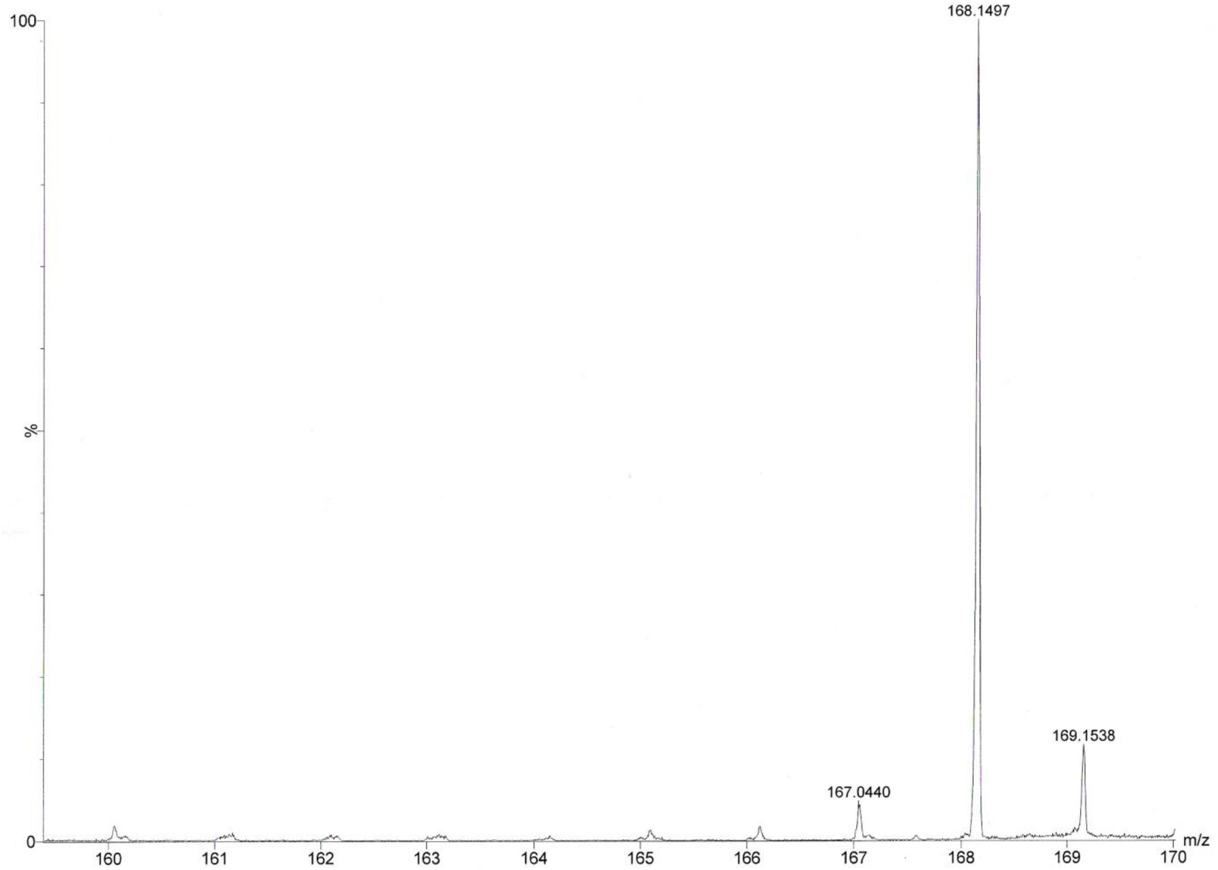

100
168.1497
%
169.1538
167.0440
0
m/z
160
161
162
163
164
165
166
167
168
169
170

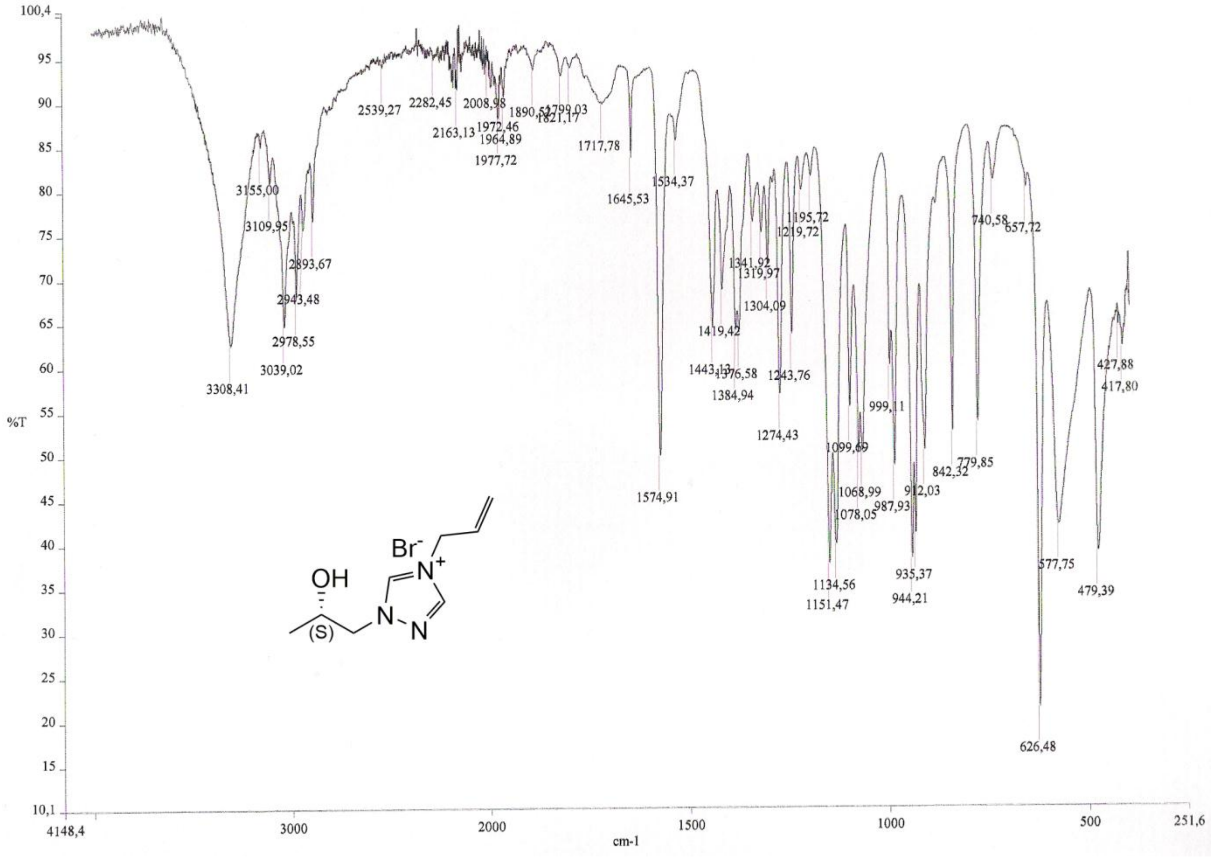
100,4
95
90
85
80
75
70
65
60
%T 55
50
45
40
35
30
25
20
15
10,1
4148,4
3000
2000
1500
1000
500
251,6
cm-1
3308,41
3155,00
3109,95
3039,02
2978,55
2943,48
2893,67
2539,27
2282,45
2163,13
2008,98
1972,46
1964,89
1977,72
1890,52
1821,17
1799,03
1717,78
1645,53
1574,91
1534,37
1443,13
1419,42
1376,58
1384,94
1341,92
1319,97
1304,09
1274,43
1243,76
1219,72
1195,72
1151,47
1134,56
1099,69
1078,05
1068,99
999,11
987,93
944,21
935,37
912,03
842,32
779,85
740,58
657,72
626,48
577,75
479,39
427,88
417,80
Br⁻
OH
(S)
N
N
N⁺

(*S*)-(+)-4-Butyl-1-(2-hydroxypropyl)-1*H*-1,2,4-triazol-4-ium bromide (+)-8c:

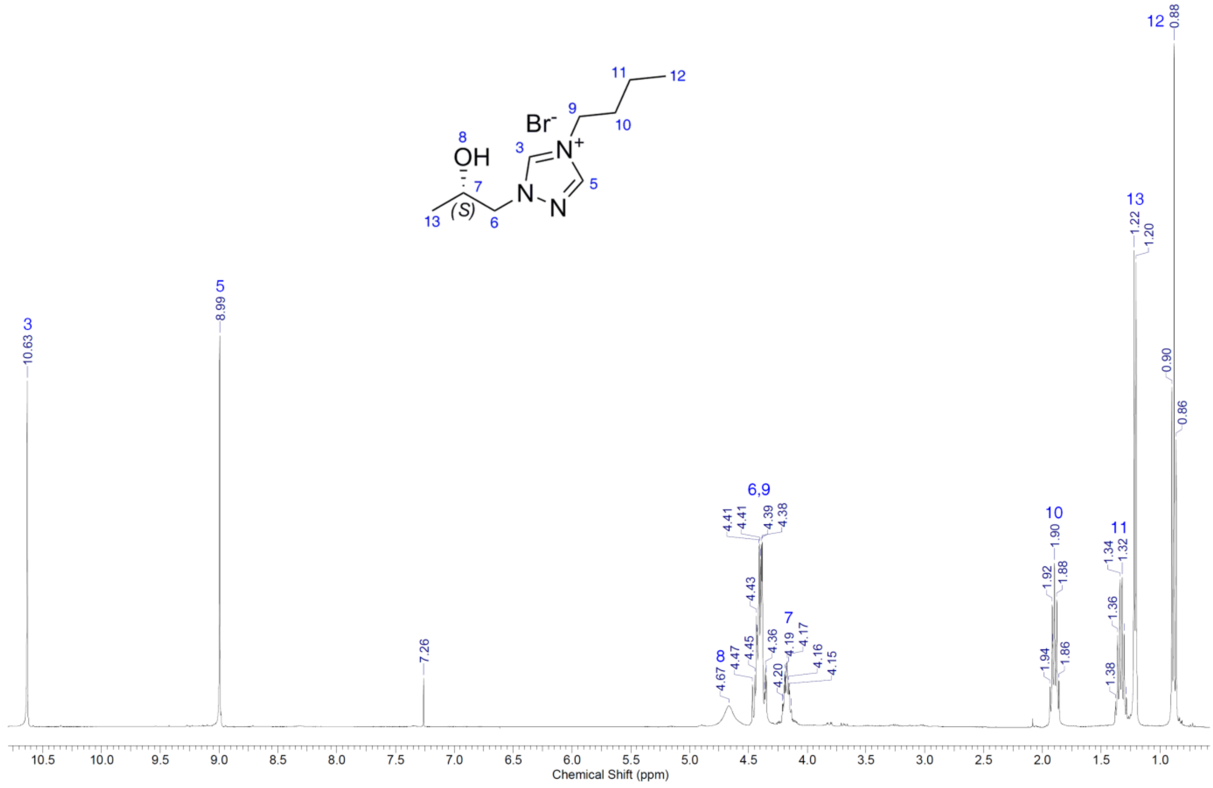

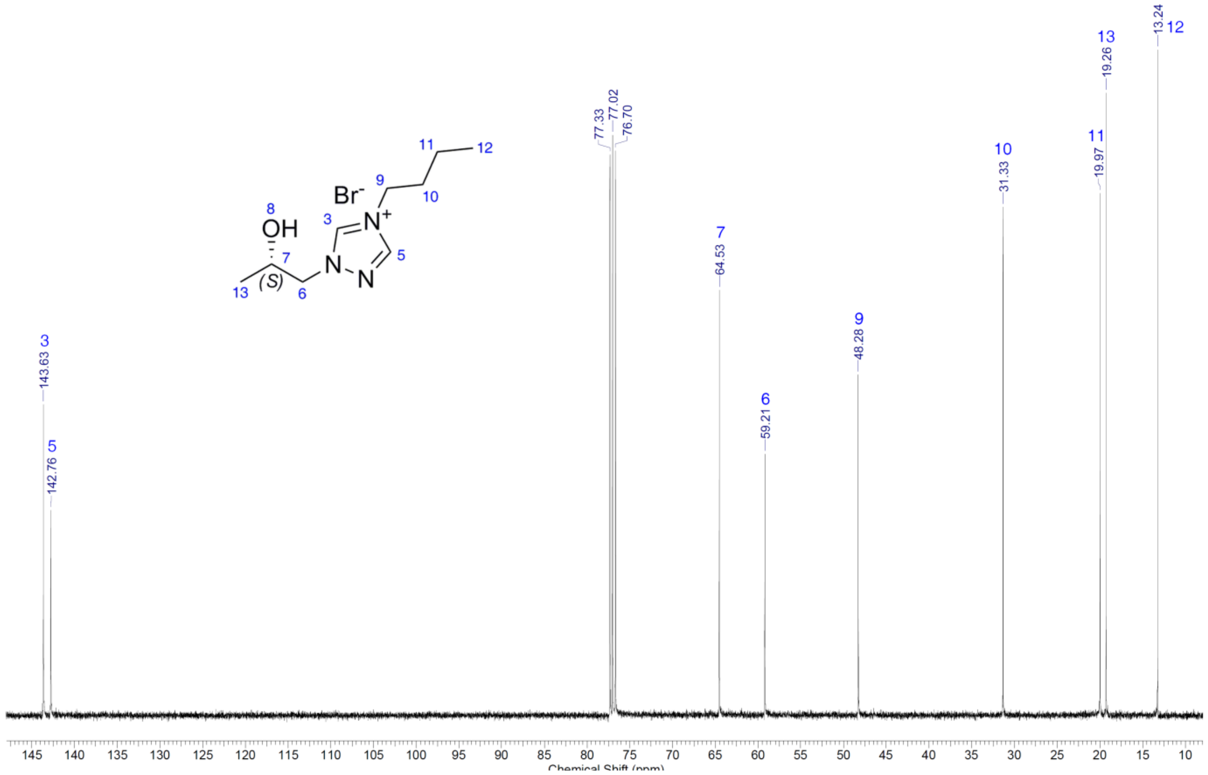

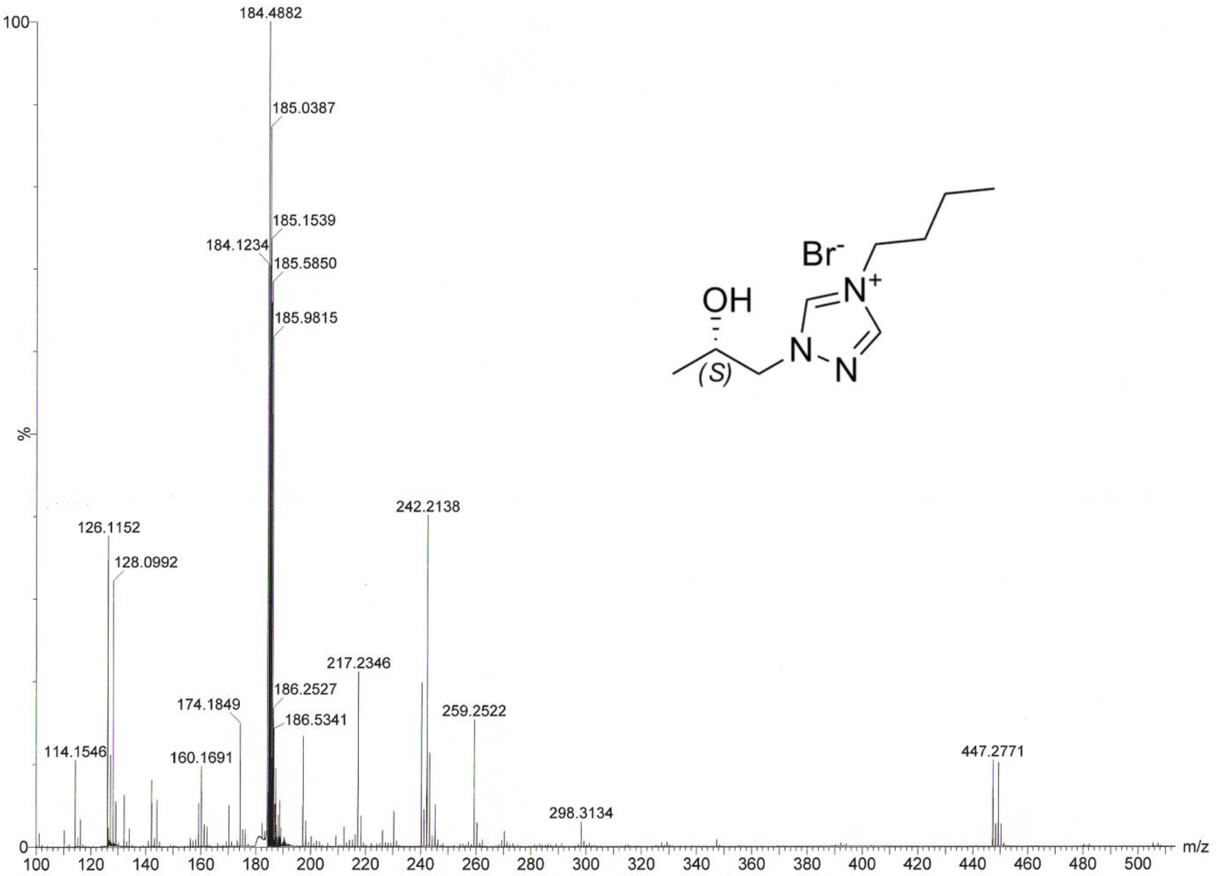

184.4882
185.0387
185.1539
184.1234
185.5850
185.9815
126.1152
128.0992
242.2138
217.2346
186.2527
174.1849
186.5341
259.2522
114.1546
160.1691
447.2771
298.3134
%
m/z
Br-
OH
(S)
N+
N
N

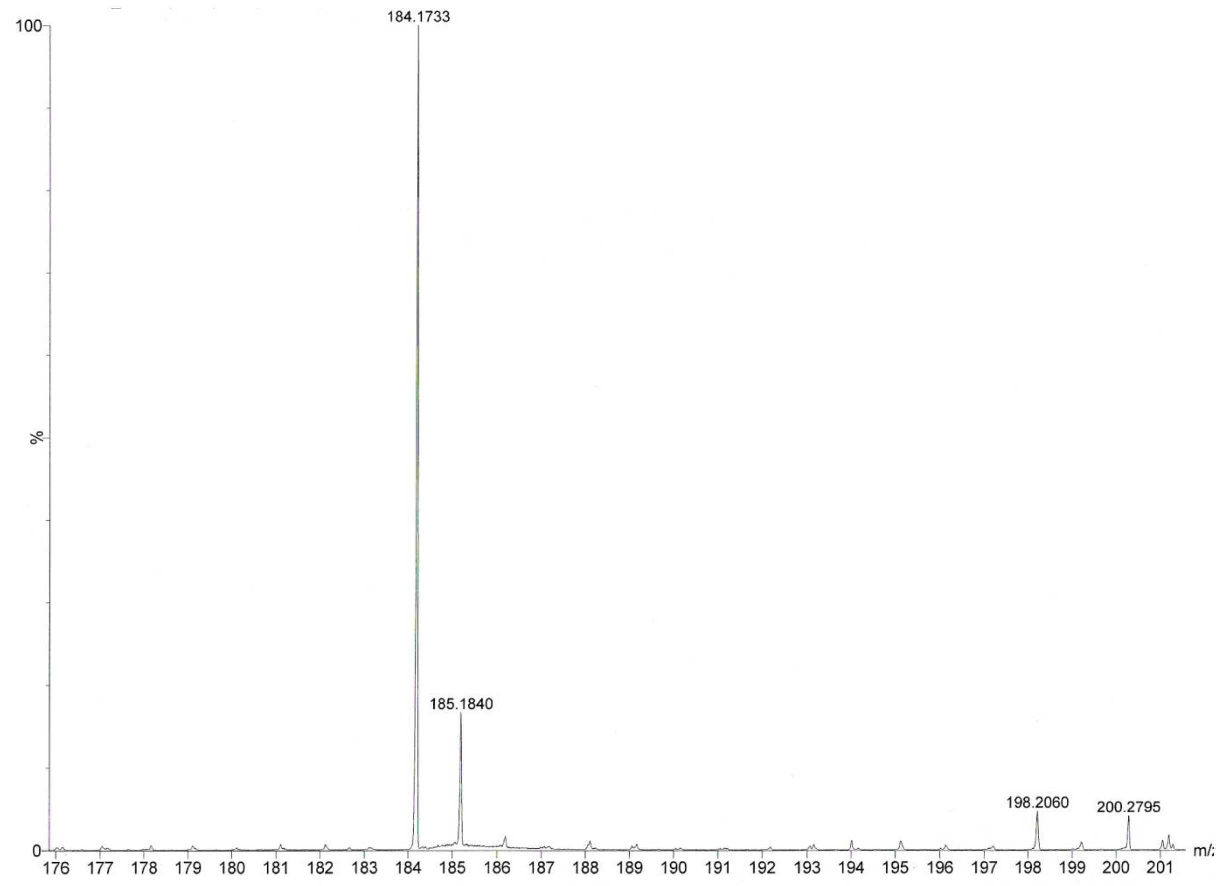

184.1733
185.1840
198.2060
200.2795
%

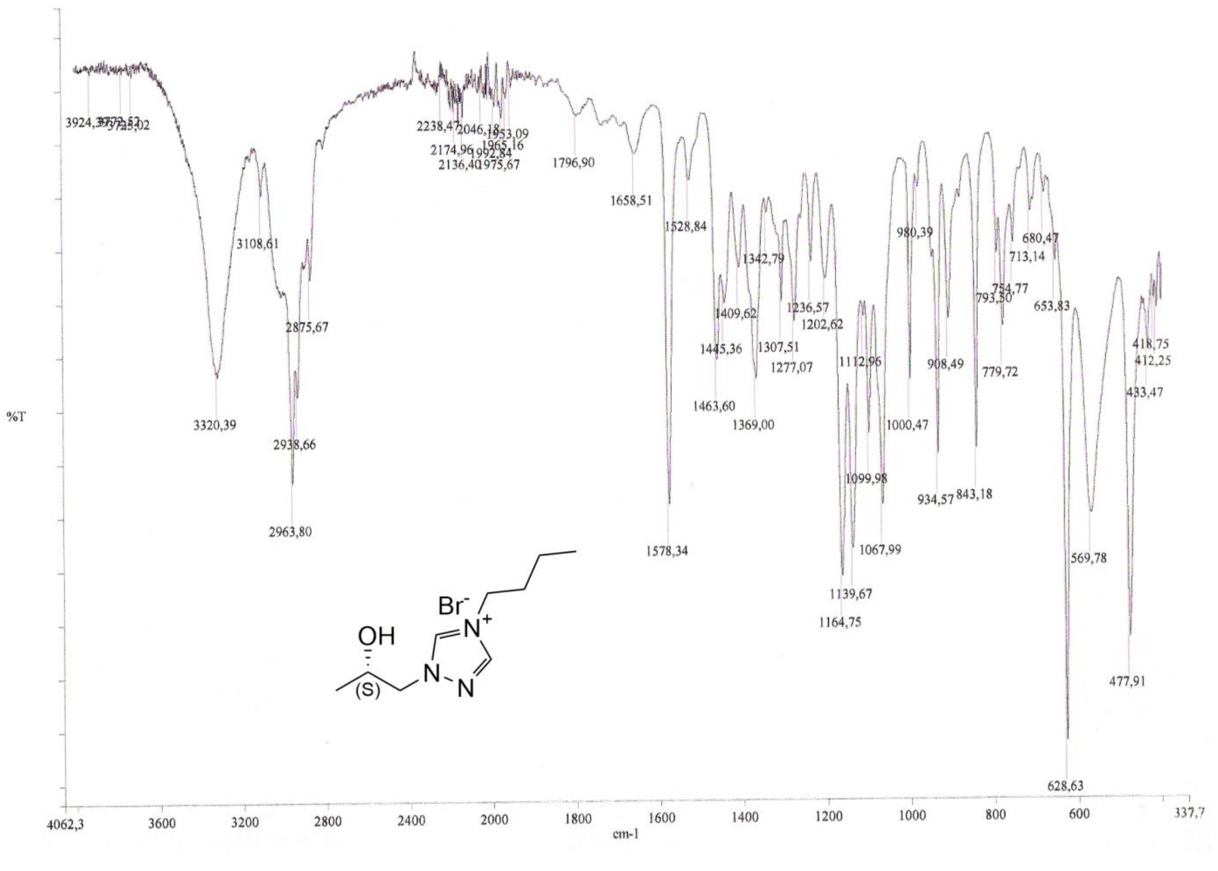
%T
cm-1
4062,3
337,7
3320,39
3108,61
2963,80
2938,66
2875,67
1796,90
1658,51
1578,34
1528,84
1463,60
1445,36
1409,62
1369,00
1342,79
1307,51
1277,07
1236,57
1202,62
1164,75
1139,67
1112,96
1099,98
1067,99
1000,47
980,39
934,57
908,49
843,18
793,50
779,72
754,77
713,14
680,47
653,83
628,63
569,78
477,91
433,47
418,75
412,25
Br-
OH
(S)
N+
N

(*S*)-(+)-1-(2-Hydroxypropyl)-4-pentyl-1*H*-1,2,4-triazol-4-ium bromide (+)-8d:

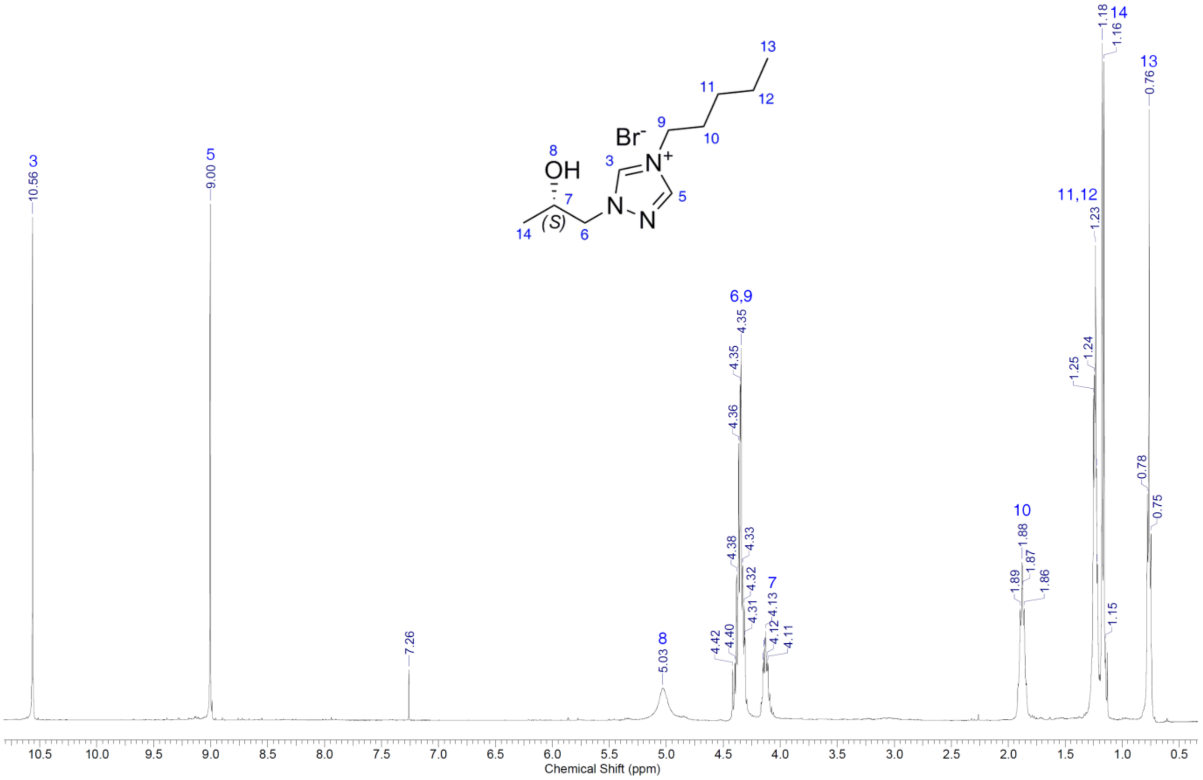

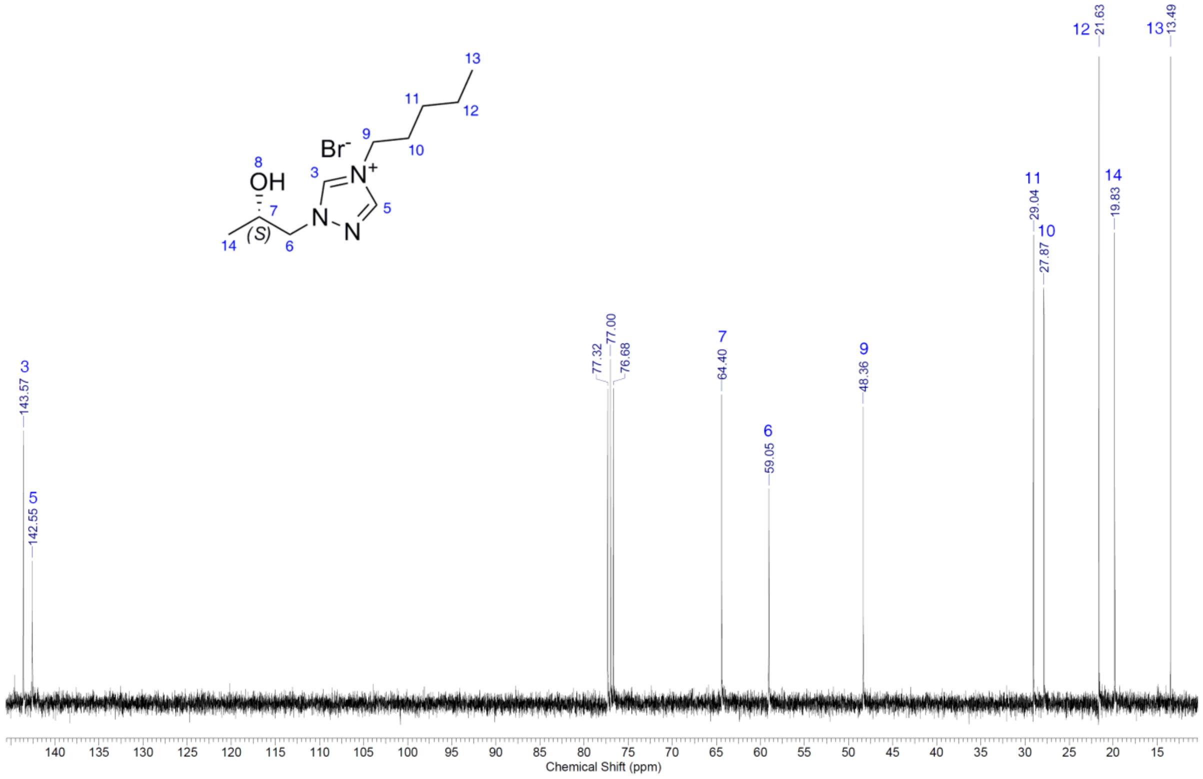

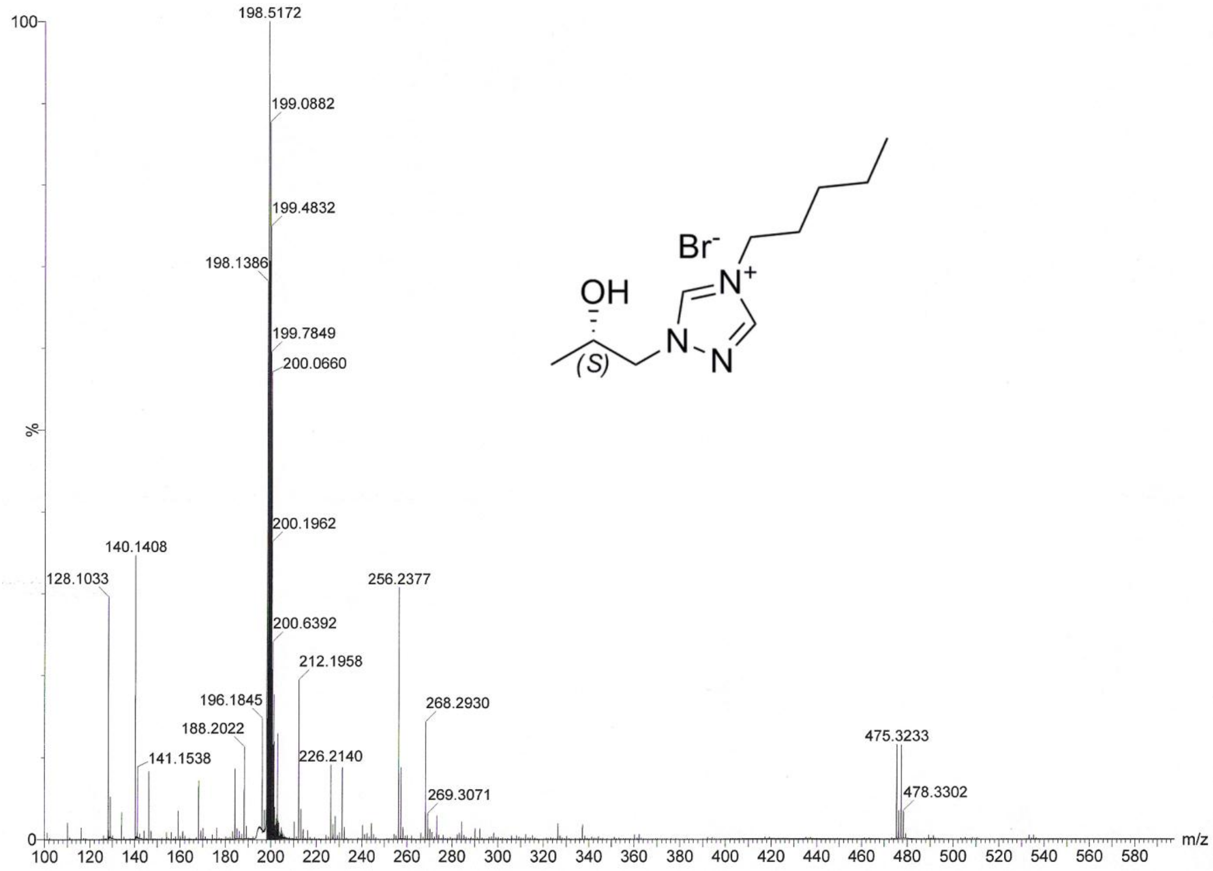
198.5172
199.0882
199.4832
198.1386
199.7849
200.0660
200.1962
140.1408
128.1033
256.2377
200.6392
212.1958
196.1845
268.2930
188.2022
141.1538
226.2140
475.3233
478.3302
269.3071
100
%
0
m/z
Br⁻
OH
N⁺
N
N
(S)

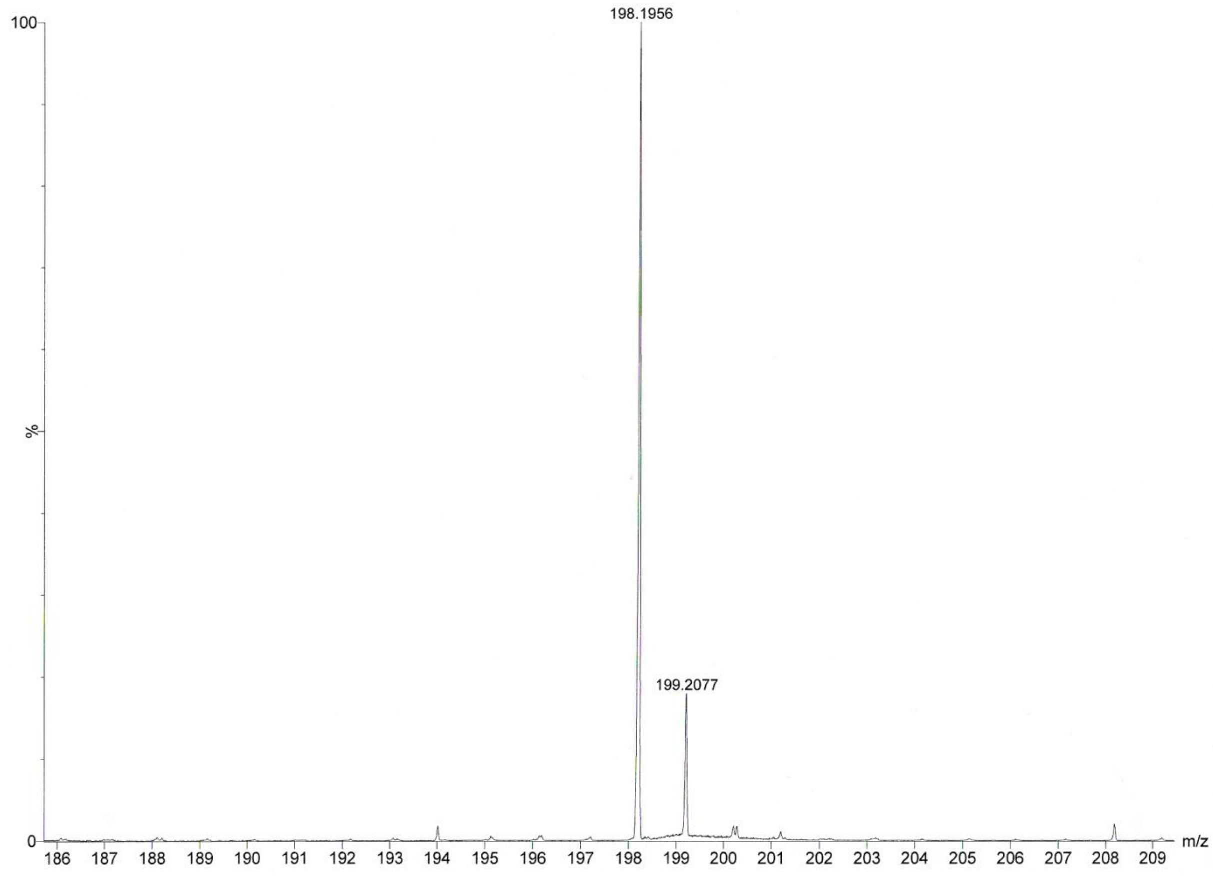
198.1956
199.2077
100
%
0
m/z

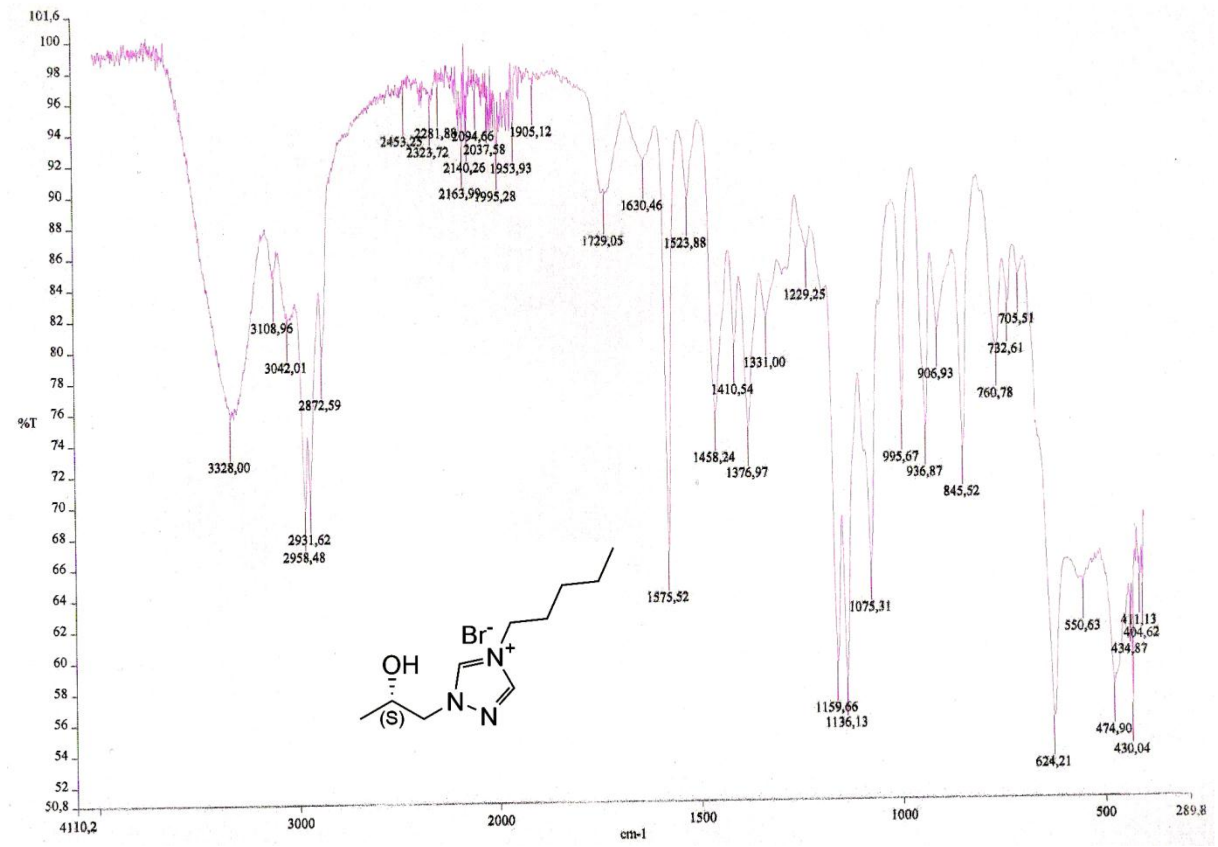
%T
cm-1
3328,00
3108,96
3042,01
2872,59
2931,62
2958,48
1729,05
1630,46
1523,88
1575,52
1458,24
1410,54
1376,97
1331,00
1229,25
1159,66
1136,13
1075,31
995,67
936,87
906,93
845,52
760,78
732,61
705,51
624,21
550,63
474,90
434,87
430,04
411,13
404,62
1905,12
OH
Br⁻
(S)
N

(*S*)-(+)-4-Heptyl-1-(2-hydroxypropyl)-1*H*-1,2,4-triazol-4-ium iodide (+)-8e:

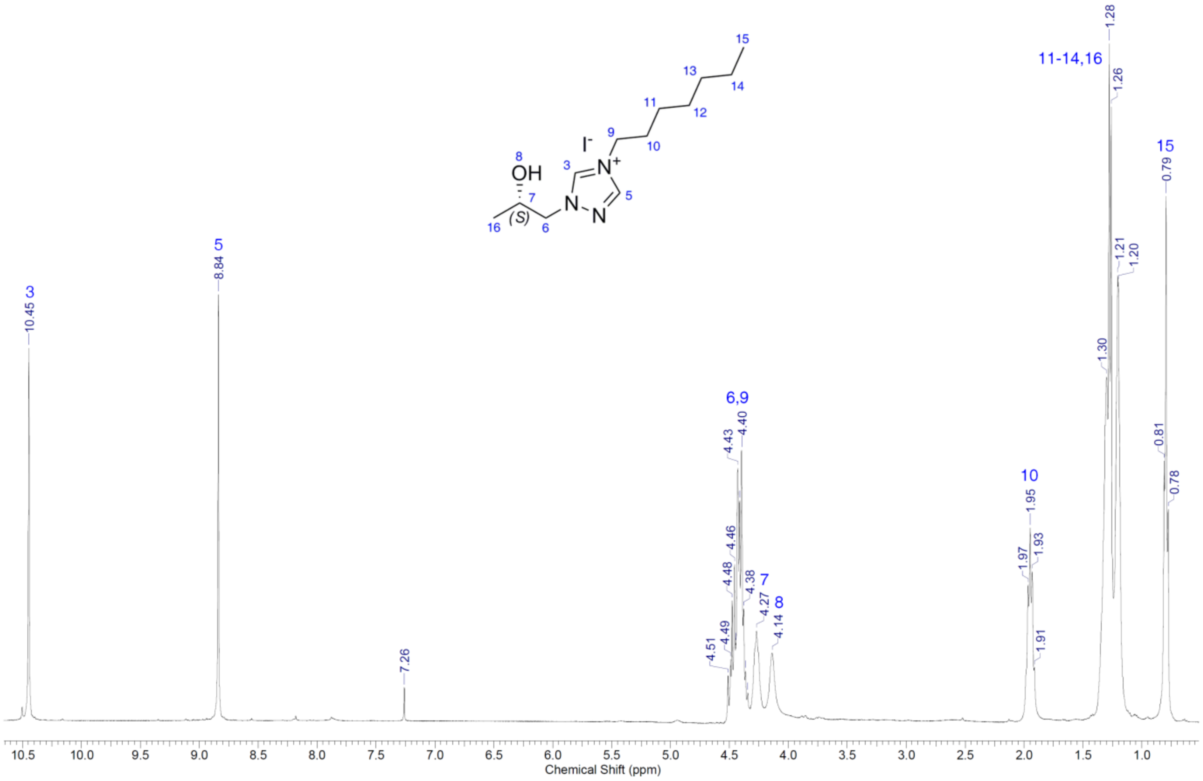

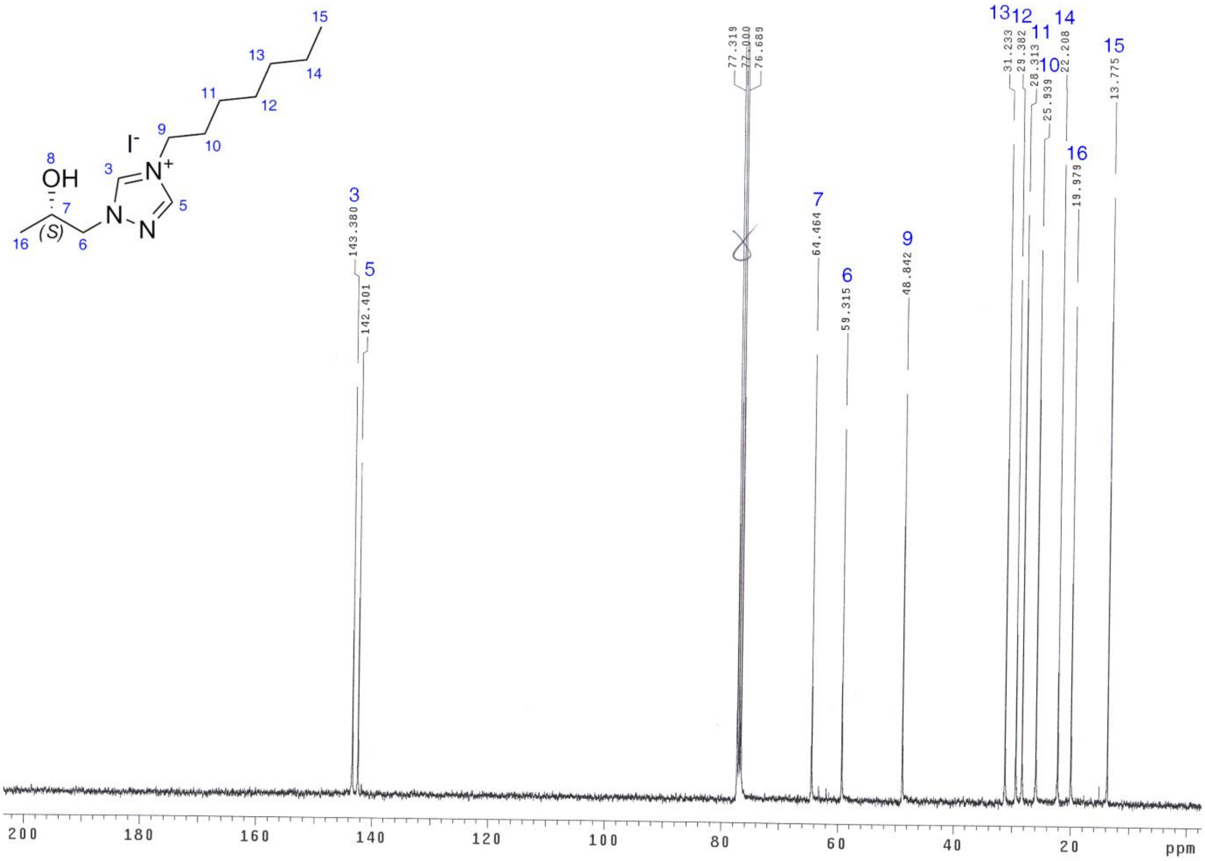

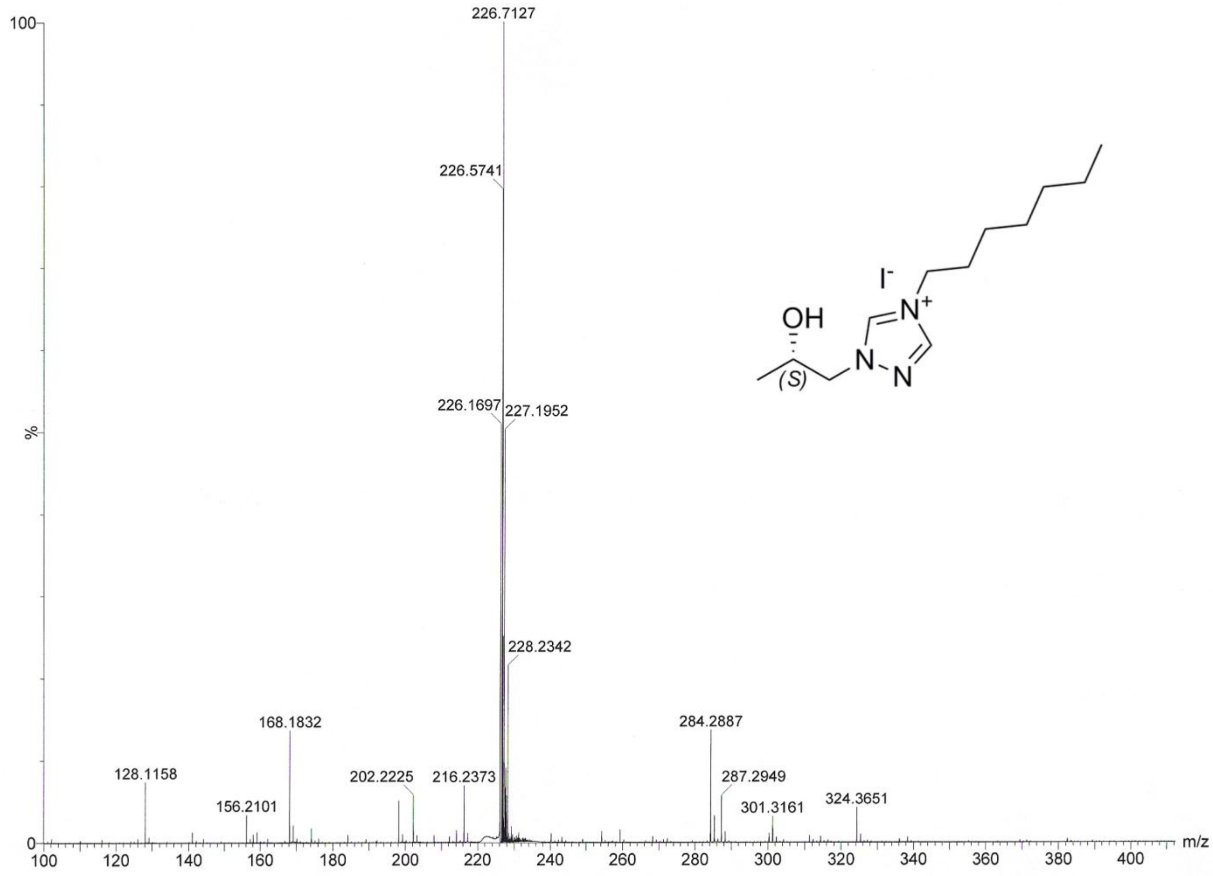
226.7127
226.5741
226.1697
227.1952
228.2342
168.1832
284.2887
128.1158
287.2949
202.2225
216.2373
156.2101
301.3161
324.3651
100
%
0
m/z
100 120 140 160 180 200 220 240 260 280 300 320 340 360 380 400
OH
(S)
N
N
N+
I-

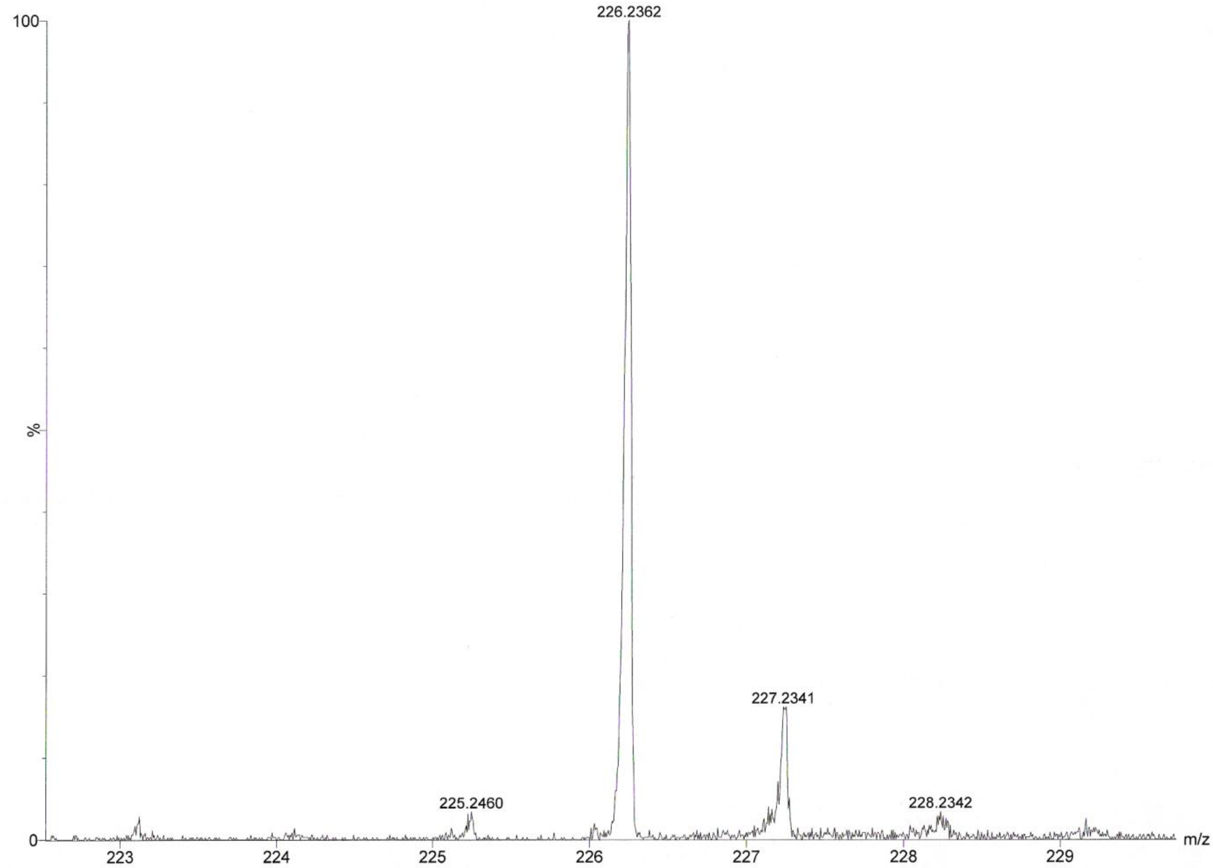
226.2362
227.2341
225.2460
228.2342
100
%
0
m/z
223 224 225 226 227 228 229

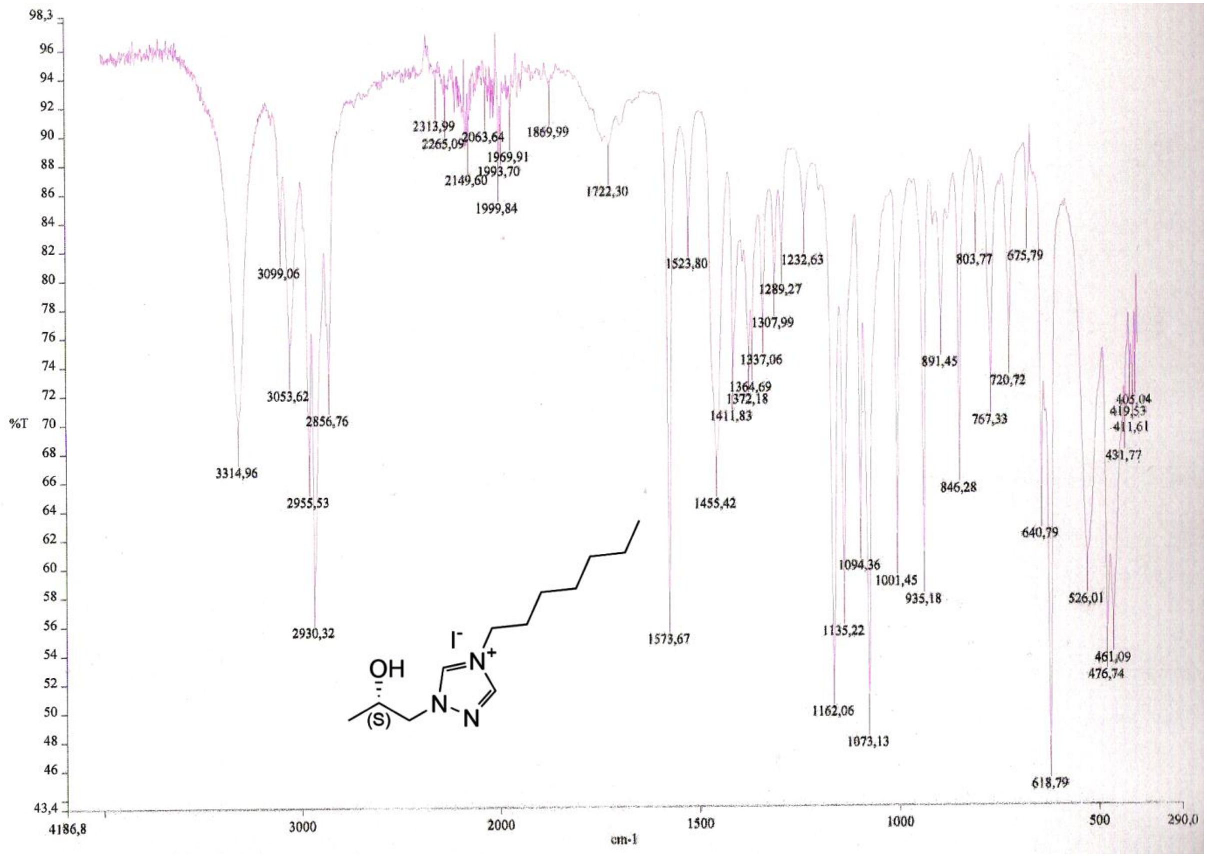
%T
cm-1
4186,8
3000
2000
1500
1000
500
290,0
98,3
43,4
3314,96
3099,06
3053,62
2955,53
2930,32
2856,76
2313,99
2265,09
2149,60
2063,64
1999,84
1993,70
1969,91
1869,99
1722,30
1573,67
1523,80
1455,42
1411,83
1372,18
1364,69
1337,06
1307,99
1289,27
1232,63
1162,06
1135,22
1094,36
1073,13
1001,45
935,18
891,45
846,28
803,77
767,33
720,72
675,79
640,79
618,79
526,01
476,74
461,09
431,77
419,53
411,61
405,04
OH
(S)
I-
N+

(*S*)-(+)-4-Decyl-1-(2-hydroxypropyl)-1*H*-1,2,4-triazol-4-ium iodide (+)-8f:

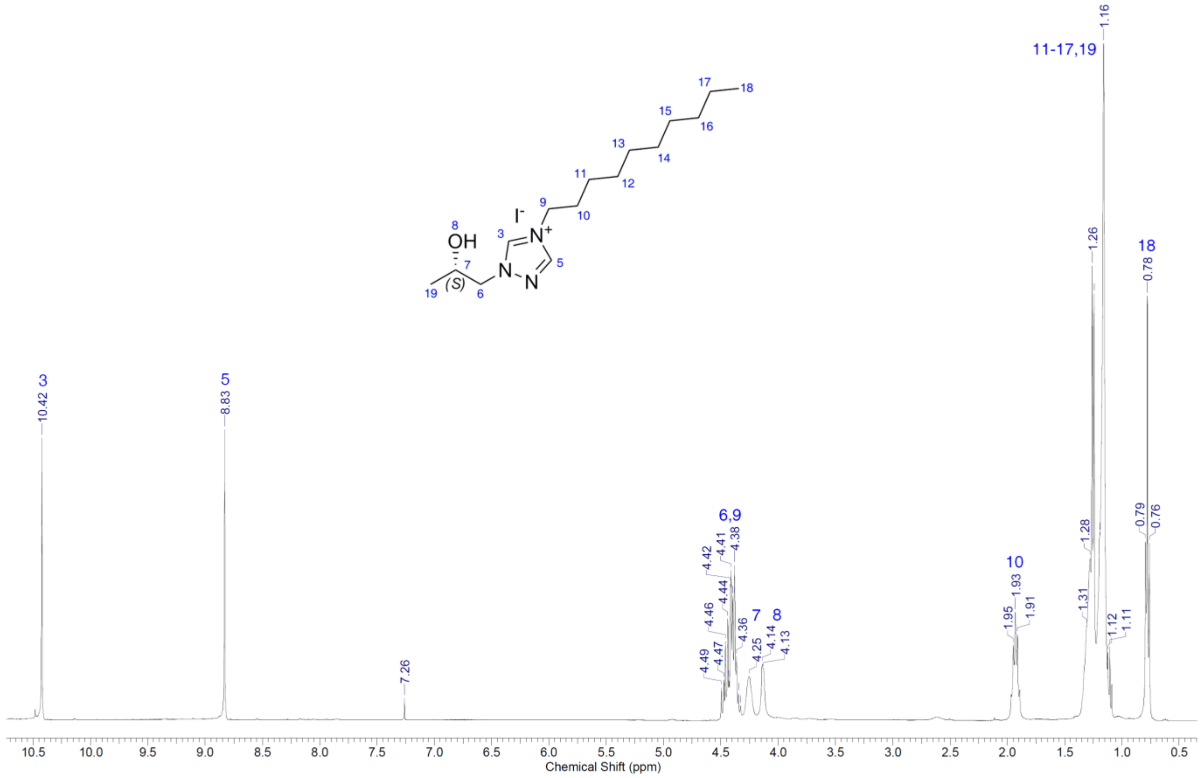

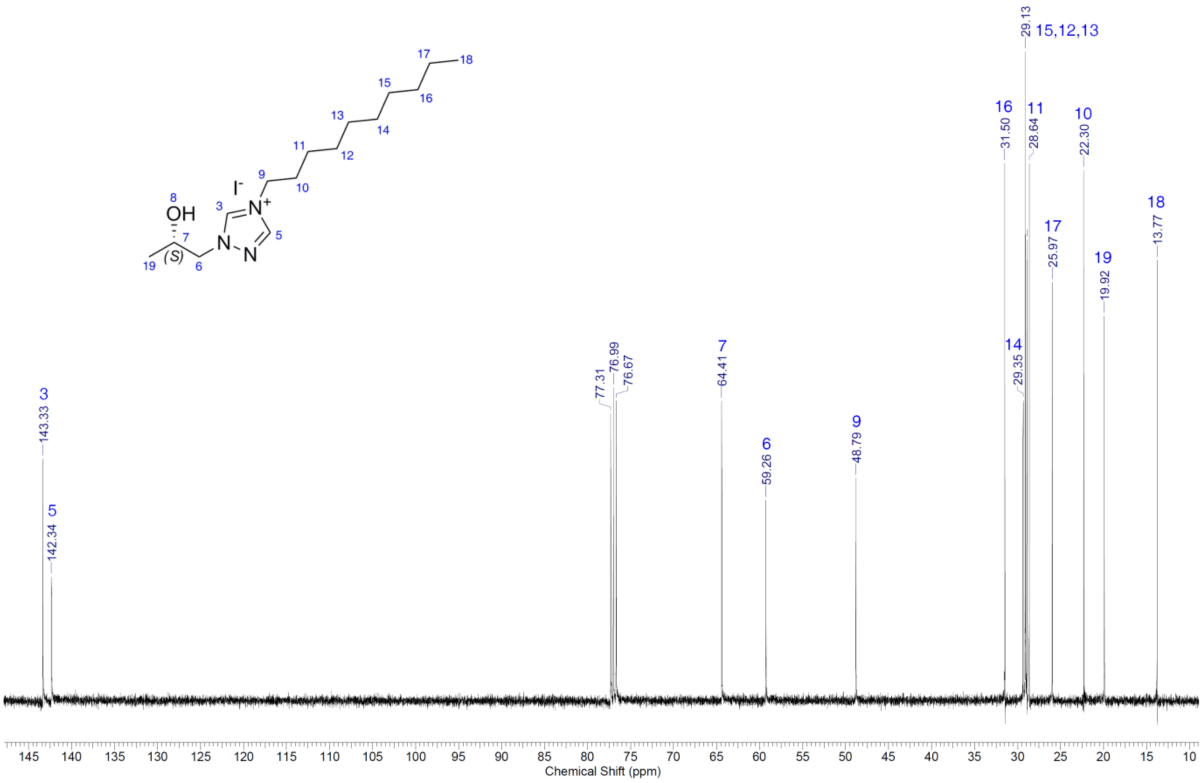

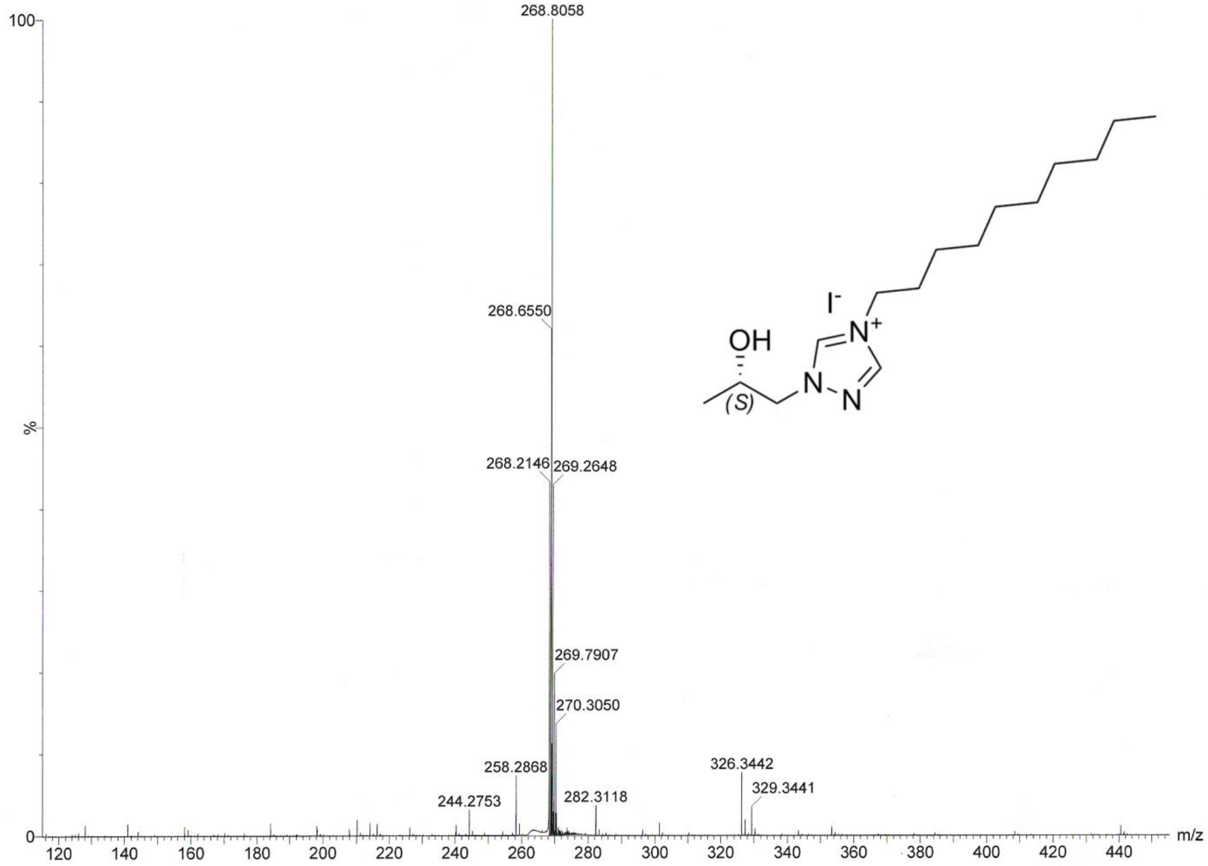

268.8058
268.6550
268.2146
269.2648
269.7907
270.3050
258.2868
244.2753
282.3118
326.3442
329.3441
100
%
0
m/z
120 140 160 180 200 220 240 260 280 300 320 340 360 380 400 420 440
I⁻
OH
N
N⁺
N
(S)

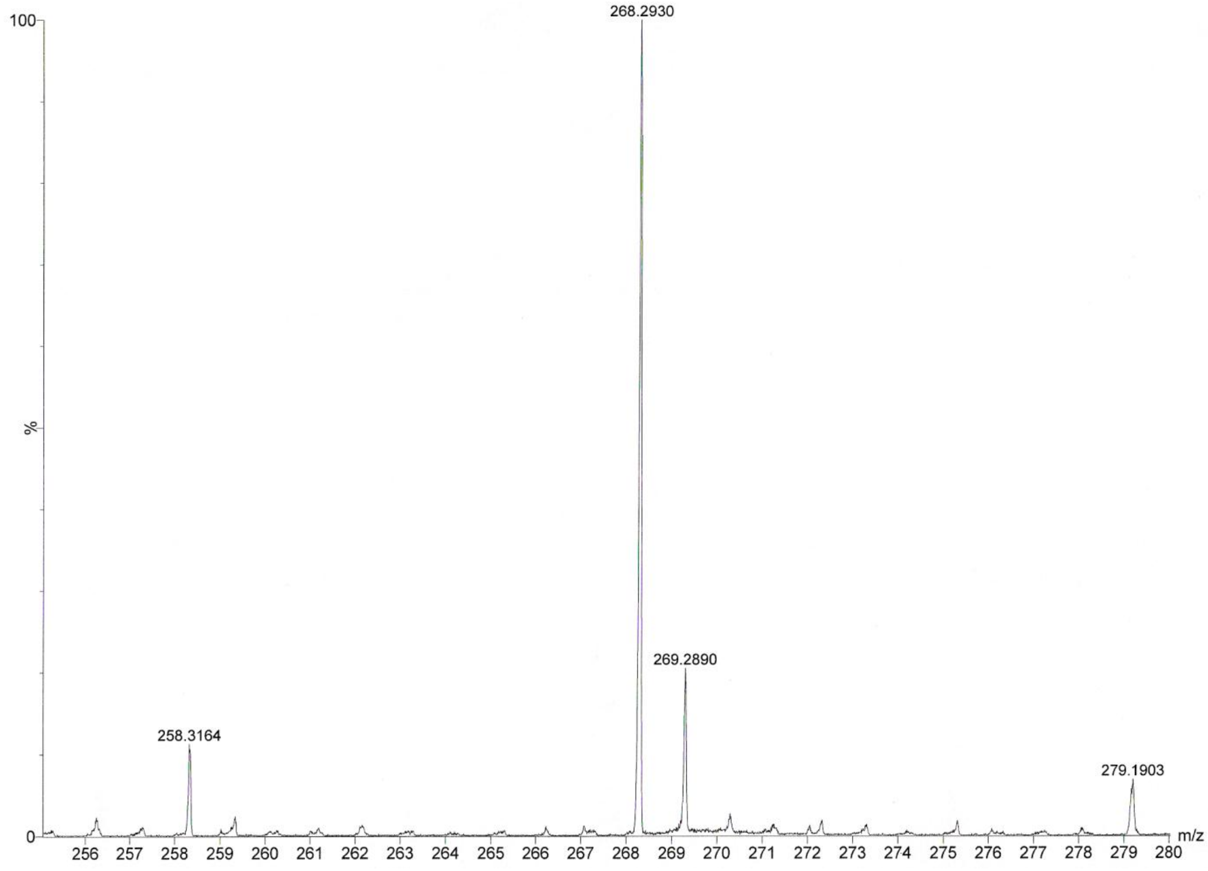

268.2930
269.2890
258.3164
279.1903
100
%
0
m/z
256 257 258 259 260 261 262 263 264 265 266 267 268 269 270 271 272 273 274 275 276 277 278 279 280

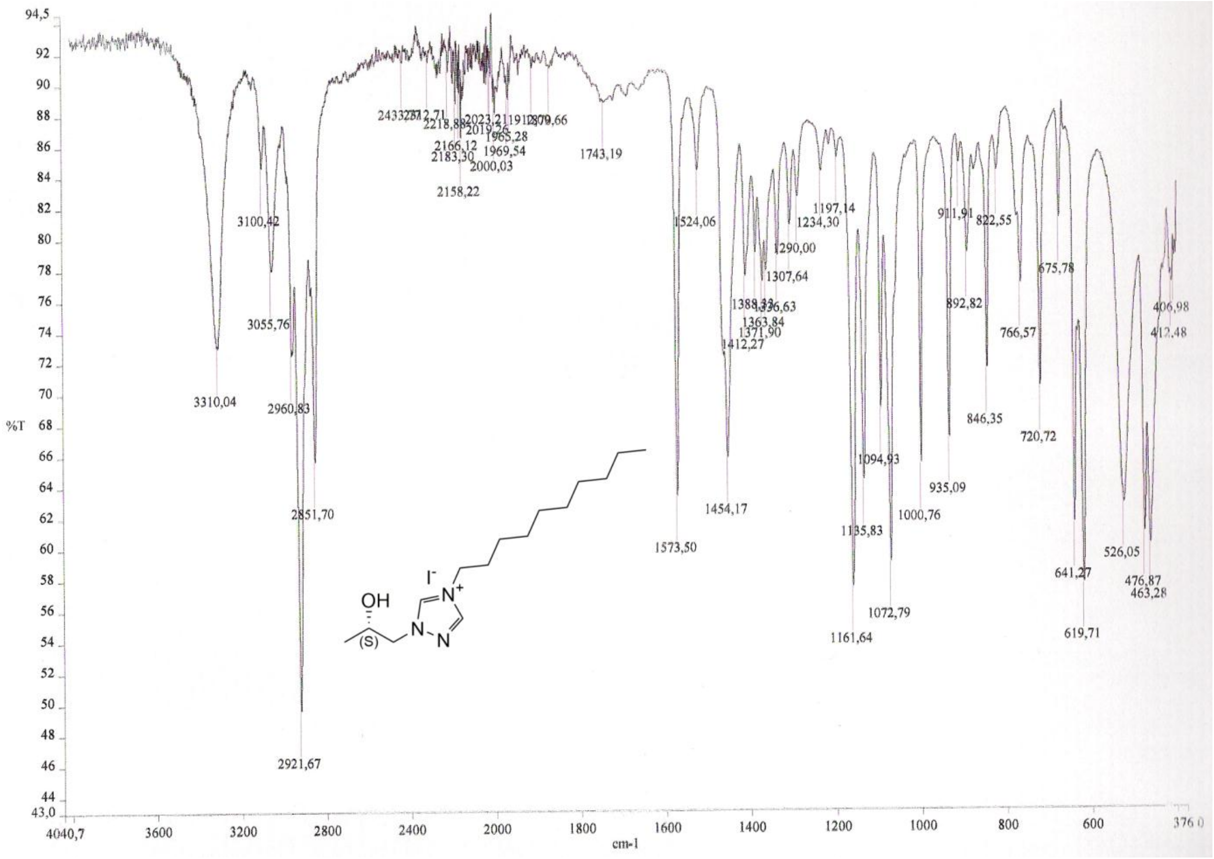

References

1. Rebros, M.; Gunaratne, H. Q. N.; Ferguson, J.; Seddon, K. R.; Stephens, G. *Green Chem.* **2009,** *11*, 402-408.

2. Jorgensen, J. H.; Ferraro, M. J. *Clin. Infect. Dis*. **2009,** *49*, 1749-1755.

3. Liu, W.; Shi, H. M.; Jin, H.; Zhao, H. Y.; Zhou, G. P.; Wen, F.; Yu, Z. Y.; Hou, T. P. *Chem. Biol. Drug. Des*. **2009,** *73*, 661-667.

4. Medina, M.; Platt, H. *Am. J. Potato Res*. **1999,** *76*, 121-125.
